# Supplementary material for: Cardiovascular magnetic resonance in emergency patients with multivessel disease or unobstructed coronary arteries: a cost-effectiveness analysis in the UK
Source: BMJ Open. 2019 Jul 11;9(7):e025700. doi: 10.1136/bmjopen-2018-025700 (PMC6629389; doi:10.1136/bmjopen-2018-025700)
Supplement: Supplementary file 1 [file bmjopen-2018-025700supp001.pdf]

SUPPLEMENTARY INFORMATION

Table of Contents

Appendix A – Model parameters, sources and assumptions for model 1 (multi-vessel disease) 2

Appendix B – Model parameters, sources and assumptions for model 2 (unobstructed coronary arteries) 6

Appendix C – Deterministic one-way sensitivity analyses for model 1 (multi-vessel disease) 9

Appendix D – Deterministic one-way sensitivity analyses for model 2 (unobstructed coronary arteries) 11

References 12

APPENDIX A – MODEL PARAMETERS, SOURCES AND ASSUMPTIONS FOR MODEL 1 (MULTI-VESSEL DISEASE)

**Testing and clinical parameters**			
Branch of model	Base-case value (Positive/total)	Distribution for PSA	Source
For each ischaemia testing option			
Probability index angiogram leads to revascularisation of second vessel	0.30 (30/100)*	Dirichlet	Expert opinion within the PIPA study team
Probability of uncertainty following index angiogram and need for ischaemia testing	0.60 (60/100)*		
Probability index angiogram leads to no revascularisation of second vessel	0.10 (10/100)*		
Index angiogram leads to revascularisation of second vessel			
Probability of true ischaemia	0.84 (598/709)	Beta	FAME[1]
Probability of MACE by 12 months | true ischaemia	0.09 (18/202)†	Beta	Smits 2017[2]
Probability of MACE by 12 months | truly no ischaemia	0.13 (54/432)†	Beta	Smits 2017[2]
Uncertainty following index angiogram and need for ischaemia testing			
Probability of true ischaemia	0.35 (218/620)	Beta	FAME[1]
Index angiogram leads to no revascularisation of second vessel			
Probability of true ischaemia	0.35 (218/620)	Beta	FAME[1]
Probability of MACE by 12 months | true ischaemia	0.31 (71/231)†	Beta	Smits 2017[2]
Probability of MACE by 12 months | truly no ischaemia	0.13 (54/432)†	Beta	Smits 2017[2]
CMR or FFR			
Uncertainty following index angiogram and need for ischaemia testing, True ischaemia			
Probability of ischaemia test positive and revascularisation (sensitivity of test)	1§	Beta	
Probability of MACE by 12 months | revascularisation	0.09 (18/202)†	Beta	Smits 2017[2]
Probability of MACE by 12 months | no revascularisation	0.31 (71/231)†#	Beta	Smits 2017[2]
Uncertainty following index angiogram and need for ischaemia testing, Truly no ischaemia			
Probability of ischaemia test positive and revascularisation (1 minus specificity of test)	0§	Beta	
Probability of MACE by 12 months | revascularisation	0.13 (54/432)†#	Beta	Smits 2017[2]

Probability of MACE by 12 months | no revascularisation	0.13 (54/432)†	Beta	Smits 2017[2]
Stress ECHO			
Uncertainty following index angiogram and need for ischaemia testing, True ischaemia			
Probability of ischaemia test positive and revascularisation (sensitivity of test)	0.70 (32/46)	Beta	Gurunathan 2016[3]
Probability of MACE by 12 months | revascularisation	0.09 (18/202)†	Beta	Smits 2017[2]
Probability of MACE by 12 months | no revascularisation	0.31 (71/231)†	Beta	Smits 2017[2]
Uncertainty following index angiogram and need for ischaemia testing, Truly no ischaemia			
Probability of ischaemia test positive and revascularisation (1 minus specificity of test)	0.23 (39/171)	Beta	Gurunathan 2016[3]
Probability of MACE by 12 months | revascularisation	0.13 (54/432)†	Beta	Smits 2017[2]
Probability of MACE by 12 months | no revascularisation	0.13 (54/432)†	Beta	Smits 2017[2]

**Cost parameters**			
Resource	Unit cost (SE)**	Distribution for PSA	Source
Revascularisation of second vessel (assumed to be PCI)	£2,682 (533)	Gamma	NHS Reference Costs 2015/16.[4] Elective Inpatient. Weighted average of codes EY40 and EY41, Standard / Complex Percutaneous Transluminal Coronary Angioplasty.
CMR	£264 (154)	Gamma	NHS Reference Costs 2015/16.[4] Diagnostic Imaging. Weighted average of all Cardiac Magnetic Resonance Imaging Scan codes.
Angiogram and FFR	£1,340 (374)	Gamma	NHS Reference Costs 2015/16.[4] Day case. Weighted average of code EY42, Complex Cardiac Catheterisation.
Angiogram, FFR and revascularisation (as a single catheter laboratory admission)	£2,971 (594)	Gamma	Cost of revascularisation above, and angiogram and FFR above, minus the cost of an angiogram, £1,051 (NHS Reference Costs 2015/16.[4] Day case. Weighted average of code EY43, Standard Cardiac Catheterisation)
Stress ECHO	£182 (3)	Gamma	NHS Reference Costs 2015/16.[4] Outpatient procedures. Cardiology. EY50Z, Complex Echocardiogram.
MACE	£3,251 (650)	Gamma	The proportion of each component of MACE was taken from Smits, 2017,[2] and a weighted average of the costs of

			each component was calculated, based on the unit costs below.
Revascularisation (PCI)	£2,682		As above
Revascularisation (CABG)	£10,944		NHS Reference Costs 2015/16.[4] Elective Inpatient. Weighted average of codes ED26, ED27, ED28 Complex/ Major/ Standard Coronary Artery Bypass Graft
Myocardial infarction (MI)	£2,177		NHS Reference Costs 2015/16.[4] Non-elective long stay. Weighted average of code EB10A, Actual or Suspected Myocardial Infarction.
Stroke	£3,723		NHS Reference Costs 2015/16.[4] Non-elective long stay. Weighted average of code AA22, Cerebrovascular Accident, Nervous System Infections or Encephalopathy.
Death	£0		
Cardiac rehabilitation	£364 (73)	Gamma	Assume offered eight sessions, but only 50% update, so cost four sessions (one first and three follow up appointments, individual costs below)
First appointment	£97		NHS Reference Costs 2015/16.[4] Consultant Led. 327, Cardiac Rehabilitation. Non-Admitted Face to Face Attendance, First.
Follow up appointment	£89		NHS Reference Costs 2015/16.[4] Consultant Led. 327, Cardiac Rehabilitation. Non-Admitted Face to Face Attendance, Follow-Up.
Cardiac medications for treatment of MI (all once daily by mouth). Assumed given for 12 months (or 6 months for patients who die).			
Aspirin (75mg)	£0.04		BNF 73, March 2017[5]
Prasugrel (5mg)	£1.70		BNF 73, March 2017[5]
Atorvastatin (80mg)	£0.07		BNF 73, March 2017[5]
Bisoprolol (2.5mg)	£0.02		BNF 73, March 2017[5]
Ramipril (2.5mg)	£0.04		BNF 73, March 2017[5]
Total daily cost	£1.87		
Total annual cost	£683 (137)	Gamma	
Outpatient follow up appointment (assumed to occur at 4-6 weeks and 6 months post-discharge)	£122 (22)	Gamma	NHS Reference Costs 2015/16.[4] Consultant led appointments. Non-Admitted Face to Face Attendance, Follow-Up. Cardiology.
Additional healthcare costs to one year	£2221 (444)	Gamma	Office of Health Economics estimate of the annual cost per person of NHS care inflated to 2015/16 prices using the

			hospital and community health services inflation index[6, 7] For patients who die, costs are assumed to be half of this (£1111).
Additional healthcare costs to one year for those with MACE	£263 (53)	Gamma	MI and stroke were assumed to be associated with continuing care costs post discharge. Costs from Greenhalgh (2011),[8] were inflated to 2015/16 prices.[6] Separate costs were provided for disabling and non-disabling stroke; according to Davies (2006) 58% of strokes are disabling,[9] and this percentage was used to weight the two continuing care costs for stroke. Additional costs to one year associated with MACE were calculated by weighting estimates of the annual costs of MI and stroke by the number of patients with these complications across the number of patients with MACE, from Smits (2017).[2]
**Quality-adjusted life years**			
Patient group	Mean (SE) †† QALYs to one year	Distribution for PSA	Source
No revascularisation of a second vessel and MACE	0.686 (0.09)	Beta	Calculations and sources described in the text
No revascularisation of a second vessel and no MACE	0.768 (0.09)	Beta	
Revascularisation of a second vessel and MACE	0.684 (0.09)	Beta	
Revascularisation of a second vessel and no MACE	0.766 (0.09)	Beta	

* To incorporate uncertainty around the percentage of patients in each of these three groups estimated by experts on the study team, it was assumed that experts were estimating the number of patients out of 100 in each group.
† For further information on the estimates of MACE, please see the final paragraph under ‘Model probabilities’.
§ In the base case analyses, CMR and FFR are assumed to have perfect sensitivity and specificity, but these parameters are incorporated into the model so that alternative assumptions can be tested in sensitivity analyses.
# These probabilities are not required in the base case analysis (since the probability in the preceding branch is zero), but are included here as they are required in sensitivity analyses.
** NHS Reference Costs provide upper and lower quartiles around mean estimates; the difference between the mean and the upper quartile was captured, and used as a crude estimate of standard error in the PSA. Where this was not possible, the standard error was assumed to be 20% of the mean cost.
†† A standard error of 0.09 was assumed, based on a previous study.[10]

CABG, Coronary Artery Bypass Graft; CMR, Cardiovascular Magnetic Resonance; ECHO, Echocardiography; FFR; Fractional flow reserve; MACE, Major adverse cardiac events; MI, Myocardial infarction; PCI, Percutaneous coronary intervention; PSA, Probabilistic sensitivity analysis; QALYs, Quality-adjusted life years; SE, Standard error

APPENDIX B – MODEL PARAMETERS, SOURCES AND ASSUMPTIONS FOR MODEL 2 (UNOBSTRUCTED CORONARY ARTERIES)

**Testing and clinical parameters**			
Branch of model	Base-case value (Positive/total)	Distribution for PSA	Source
For both arms			
Probability of truly having had an myocardial infarction (MI) (prevalence)	0.24 (429/1801)	Beta	Pasupathy 2015[11]
Standard ECHO and CMR			
Probability of ischaemia test positive and treatment for MI | MI (sensitivity of test)	1*	Beta	
Probability of MACE within 12 months | treatment for MI	0.21†	Beta	Kang 2011,[12] Pathik 2016[13]
Probability of MACE within 12 months | no treatment for MI	0.26†§	Beta	Kang 2011,[12] Pathik 2016,[13] Lindahl 2017[14]
Probability of ischaemia test positive and treatment for MI | no MI (1 minus specificity of test)	0*	Beta	
Probability of MACE within 12 months | treatment for MI	0.03†§	Beta	Kang 2011,[12] Pathik 2016[13]
Probability of MACE within 12 months | no treatment for MI	0.03†	Beta	Kang 2011,[12] Pathik 2016[13]
Standard ECHO			
Probability of ischaemia test positive and treatment for MI | MI (sensitivity of test)	0.47 (25/53)#	Beta	Dastidar 2017[15]
Probability of MACE within 12 months | treatment for MI	0.21†	Beta	Kang 2011,[12] Pathik 2016[13]
Probability of MACE within 12 months | no treatment for MI	0.26†	Beta	Kang 2011,[12] Pathik 2016,[13] Lindahl 2017[14]
Probability of ischaemia test positive and treatment for MI | no MI (1 minus specificity of test)	0.62 (93/151)#	Beta	Dastidar 2017[15]
Probability of MACE within 12 months | treatment for MI	0.03†	Beta	Kang 2011,[12] Pathik 2016[13]
Probability of MACE within 12 months | no treatment for MI	0.03†	Beta	Kang 2011,[12] Pathik 2016[13]

Cost parameters			
Resource	Unit cost (SE)**	Distribution for PSA	Source
Standard ECHO	£72 (22)	Gamma	NHS Reference Costs 2015/16.[4] Diagnostic Imaging. Outpatient. RD51A, Simple Echocardiogram, 19 years and over.
CMR	£264 (154)	Gamma	NHS Reference Costs 2015/16.[4] Diagnostic Imaging. Weighted average of all Cardiac Magnetic Resonance Imaging Scan codes.
Cardiac rehabilitation	£364 (73)	Gamma	Assume offered eight sessions, but only 50% update, so cost four sessions (one first and three follow up appointments, individual costs below)
First appointment	£97		NHS Reference Costs 2015/16.[4] Consultant Led. Cardiac Rehabilitation. Non-Admitted Face to Face Attendance, First.
Follow up appointment	£89		NHS Reference Costs 2015/16.[4] Consultant Led. Cardiac Rehabilitation. Non-Admitted Face to Face Attendance, Follow-Up.
Cardiac medications for treatment of MI (all once daily by mouth)			
Aspirin (75mg)	£0.04		BNF 73, March 2017[5]
Clopidogrel (75mg)	£0.05		BNF 73, March 2017[5]
Atorvastatin (80mg)	£0.07		BNF 73, March 2017[5]
Bisoprolol (2.5mg)	£0.02		BNF 73, March 2017[5]
Ramipril (2.5mg)	£0.04		BNF 73, March 2017[5]
Total daily cost	£0.22		
Total annual cost	£80 (16)	Gamma	
Treatment for non-MI cause (cardiac medications) – total annual cost	£40 (8)	Gamma	Assume half of these patients are taken off cardiac medications above (these medications are given for cardiomyopathy but not for myocarditis)
Outpatient follow up appointment (assumed to occur at 4-6 weeks post-discharge)	£122 (22)	Gamma	NHS Reference Costs 2015/16.[4] Consultant led appointments. Non-Admitted Face to Face Attendance, Follow-Up. Cardiology.
MACE	£2,808 (562)	Gamma	The proportion of each component of MACE was taken from Pathik,[13] and a weighted average of the costs of each component was calculated, based on the unit costs provided in Appendix A. Given the small number of MACE

			events, calculations were based on MI and non-MI patients combined.
Additional healthcare costs to one year	£2221 (444)	Gamma	Office of Health Economics estimate of the annual cost per person of NHS care inflated to 2015/16 prices using the hospital and community health services inflation index[6, 7] For patients who die, costs are assumed to be half of this (£1111).
Additional healthcare costs to one year for those with MACE	£1602 (320)	Gamma	MI and stroke were assumed to be associated with continuing care costs post discharge. Costs from Greenhalgh (2011),[8] were inflated to 2015/16 prices.[6] Separate costs were provided for disabling and non-disabling stroke; according to Davies (2006) 58% of strokes are disabling,[9] and this percentage was used to weight the two continuing care costs for stroke. Additional costs to one year associated with MACE were calculated by weighting estimates of the annual costs of MI and stroke by the number of patients with these complications across the number of patients with MACE, from Pathik (2017).[13]
**Quality-adjusted life years**			
Patient group	Mean (SE)†† QALYs to one year	Distribution for PSA	Source
MACE within 12 months	0.686 (0.09)	Beta	Calculations and sources described in the text
No MACE within 12 months	0.768 (0.09)	Beta	

* In the base case analyses, standard ECHO combined with CMR is assumed to have perfect sensitivity and specificity, but these parameters are incorporated into the model so that alternative assumptions can be tested in sensitivity analyses.
† A standard error of 0.10 was assumed for the PSA.
§ These probabilities are not required in the base case analysis (since the probability in the preceding branch is zero), but are included here as they are required in sensitivity analyses.
# For further information on how estimates of sensitivity and specificity for standard ECHO were obtained, please see the final paragraph under 'Model probabilities'.
** NHS Reference Costs provide upper and lower quartiles around mean estimates; the difference between the mean and the upper quartile was captured, and used as a crude estimate of standard error in the PSA. Where this was not possible, the standard error was assumed to be 20% of the mean cost.
†† A standard error of 0.09 was assumed, based on a previous study.[10]

CMR, Cardiovascular Magnetic Resonance; ECHO, Echocardiography; MACE, Major adverse cardiac events; MI, Myocardial infarction; PSA, Probabilistic sensitivity analysis; QALYs, Quality-adjusted life years

APPENDIX C – DETERMINISTIC ONE-WAY SENSITIVITY ANALYSES FOR MODEL 1 (MULTI-VESSEL DISEASE)

SA	Parameter varied	Base case	Alternative strategies for sensitivity analysis
1	Probabilities associated with decision on revascularisation from index angiogram:	Based on expert opinion:	Based on PIPA data:
	Probability index angiogram leads to revascularisation of second vessel	0.30 (30/100)	0.11 (78/717)
	Probability of uncertainty following index angiogram and need for ischaemia testing	0.60 (60/100)	0.15 (111/717)
	Probability index angiogram leads to no revascularisation of second vessel	0.10 (10/100)	0.74 (528/717)
2a	Sensitivity of CMR	1	0.8 (SE 0.1)
2b	Specificity of CMR	1	0.8 (SE 0.1)
2c	Sensitivity and specificity of CMR (labelled test accuracy in manuscript Table 1)	1 and 1	0.8 (SE 0.1) for each
3a	Sensitivity of FFR	1	0.8 (SE 0.1)
3b	Specificity of FFR	1	0.8 (SE 0.1)
3c	Sensitivity and specificity of FFR (labelled test accuracy in manuscript Table 1)	1 and 1	0.8 (SE 0.1) for each
4a	Sensitivity of stress ECHO	0.70	0.791 (SE 0.008) [54]
4b	Specificity of stress ECHO	0.77 (reported 1-specificity =0.23)	1-specificity 0.129 (SE 0.007) [54]
4c	Sensitivity and specificity of stress ECHO (labelled test accuracy in manuscript Table 1)	0.70 and 0.77 (reported 1-specificity =0.23)	Sensitivity: 0.791 (SE 0.008) and 1-specificity 0.129 (SE 0.007) [54]
5	Probability of MACE for truly ischaemic patients who had a second revascularisation, truly ischaemic patients who did not have a second revascularisation, and truly not ischaemic patients whether or not they had a second revascularisation	0.09, 0.31, 0.13	All ±0.05
6	Cost of CMR	£264	± 20%
7	Cost of angiogram and FFR	£1340	± 20%

8	QALYs for patients with MACE with and without revascularisation of a second vessel	0.684, 0.686	Base case -0.2 (0.484, 0.486)
9	QALYs for patients with no MACE with and without revascularisation of a second vessel	0.766, 0.768	Base case +0.2 (0.966, 0.968)

CMR, Cardiovascular Magnetic Resonance; ECHO, Echocardiography; FFR, Fractional flow reserve; MACE, Major adverse cardiac events; QALY, Quality-adjusted life years; SA, Sensitivity analysis; SE, Standard error

APPENDIX D – DETERMINISTIC ONE-WAY SENSITIVITY ANALYSES FOR MODEL 2 (UNOBSTRUCTED CORONARY ARTERIES)

SA	Parameter varied	Base case	Alternative strategies for sensitivity analysis
1a	Sensitivity of standard ECHO plus CMR	1	0.8 (SE 0.1)
1b	Specificity of standard ECHO plus CMR	1	0.8 (SE 0.1)
1c	Sensitivity and specificity of standard ECHO plus CMR (labelled test accuracy in manuscript Table 2)	1 and 1	0.8 (SE 0.1) for each
2a	Sensitivity of standard ECHO	0.47	0.7 (SE 0.1)
2b	Specificity of standard ECHO	0.38	0.7 (SE 0.1)
2c	Sensitivity and specificity of standard ECHO (labelled test accuracy in manuscript Table 2)	0.47 and 0.38	0.7 (SE 0.1) for each
3	Probability of MACE for patients who had a MI and treatment, for patients who had a MI and no treatment, and for patients without MI regardless of whether they had treatment (ratio of events in MI:non-MI group =4)*	0.21, 0.26, 0.03	0.18, 0.23, 0.05
4	Probability of MACE (ratio=2)	0.21, 0.26, 0.03	0.13, 0.17, 0.06
5	Probability of MACE (ratio=1)	0.21, 0.26, 0.03	0.08, 0.10, 0.08
6	Cost of CMR	£264	± 20%
7	Cost of additional healthcare costs to one year for patients with MACE - increase the proportion who die from 8% to 50%	£1602	£874
8	QALYs for patients with MACE	0.686	Base case -0.2 (0.486)
9	QALYs for patients with no MACE	0.768	Base case +0.2 (0.968)
10	QALYs for patients with MACE - increase the proportion who die from 8% to 50%	0.686	0.525

** For further information on this ratio, please see the Clinical parameters section of the manuscript.*
CMR, Cardiovascular Magnetic Resonance; ECHO, Echocardiography; MACE, Major adverse cardiac events; MI, Myocardial infarction; QALY, Quality-adjusted life years; SA, Sensitivity analysis; SE, Standard error

REFERENCES

1. Tonino PA, Fearon WF, De Bruyne B, et al. Angiographic versus functional severity of coronary artery stenoses in the FAME study fractional flow reserve versus angiography in multivessel evaluation. *J Am Coll Cardiol* 2010;55(25):2816-21.
2. Smits PC, Abdel-Wahab M, Neumann FJ, et al. Fractional Flow Reserve-Guided Multivessel Angioplasty in Myocardial Infarction. *N Engl J Med* 2017;376(13):1234-44.
3. Gurunathan S, Young G, Parsons G, et al. 132 Diagnostic Accuracy of Stress Echocardiography Compared with Invasive Coronary Angiography with Fractional Flow Reserve for The Diagnosis of Haemodynamically Significant Cad in Patients with Known or Suspected CAD. *Heart* 2016;102(Suppl 6):A94-A95.
4. Department of Health. National Schedule of Reference Costs 2015-16. London: Department of Health 2016.
5. Joint Formulary Committee. British National Formulary No. 73. London: British Medical Association and Royal Pharmaceutical Society of Great Britian 2017.
6. Curtis L, Burns A. Unit costs of health and social care 2016. Canterbury: Personal Social Services Research Unit, University of Kent 2016.
7. Hawe E, Cockcroft L. OHE guide to UK health and health care statistics. London: Office of Health Economics 2013.
8. Greenhalgh J, Bagust A, Boland A, et al. Clopidogrel and modified-release dipyridamole for the prevention of occlusive vascular events (review of Technology Appraisal No. 90): a systematic review and economic analysis. *Health Technol Assess* 2011;15(31):1-178.
9. Davies L, Brown TJ, Haynes S, et al. Cost-effectiveness of cell salvage and alternative methods of minimising perioperative allogeneic blood transfusion: a systematic review and economic model. *Health Technol Assess* 2006;10(44).
10. Palmer S, Sculpher M, Philips Z, et al. A cost-effectiveness model comparing alternative management strategies for the use of glycoprotein IIb/IIIa antagonists in non-st-elevation acute coronary syndrome. Report to the National Institute for Clinical Excellence 2002.
11. Pasupathy S, Air T, Dreyer RP, et al. Systematic review of patients presenting with suspected myocardial infarction and nonobstructive coronary arteries. *Circulation* 2015;131(10):861-70.
12. Kang WY, Jeong MH, Ahn YK, et al. Are patients with angiographically near-normal coronary arteries who present as acute myocardial infarction actually safe? *Int J Cardiol* 2011;146(2):207-12.
13. Pathik B, Raman B, Mohd Amin NH, et al. Troponin-positive chest pain with unobstructed coronary arteries: incremental diagnostic value of cardiovascular magnetic resonance imaging. *Eur Heart J Cardiovasc Imaging* 2016;17(10):1146-52.
14. Lindahl B, Baron T, Erlinge D, et al. Medical Therapy for Secondary Prevention and Long-Term Outcome in Patients With Myocardial Infarction With Nonobstructive Coronary Artery Disease. *Circulation* 2017;135(16):1481-89.
15. Dastidar AG, Rodrigues JCL, Johnson TW, et al. Myocardial Infarction With Nonobstructed Coronary Arteries: Impact of CMR Early After Presentation. *JACC Cardiovasc Imaging* 2017;10(10 Pt A):1204-06.
