# Supplementary material for: Cardiovascular magnetic resonance in emergency patients with multivessel disease or unobstructed coronary arteries: a cost-effectiveness analysis in the UK
Source: BMJ Open. 2019 Jul 11;9(7):e025700. doi: 10.1136/bmjopen-2018-025700 (PMC6629389; doi:10.1136/bmjopen-2018-025700)
Supplement: Author's manuscript [file bmjopen-2018-025700.draft_revisions.pdf]

BMJ Open

BMJ Open is committed to open peer review. As part of this commitment we make the peer review history of every article we publish publicly available.

When an article is published we post the peer reviewers' comments and the authors' responses online. We also post the versions of the paper that were used during peer review. These are the versions that the peer review comments apply to.

The versions of the paper that follow are the versions that were submitted during the peer review process. They are not the versions of record or the final published versions. They should not be cited or distributed as the published version of this manuscript.

BMJ Open is an open access journal and the full, final, typeset and author-corrected version of record of the manuscript is available on our site with no access controls, subscription charges or pay-per-view fees (http://bmjopen.bmj.com).

If you have any questions on BMJ Open's open peer review process please email info.bmjopen@bmj.com

Cardiovascular magnetic resonance in emergency patients with multi-vessel disease or unobstructed coronary arteries: a cost-effectiveness analysis in the United Kingdom

Journal:	*BMJ Open*
Manuscript ID	bmjopen-2018-025700
Article Type:	Research
Date Submitted by the Author:	27-Jul-2018

Complete List of Authors:	Stokes, Elizabeth; University of Oxford, Health Economics Research Centre, Nuffield Department of Population Health<br>Doble, Brett; University of Oxford, Nuffield Department of Population Health<br>Pufulete, Maria; University of Bristol, Clinical Trials and Evaluation Unit; University Hospitals Bristol NHS Foundation Trust and University of Bristol, NIHR Bristol Biomedical Research Centre<br>Reeves, Barnaby; University of Bristol, Clinical Trials and Evaluation Unit; University Hospitals Bristol NHS Foundation Trust and University of Bristol, NIHR Bristol Biomedical Research Centre<br>Bucciarelli Ducci, Chiara; University Hospitals Bristol NHS Foundation Trust and University of Bristol, NIHR Bristol Biomedical Research Centre; University Hospitals Bristol NHS Foundation Trust, Bristol Heart Institute<br>Dorman, Stephen; University Hospitals Bristol NHS Foundation Trust, Bristol Heart Institute<br>Greenwood, John; University of Leeds, Leeds Institute of Cardiovascular and Metabolic Medicine<br>Anderson, Richard; University Hospital of Wales, Cardiology<br>Wordsworth, Sarah; University of Oxford, Health Economics Research Centre, Nuffield Department of Population Health

Keywords:	Cardiovascular disease, Cardiovascular magnetic resonance, Cost-utility analysis, Diagnostic accuracy, Economic evaluation, Myocardial infarction < CARDIOLOGY

SCHOLARONE™ Manuscripts

Cardiovascular magnetic resonance in emergency patients with multi-vessel disease or unobstructed coronary arteries: a cost-effectiveness analysis in the United Kingdom

Elizabeth A. Stokes[1*], Brett Doble[1], Maria Pufulete[2,3], Barnaby C. Reeves[2,3], Chiara Bucciarelli-Ducci[3,4], Stephen Dorman[4], John P Greenwood[5], Richard A Anderson[6], Sarah Wordsworth[1]

Affiliations

[1]Health Economics Research Centre, Nuffield Department of Population Health, University of Oxford, Oxford, United Kingdom

[2]Clinical Trials and Evaluation Unit, University of Bristol, Bristol, United Kingdom

[3]NIHR Bristol Biomedical Research Centre, University Hospitals Bristol NHS Foundation Trust and University of Bristol, Bristol, United Kingdom

[4]Bristol Heart Institute, University Hospitals Bristol NHS Foundation Trust, Bristol, United Kingdom

[5]Leeds Institute of Cardiovascular and Metabolic Medicine, University of Leeds, Leeds, United Kingdom

[6]University Hospitals of Wales, Heath Park, Cardiff, United Kingdom

*Corresponding author: Elizabeth Stokes, Health Economics Research Centre, Nuffield Department of Population Health, University of Oxford, Old Road Campus, Headington, Oxford, OX3 7LF, United Kingdom. Tel: +44(0)1865 289276; Email: elizabeth.stokes@dph.ox.ac.uk

Word Count: 4032

For peer review only

ABSTRACT

Objective: To identify the key drivers of cost-effectiveness for cardiovascular magnetic resonance (CMR) when patients activate the primary percutaneous coronary intervention (PPCI) pathway.

Design: Economic decision models for two patient subgroups populated from secondary sources, each with a one-year time horizon from the perspective of the National Health Service (NHS) and personal social services in the UK.

Setting: Usual care (with or without CMR) in the NHS.

Participants: Patients who activated the PPCI pathway, and for Model 1: underwent an emergency coronary angiogram and PPCI, and were found to have multi-vessel coronary artery disease. For Model 2: underwent an emergency coronary angiogram, and were found to have unobstructed coronary arteries.

Interventions: Model 1 (multi-vessel disease) compared two different ischaemia testing methods, CMR or fractional flow reserve (FFR), versus stress echocardiography. Model 2 (unobstructed arteries) compared CMR with standard echocardiography versus standard echocardiography alone.

Main outcome measures: Key drivers of cost-effectiveness for CMR, incremental costs and quality-adjusted life years (QALYs) and incremental cost-effectiveness ratios.

Results: In both models the incremental costs and QALYs between CMR (or FFR, model 1) versus no CMR (stress echocardiography, model 1 and standard echocardiography, model 2) were small (CMR: -£64 (95% CI -£232, £187) / FFR: £360 (95% CI -£116, £844) and CMR/FFR: 0.0012 QALYs, (95% CI -0.0076, 0.0093)) and (£98, (95% CI -£199, £488) and 0.0005 QALYs (95% CI -0.0050, 0.0077)) respectively. The diagnostic accuracy of the tests was the key driver of cost-effectiveness for both patient groups.

Conclusions: If CMR were introduced for all subgroups of patients who activate the PPCI pathway, it is likely that diagnostic accuracy would be a key determinant of its cost-effectiveness. Further research is needed to definitively answer whether revascularisation guided by CMR or FFR leads to different clinical outcomes in acute coronary syndrome patients with multi-vessel disease.

Keywords: cardiovascular disease, cardiovascular magnetic resonance (CMR), cost-utility analysis, diagnostic accuracy, economic evaluation, myocardial infarction

For peer review only - http://bmjopen.bmj.com/site/about/guidelines.xhtml

ARTICLE SUMMARY

Strengths and limitations of this study

- We present the first cost-effectiveness analysis of cardiovascular magnetic resonance (CMR) and fractional flow reserve (FFR) ischaemia tests compared to stress echocardiography in patients who activate the primary percutaneous coronary intervention (PPCI) pathway and are found to have multi-vessel coronary disease, and the first cost-effectiveness analysis of introducing CMR in patients who activate the PPCI pathway and are found to have unobstructed coronary arteries.

- The results of our analyses provide much needed evidence to guide detailed measurement of key drivers of cost-effectiveness for future research.

- The majority of model parameter estimates were based on single studies with small sample sizes, conducted outside the UK where patient pathways differ.

- CMR and FFR tests were treated as reference standards (assumed 100% sensitivity and specificity) in base-case analyses; sensitivity analyses show that relative cost-effectiveness varies substantially depending on assumptions about these parameters.

For peer review only

INTRODUCTION

Patients with acute coronary syndrome (ACS) and suspected acute thrombotic coronary occlusion activate the primary percutaneous coronary intervention (PPCI) pathway. In the UK National Health Service (NHS), PPCI is the main clinical approach to restore blood flow in the infarcted artery after ST-elevation myocardial infarction (STEMI).[1] Many patients presenting to hospital with STEMI have multi-vessel disease (40-65%),[2-7] which may require secondary revascularisation of the patients' non-infarcted territories. In addition, 3-16% of patients who activate the PPCI pathway do not receive PPCI because angiography sometimes shows that the coronary arteries are unobstructed.[8, 9] These factors result in considerable uncertainty and sometimes a lack of a definitive diagnosis of myocardial infarction (MI) in these patients, which can lead to inappropriate clinical management,[10, 11] which may in turn be associated with poor prognosis or unnecessary resource use.[12]

Cardiovascular magnetic resonance (CMR) is a non-invasive imaging technique that assesses heart structure with high temporal resolution, and can help inform management decisions for ACS patients who activate the PPCI pathway. CMR can facilitate differential diagnosis in the context of a normal coronary angiogram,[13-15] providing a definitive diagnosis in 65-90% of patients.[13, 16, 17] The benefits of CMR in other subgroups of PPCI patients, such as those found to have multi-vessel disease, are less clear. Long-term outcome data and studies reporting the cost-effectiveness of CMR are absent, further complicating resource allocation of CMR services within the NHS. Despite a lack of evidence, CMR has been increasingly used in ACS patients in the UK,[18] and is included in the 2017 European Society of Cardiology guidelines on the management of patients presenting with STEMI.[19]

There has also been a rapid increase in the use of fractional flow reserve (FFR) testing; a guide wire-based procedure which is undertaken through a standard guide catheter during invasive angiography to assess the degree of ischaemia in stenosed coronary arteries. Stress CMR is considered to have excellent diagnostic accuracy compared to FFR for detecting ischaemia.[20, 21] The main benefit of ischaemia testing using FFR is that lesions can be assessed and revascularised in the same session; however if revascularisation is not required, then the benefit of FFR over CMR is less clear. In addition to CMR and FFR, stress echocardiography (ECHO) and myocardial perfusion scintigraphy using single photon emission computed tomography (MPS-SPECT) are often used to evaluate patients with multi-vessel disease for residual ischaemia of bystander disease after PPCI for STEMI; stress ECHO is one of the most commonly used imaging modalities in the UK.[22]

For peer review only - http://bmjopen.bmj.com/site/about/guidelines.xhtml

In this paper, we present a health economic analysis based on a study that was designed to establish the feasibility of setting up a UK multi-centre registry to document CMR use in patients who activate the PPCI pathway and determine its prognostic value and impact on patient management.[23] Here we present the results of two economic decision models which we designed to compare the cost-effectiveness of introducing CMR in two patient subgroups: patients with multi-vessel disease and those with unobstructed coronary arteries. The aim was to identify how the results of the analyses change in response to sensitivity analyses to determine key drivers of cost-effectiveness that could be considered for detailed measurement in any future research.

METHODS

Overview and treatment strategies

We built two separate cost-effectiveness models in consultation with clinical experts to estimate the direct medical costs and quality-adjusted life years (QALYs) of introducing CMR after PPCI pathway activation in two patient subgroups over a one-year time horizon:

1) Model 1 compares two types of ischaemia testing: CMR or FFR to stress ECHO in patients with multi-vessel disease (commonly defined as stenosis >50%) after index angiogram and PPCI;
2) Model 2 compares CMR in addition to standard ECHO to standard ECHO alone in patients with unobstructed coronary arteries after index angiogram.

From these two models, it may be possible to make inferences about the likely drivers of cost-effectiveness of introducing CMR for all subgroups of patients who activate the PPCI pathway. The analysis was conducted from an NHS and personal social services perspective in the UK.[24]

Model structure

Model 1: multi-vessel disease

Model 1 uses a decision tree structure (Figure 1) to compare the cost-effectiveness of two types of ischaemia testing (CMR and FFR) to stress ECHO. All three tests are used to guide decisions regarding secondary revascularisation in patients with multi-vessel disease. Based on the findings of their emergency index angiogram at which the culprit lesion is treated, patients can enter one of three test/treatment pathways to determine whether further

For peer review only - http://bmjopen.bmj.com/site/about/guidelines.xhtml

For peer review only

treatment is required for non-culprit lesions at a later date: no ischaemia testing and secondary revascularisation; ischaemia testing to guide decisions for secondary revascularisation; or no ischaemia testing and no secondary revascularisation. Patients are then divided into whether they 'truly' have ischaemia or not. For patients receiving ischaemia testing, the test results can be either positive or negative (ischaemia present or not), and the model reflects potential misclassification of patients (i.e., ischaemia testing has a sensitivity and specificity). Patients entering any of the three test/treatment pathways are then at risk of experiencing a major adverse cardiovascular event (MACE) or no MACE over the one-year time horizon. MACE is a composite endpoint of all-cause mortality, MI, stroke or (repeat) revascularisation.

The model structure is the same for the three ischaemia testing options, but there are differences in the timing of testing and secondary revascularisation between these arms (note for all arms this testing and secondary revascularisation, if required, is at a later date than the index angiogram at PPCI and treatment of the culprit lesion). For CMR and stress ECHO, this is a two-step process (i.e., a patient has the ischaemia test, and then separately has secondary revascularisation). For FFR, this happens at the same time (i.e., a patient has their diagnostic angiogram and FFR, and if required, secondary revascularisation). We have assumed that this timing difference only affects costs, but not the model structure.

Model 2: unobstructed coronary arteries

Model 2 also uses a decision tree structure (Figure 2) to compare the cost-effectiveness of CMR with standard ECHO to 'current practice' of standard ECHO only. These tests are used to guide decisions in offering treatment for MI in patients who activate the PPCI pathway but are found to have unobstructed coronary arteries. In each arm, patients are divided according to whether they truly had an MI or not. Results for both testing approaches can be either positive or negative (MI occurred or not), with the model again reflecting the potential for misclassification. Patients receiving a positive test result are assumed to have had an MI and receive treatment (cardiac rehabilitation and medications). A negative test results in treatment for other cardiac causes of chest pain (fewer cardiac medications or none at all). Thereafter, patients are divided into whether they had MACE or not over the one-year time horizon.

For peer review only - http://bmjopen.bmj.com/site/about/guidelines.xhtml

Economic analyses

TreeAge Pro 2013 (TreeAge Software Inc., Williamstown, MA) was used to develop and analyse the models. Resources were valued in 2015/16 pounds sterling. The expected costs and QALYs associated with each testing strategy to one-year were calculated. Costs and QALYs were not discounted, as the time horizon was only one-year. Incremental cost-effectiveness ratios (ICERs) were calculated as the ratio of the difference in cost to the difference in QALYs between each testing option and its comparator.

Identification of model parameters

Testing and clinical parameters used in both models were derived from reviews of the literature. Unit costs were obtained from standard UK sources, notably the National Schedule of Reference Costs and the British National Formulary.[25, 26] Health state utility values were identifed by searching the Tufts Cost-effectiveness Analysis (CEA) Registry.[27]

For Model 1, we searched Medline using Medical Subject Headings and keywords for each ischaemia test (e.g. exp/pressure-wire or pressure-wire.mp or fractional flow reserve.mp and exp/myocardial infarction). Review articles, reference lists and citations of relevant studies were also evaluated. For Model 2, initial Medline searching identified a 2016 review paper,[28] which referenced the inaugural study of CMR in patients with unobstructed coronary arteries.[13] All citations of this primary paper were reviewed.[13] Two meta-analyses were identified and their citations were reviewed.[29, 30] Medline was also searched using keywords such as 'myocardial infarction with non-obstructive coronary arteries' and synonyms.

Complete lists of model parameters, their respective sources and assumptions made in their estimation are provided in Appendix A and B and briefly summarised below.

Testing parameters

Model 1: multi-vessel disease

CMR and FFR were treated as reference standards with assumed 100% sensitivity and specificity. While FFR is the reference standard for detecting ischaemia in patients with stable coronary artery disease, there is uncertainty in how FFR-guided revascularisation influences outcome in this high-risk group of STEMI patients with multi-vessel disease. It was therefore considered a reasonable base-case assumption that the sensitivities and specificities of CMR and FFR were the same in this population. While in practice their sensitivity and specificity are likely to be a little less than 100%, there is no gold standard test

For peer review only - http://bmjopen.bmj.com/site/about/guidelines.xhtml

for ischaemia in STEMI patients with multi-vessel disease with which to compare; lower sensitivities and specificities were explored in sensitivity analyses. The assumption of 100% sensitivity and specificity for CMR and FFR results in identical probabilities and outcomes, but differences in costs for the two arms. Sensitivity and specificity values for stress ECHO were obtained from Gurunathan et al.[31]

Model 2: unobstructed coronary arteries

CMR with standard ECHO was treated as a reference standard, whereas sensitivity and specificity values for standard ECHO were obtained from Dastidar et al.[32]

Clinical parameters

Model 1: multi-vessel disease

The probabilities of patients entering one of the three test/treatment pathways were based on expert clinical opinion. Probabilities of true ischaemia for each test/treatment pathway were derived from the FAME study.[33] Subgroup outcomes from Smits et al. were used to estimate probabilities of MACE for three patient groups: truly ischaemic patients who had secondary revascularisation, truly ischaemic patients who did not have secondary revascularisation, and truly non-ischaemic patients, regardless of secondary revascularisation.[34]

Model 2: unobstructed coronary arteries

Studies reporting the probability of MACE in patients with unobstructed coronary arteries have used varying definitions of MACE and follow-up periods (12-34 months).[35-38] In patients with <50% coronary artery stenosis, Kang et al. reported a 7.8% risk of MACE at one-year,[37] but their MACE definition did not include stroke. Three studies reported MACE, broken down by CMR diagnosis.[35, 36, 38] Pathik reported MACE for 27% of patients diagnosed with MI, and for 5% of patients without MI (diagnosed with myocarditis, cardiomyopathy or normal CMR) over a median of 24 months.[38] In the other two studies, no MACE outcomes were observed in the non-MI patients. The MACE estimates reported in Pathik et al. were converted to continuous rates (assuming events occur evenly over 24 months of follow-up). The ratio of events in the MI patients compared to the non-MI patients was six, and was used with the overall estimate of MACE at one-year from Kang et al. (0.078),[37] and the probability of patients truly having an MI from Pasupathy et al. (0.24),[29] to calculate the probability of MACE in patients who truly had an MI (0.21) and in those who did not have an MI (0.03).

For peer review only - http://bmjopen.bmj.com/site/about/guidelines.xhtml

The hazard ratio for MACE in patients who were taking statins compared to those who were not (0.77) reported by Lindahl et al. was used to adjust the probability of MACE for patients who had an MI, giving a probability of MACE for those with MI but without treatment of 0.26.[39] For patients without MI, the probability of MACE of 0.03 was assumed to apply regardless of treatment.

Cost parameters

Model 1: multi-vessel disease

The costs considered for all three ischaemia testing strategies included: the cost of ischaemia testing (CMR, FFR, or stress ECHO) if required, secondary revascularisation, adverse events included in MACE (initial inpatient and post-discharge costs), medications and cardiac rehabilitation offered to all patients and additional healthcare costs beyond hospital discharge to one-year, including follow-up outpatient appointments.

Model 2: unobstructed coronary arteries

The costs considered for the two testing strategies included: the cost of testing (CMR and/or standard ECHO), cardiac rehabilitation and medications for patients classified as either having had an MI or a non-MI diagnosis (details in Appendix B), adverse events included in MACE (initial inpatient and post-discharge costs) and additional healthcare costs beyond hospital discharge to one-year, including a follow-up appointment.

Quality-adjusted life years

Model 1: multi-vessel disease

QALYs were estimated over one-year as a function of survival and health state utility values. Since death is one of the components of MACE, we assumed that patients without MACE are all alive at one-year and that the probability of dying for MACE patients was 0.08 as estimated by Smits et al.[34] We identified mean utility values of 0.72, 0.77 and 0.77 at baseline (during index hospitalisation), one and twelve-months respectively estimated using the UK EQ-5D-3L tariff in patients with STEMI having PPCI.[27, 40]

QALYs were calculated assuming a patient's utility changed linearly between each time point or death. Patients who died were assumed to die midway through the time-period (six months), and have a utility of zero after death. QALYs for patients without MACE who were all alive at one-year were calculated based on the utility values for the three time-points above.[40] QALYs for patients with MACE were also calculated using these three utility values, but assuming 8% of patients died to produce a weighted average for patients alive and

For peer review only - http://bmjopen.bmj.com/site/about/guidelines.xhtml

dead at one-year.[34] A utility decrement of 0.05 was applied to this weighted average to reflect the reduced quality-of-life of patients with MACE compared to those without.[41] QALYs for patients who had secondary revascularisation were modified to assume their utility at the one-month time-point was repeated for a subsequent month.

Model 2: unobstructed coronary arteries

Searches of the CEA Registry did not yield any utility values for patients with unobstructed coronary arteries, and therefore QALYs for patients with and without MACE in Model 2 were calculated using the same utility values and methods as for Model 1.[40]

Base-case and sensitivity analyses

The best available point estimates were used in the base-case analyses in which ICERs were the summary measures. Since this was a feasibility study, the actual ICERs are not the main interest, but rather how they change in response to sensitivity analyses.

One-way deterministic sensitivity analyses (DSAs) were conducted to test the impact of varying the base-case values for several model parameters. Alternative values for DSAs (Appendix C and D) were obtained from substitute evidence sources, clinical judgment (e.g. 8% probability of death for patients with MACE was thought to be low, and a relative risk of 6.0 to be high for events in MI compared to non-MI patients, so higher and lower values were explored) or altering mean values for unit costs by ±20%. Threshold analyses were also conducted to establish the cost of each test at which the expected costs associated with an alternative strategy would be identical.

Probabilistic sensitivity analyses (PSAs) were used to account for the impact on results of all uncertain parameters simultaneously. Parameters were assigned distributions based on the precision of estimates available to describe a range of plausible values, and 1,000 randomly selected values from each distribution were generated and results calculated for each run of the model. 95% confidence intervals and scatter plots of the 1,000 incremental costs and QALYs for each comparison were then assessed.

Patient and public involvement

Patients and the public were not involved in the design of this study.

For peer review only - http://bmjopen.bmj.com/site/about/guidelines.xhtml

RESULTS

Base-case results

Model 1: multi-vessel disease

Over one-year, the expected total costs of stress ECHO, CMR and FFR strategies per patient were £5495, £5431 and £5855 respectively (Table 1). Use of stress ECHO produced 0.7564 QALYs, whereas CMR and FFR both resulted in an additional 0.0012 QALYs gained. CMR dominated stress ECHO as a CMR strategy was less costly (-£64) and more effective (+0.0012 QALYs). FFR was more costly (£360) and more effective (+0.0012 QALYs) than stress ECHO, resulting in an ICER above the accepted cost-effectiveness threshold of £20,000-£30,000 per QALY.

For peer review only

For peer review only - http://bmjopen.bmj.com/site/about/guidelines.xhtml

Table 1. Base-case and deterministic one-way sensitivity analyses results for Model 1 (multi-vessel disease)

DSA	Ischaemia testing option	Costs (£) Mean (SE)	QALYs Mean (SE)	Difference in costs to Stress ECHO Mean (95% CI)	Difference in QALYs to Stress ECHO Mean (95% CI)	ICER (£) – compared to Stress ECHO
Base	Stress ECHO	5495 (556)	0.7564 (0.0545)			
Case	CMR	5431 (560)	0.7576 (0.0551)	-64 (-232, 187)	0.0012 (-0.0076, 0.0093)	CMR dominant (-53,563)
	FFR	5855 (539)	0.7576 (0.0551)	360 (-116, 844)	0.0012 (-0.0076, 0.0093)	300,216
1 Decision	Stress ECHO	4575 (449)	0.7537 (0.0654)			
From	CMR	4558 (500)	0.7540 (0.0661)	-17 (-62, 45)	0.0003 (-0.0022, 0.0026)	CMR dominant (-53,563)
angiogram	FFR	4667 (500)	0.7540 (0.0661)	93 (-21, 214)	0.0003 (-0.0022, 0.0026)	300,216
2 CMR test	Stress ECHO	5495 (556)	0.7564 (0.0545)			
Accuracy	CMR	5558 (600)	0.7567 (0.0572)	63 (-202, 390)	0.0004 (-0.0119, 0.0108)	168,453
3 FFR test	Stress ECHO	5495 (556)	0.7564 (0.0545)			
Accuracy	FFR	5944 (568)	0.7567 (0.0572)	449 (-18, 947)	0.0004 (-0.0119, 0.0108)	1,207,875
4 ECHO	Stress ECHO	5431 (553)	0.7568 (0.0555)			
Test	CMR	5431 (560)	0.7576 (0.0551)	+0 (-128, 253)	0.0008 (-0.0022, 0.0036)	201
Accuracy	FFR	5855 (539)	0.7576 (0.0551)	424 (18, 888)	0.0008 (-0.0022, 0.0036)	529,193
5	Stress ECHO	5674 (586)	0.7520 (0.0566)			
MACE	CMR	5610 (580)	0.7533 (0.0574)	-65 (-233, 164)	0.0012 (-0.0078, 0.0087)	CMR dominant (-53,434)
+0.05	FFR	6033 (575)	0.7533 (0.0574)	359 (-85, 831)	0.0012 (-0.0078, 0.0087)	296,587
5	Stress ECHO	5317 (564)	0.7607 (0.0589)			
MACE	CMR	5252 (565)	0.7619 (0.0595)	-65 (-244, 192)	0.0012 (-0.0081, 0.0097)	CMR dominant (-53,463)
-0.05	FFR	5676 (533)	0.7619 (0.0595)	359 (-112, 830)	0.0012 (-0.0081, 0.0097)	297,408

For peer review only - http://bmjopen.bmj.com/site/about/guidelines.xhtml

DSA	Ischaemia testing option	Costs (£) Mean (SE)	QALYs Mean (SE)	Difference in costs to Stress ECHO Mean (95% CI)	Difference in QALYs to Stress ECHO Mean (95% CI)	ICER (£) – compared to Stress ECHO
6 CMR cost	Stress ECHO	5495 (556)	0.7564 (0.0545)			
+20%	CMR	5463 (556)	0.7576 (0.0551)	-32 (-218, 187)	0.0012 (-0.0076, 0.0093)	CMR dominant (-27,021)
6 CMR cost	Stress ECHO	5495 (556)	0.7564 (0.0545)			
-20%	CMR	5399 (551)	0.7576 (0.0551)	-96 (-268, 159)	0.0012 (-0.0076, 0.0093)	CMR dominant (-80,104)
7 FFR cost	Stress ECHO	5495 (556)	0.7564 (0.0545)			
+20%	FFR	6015 (560)	0.7576 (0.0551)	521 (78, 992)	0.0012 (-0.0076, 0.0093)	434,425
7 FFR cost	Stress ECHO	5495 (556)	0.7564 (0.0545)			
-20%	FFR	5694 (537)	0.7576 (0.0551)	199 (-240, 622)	0.0012 (-0.0076, 0.0093)	166,007
8 QALYs	Stress ECHO	5495 (556)	0.7306 (0.0559)			
for MACE	CMR	5431 (560)	0.7346 (0.0565)	-64 (-232, 187)	0.0040 (-0.0057, 0.0117)	CMR dominant (-16,041)
-0.2	FFR	5855 (539)	0.7346 (0.0565)	360 (-116, 844)	0.0040 (-0.0057, 0.0117)	89,907
9 QALYs	Stress ECHO	5495 (556)	0.9306 (0.0576)			
for no	CMR	5431 (560)	0.9346 (0.0587)	-64 (-232, 187)	0.0040 (-0.0071, 0.0104)	CMR dominant (-16,041)
MACE +0.2	FFR	5855 (539)	0.9346 (0.0587)	360 (-116, 844)	0.0040 (-0.0071, 0.0104)	89,907

CMR, Cardiovascular Magnetic Resonance; CI, Confidence interval; ECHO, Echocardiography; FFR, Fractional flow reserve; ICER, Incremental cost-effectiveness ratio; MACE, Major adverse cardiovascular events; QALY, Quality-adjusted life year; SA, Sensitivity analysis; SE, Standard error

Note: all costs and ICERs are rounded to nearest pound

For peer review only - http://bmjopen.bmj.com/site/about/guidelines.xhtml

Model 2: unobstructed coronary arteries

Over one-year, the expected total costs of CMR with standard ECHO and standard ECHO alone strategies per patient were £3130 and £3032 respectively (Table 2). Use of CMR with standard ECHO produced 0.0005 more QALYs than standard ECHO alone, at an increase in costs of £98, resulting in an ICER above the accepted cost-effectiveness threshold.

For peer review only

For peer review only - http://bmjopen.bmj.com/site/about/guidelines.xhtml

Table 2. Base-case and deterministic one-way sensitivity analyses results for Model 2 (unobstructed coronary arteries)

DSA	Testing option	Costs (£) Mean (SE)	QALYs Mean (SE)	Difference in costs Mean (95% CI)	Difference in QALYs Mean (95% CI)	ICER (£)
Base	Standard ECHO	3032 (564)	0.7615 (0.0837)			
Case	Standard ECHO and CMR	3130 (589)	0.7620 (0.0844)	98 (-199, 488)	0.0005 (-0.0050, 0.0077)	190,114
1 CMR test	Standard ECHO	3032 (564)	0.7615 (0.0837)			
Accuracy	Standard ECHO and CMR	3183 (591)	0.7618 (0.0850)	151 (-105, 523)	0.0003 (-0.0026, 0.0053)	469,998
2 ECHO test	Standard ECHO	3068 (585)	0.7617 (0.0832)			
Accuracy	Standard ECHO and CMR	3130 (589)	0.7620 (0.0844)	62 (-209, 453)	0.0003 (-0.0032, 0.0048)	211,617
3 MACE	Standard ECHO	3067 (541)	0.7608 (0.0826)			
(ratio=4)	Standard ECHO and CMR	3165 (559)	0.7614 (0.0830)	98 (-192, 465)	0.0005 (-0.0043, 0.0069)	190,154
4 MACE	Standard ECHO	3043 (573)	0.7613 (0.0829)			
(ratio=2)	Standard ECHO and CMR	3146 (606)	0.7617 (0.0834)	104 (-162, 498)	0.0004 (-0.0051, 0.0063)	250,905
5 MACE	Standard ECHO	3046 (579)	0.7612 (0.0847)			
(ratio=1)	Standard ECHO and CMR	3161 (596)	0.7614 (0.0850)	115 (-168, 492)	0.0002 (-0.0051, 0.0069)	554,609
6 CMR cost	Standard ECHO	3032 (564)	0.7615 (0.0837)			
+20%	Standard ECHO and CMR	3183 (579)	0.7620 (0.0844)	151 (-133, 532)	0.0005 (-0.0050, 0.0077)	292,836
6 CMR cost	Standard ECHO	3032 (564)	0.7615 (0.0837)			
-20%	Standard ECHO and CMR	3077 (572)	0.7620 (0.0844)	45 (-229, 476)	0.0005 (-0.0050, 0.0077)	87,391
7 Reduce	Standard ECHO	2974 (548)	0.7615 (0.0837)			
MACE costs	Standard ECHO and CMR	3077 (574)	0.7620 (0.0844)	103 (-159, 502)	0.0005 (-0.0050, 0.0077)	198,992
8 QALYs for	Standard ECHO	3032 (564)	0.7457 (0.0879)			
MACE -0.2	Standard ECHO and CMR	3130 (589)	0.7474 (0.0886)	98 (-199, 488)	0.0018 (-0.0091, 0.0139)	55,281

For peer review only - http://bmjopen.bmj.com/site/about/guidelines.xhtml

DSA	Testing option	Costs (£) Mean (SE)	QALYs Mean (SE)	Difference in costs Mean (95% CI)	Difference in QALYs Mean (95% CI)	ICER (£)
9 QALYs for no MACE +0.2	Standard ECHO	3032 (564)	0.9457 (0.0905)			
	Standard ECHO and CMR	3130 (589)	0.9474 (0.0911)	98 (-199, 488)	0.0018 (-0.0094, 0.0134)	55,281
10 QALYs for MACE (50% die)	Standard ECHO	2994 (516)	0.7488 (0.0859)			
	Standard ECHO and CMR	3095 (542)	0.7503 (0.0864)	101 (-180, 464)	0.0015 (-0.0090, 0.0133)	66,040

CMR, Cardiovascular Magnetic Resonance; CI, Confidence interval; ECHO, Echocardiography; ICER, Incremental cost-effectiveness ratio; MACE, Major adverse cardiovascular events; QALY, Quality-adjusted life year; SA, Sensitivity analysis; SE, Standard error

Note: all costs and ICERs are rounded to nearest pound.

For peer review only

For peer review only - http://bmjopen.bmj.com/site/about/guidelines.xhtml

Sensitivity analyses

Model 1: multi-vessel disease

Table 1 reports the results of the DSAs for Model 1. DSA 1, varying the probabilities of a clinician ordering an ischaemia test and of a decision to perform a secondary revascularisation (or not), affects the mean costs and QALYs under each strategy but does not alter the cost-effectiveness results. When the sensitivity and specificity of CMR were reduced to 80% (DSA 2), CMR became more costly than stress ECHO, rather than dominant, and at accepted cost-effectiveness thresholds, would not be considered cost-effective relative to stress ECHO. When the sensitivity and specificity of FFR were reduced to 80% (DSA 3), conclusions did not alter, but the ICER increased. In DSA 4, using higher sensitivity and specificity values for stress ECHO, the ICER for CMR would still be considered cost-effective but was no longer dominant over stress ECHO, and FFR had an even more unfavourable ICER compared to stress ECHO. Altering the probability of MACE (DSA 5), the costs of the ischaemia tests (DSAs 6 and 7), and QALYs for patients with and without MACE (DSAs 8 and 9), did not alter cost-effectiveness conclusions.

Threshold analyses identified that if the cost of CMR increased to £371 (from a base-case value of £264), there would be no difference in the expected costs of the CMR and stress ECHO strategies. Similarly, the cost of angiogram and FFR would need to be reduced to £250 and £415 (from a base-case value of £1,340) for there to be no difference in the expected costs of CMR and stress ECHO strategies respectively compared to FFR.

The 1,000 simulated incremental costs and QALYs from the PSA (Figures 3A and 3B) show considerable uncertainty in the base-case results. This uncertainty is apparent in both figures as the spread of estimates crosses two or three quadrants of the cost-effectiveness plane.

Model 2: unobstructed coronary arteries

Table 2 reports the results of the DSAs for Model 2. Reducing the diagnostic accuracy of CMR (DSA 1) and increasing the cost of CMR (DSA 6) had the greatest impact on the mean incremental cost between CMR with standard ECHO and standard ECHO alone. Altering QALYs (DSAs 8, 9 and 10), had the greatest impact on the mean incremental QALYs between the arms. However, in all DSAs conducted, CMR with standard ECHO remained both more costly and more effective than standard ECHO alone, resulting in ICERs that were consistently above the accepted cost-effectiveness threshold.

For peer review only - http://bmjopen.bmj.com/site/about/guidelines.xhtml

Threshold analyses show that if the cost of CMR reduced to £166 (from a base-case value of £264), there would be no difference in the expected costs between strategies.

Similar to Model 1, the 1,000 simulated incremental costs and QALYs (Figure 3C) indicate considerable uncertainty in the base-case results; again, the estimates are spread across three quadrants of the cost-effectiveness plane.

DISCUSSION

We developed economic decision models to identify key drivers of cost-effectiveness of CMR compared to 'current practice' in two subgroups of patients who activate the PPCI pathway: multi-vessel disease (Model 1) and unobstructed coronary arteries (Model 2). 'Current practice' for ischemia testing in multi-vessel disease varies widely. The results of both models suggest that differences in QALYs between strategies are small and, therefore, the results are largely driven by modest differences in costs.

In Model 1, only 35% of patients receiving ischaemia testing truly had ischaemia, therefore the majority of patients received an expensive test without needing further treatment. If more patients needed revascularisation the expected cost of the FFR strategy would reduce as potential economies of scale would result from performing the ischaemia test and secondary revascularisation concurrently. In Model 2, the reduction in costs associated with treating fewer patients for MI if CMR were to be introduced only partially compensated for the additional cost of CMR. DSAs for both models identified the diagnostic accuracy of the tests as the key driver of cost-effectiveness. In Model 1, the costs of ischaemia testing and QALYs associated with MACE/ no MACE also influenced cost-effectiveness, but the latter factors had minimal impact compared to altering the diagnostic accuracy of CMR and FFR. For Model 2, the cost of CMR, the QALYs associated with MACE/ no MACE, and the proportion of patients with MACE who die, influenced cost-effectiveness results.

Overall, we have identified the sensitivity and specificity of the tests as the key drivers of cost-effectiveness in both models. It is likely that any business case to introduce CMR for all patients who activate the PPCI pathway would require accurate estimates of these parameters to be collected in future studies.

In terms of limitations of our study, first, many parameter estimates were obtained from a single study conducted outside the UK, where practices may differ. Estimates from

For peer review only - http://bmjopen.bmj.com/site/about/guidelines.xhtml

different patient groups were sometimes used to populate the model. For example, in Model 1 the diagnostic accuracy of stress ECHO was derived from a study diagnosing significant coronary artery disease, rather than PPCI patients.[31] Despite these potential limitations, our model represents the best available evidence.

Second, for the base-case analysis of Model 1, CMR and FFR were treated as reference standards, assuming 100% sensitivity and specificity. We considered identical values for CMR and FFR as a reasonable starting point; while FFR is the reference standard for detecting ischaemia in patients with stable coronary artery disease, there is uncertainty in whether this also applies to a STEMI population with multi-vessel disease. There is no gold standard test for ischaemia in this population with which to compare. However, it is important to note that varying the sensitivity and specificity in DSAs for Model 1 had large impacts on cost-effectiveness results. Similarly, for Model 2, reducing the diagnostic accuracy of CMR with standard ECHO compared to standard ECHO doubled the ICER.

Third, resource use was estimated for patients in both models based on standard patient pathways described by clinical experts, as individual patient data from routine sources (e.g. Hospital Episode Statistics) were not available at the time of analysis. This may underestimate the variability between individual patients.

Finally, there was uncertainty around the utility decrement of 0.05 for patients with MACE compared to those without MACE.[41] Although this decrement has been used by others, it is not based on primary data.[42] In addition, utility estimates for patients with unobstructed coronary arteries were not available requiring the use of utility estimates from a population with STEMI having PPCI in Model 2. We considered these assumptions to be reasonable given (a) quality-of-life using the Short Form-36 was similar for patients with unobstructed coronary arteries and a control group of MI patients with coronary heart disease,[43] and (b) we varied QALYs in the DSAs.

Our cost-effectiveness models have highlighted that the diagnostic accuracies of the tests (CMR and FFR, and CMR) are key drivers of the relative cost-effectiveness of management strategies based on these tests when compared to ‘current practice’ for patients with multi-vessel disease and unobstructed coronaries respectively. In the base-case analyses for patients with multi-vessel disease, FFR was not cost-effective relative to stress ECHO, and was more costly than a CMR strategy. For patients with stable angina, the MR-INFORM trial showed that CMR-guided management is non-inferior for MACE at one-year compared

For peer review only - http://bmjopen.bmj.com/site/about/guidelines.xhtml

with invasive angiography and FFR.[44] It is, therefore, concerning that there has been rapid adoption of FFR for detecting ischaemia during our study despite a lack of evidence of benefit over CMR; this may reflect the lack of capacity for CMR nationally. Future research should seek to quantify the relative diagnostic accuracy of CMR and FFR compared to stress ECHO, and versus each other. The fact that both CMR and FFR testing are regarded as reference standards in clinical practice and no superior standard is recognised may mean that the cost-effectiveness of CMR compared to FFR can only be tested in a randomised controlled trial, in which the impact of the ischaemia tests on patients' care pathways could be directly observed.

For peer review only

For peer review only - http://bmjopen.bmj.com/site/about/guidelines.xhtml

ACKNOWLEDGEMENTS

We would like to thank Alice Redfern, who assisted with the literature searches for parameter estimates for the models, and Danielle Bargo, who assisted with the design of the health economic model structures.

AUTHOR CONTRIBUTIONS

EAS designed the study, extracted, analysed and interpreted all the data, critically reviewed and edited the draft article and gave final approval of this version to be published.

BD assisted with the interpretation of the data, drafted and revised this article and gave final approval of this version to be published.

MP conceptualised the study, assisted with its design and the interpretation of data, critically reviewed and edited the draft article and gave final approval of this version to be published.

BCR conceptualised the study, assisted with its design and the interpretation of data, critically reviewed and edited the draft article and gave final approval of this version to be published.

CBD conceptualised the study, assisted with its design and the interpretation of data, critically reviewed and edited the draft article and gave final approval of this version to be published.

SD assisted with the study design and the interpretation of data, critically reviewed and edited the draft article and gave final approval of this version to be published.

JPG assisted with the interpretation of data, critically reviewed and edited the draft article and gave final approval of this version to be published.

RAA assisted with the interpretation of data, critically reviewed and edited the draft article and gave final approval of this version to be published.

SW conceptualised the study, assisted with its design and the interpretation of data, critically reviewed and edited the draft article and gave final approval of this version to be published.

FUNDING

This research was funded by the National Institute for Health Research (NIHR), Health Services and Delivery Research Programme (ref 11/2003/58). Barnaby C. Reeves, Chiara Bucciarelli-Ducci and the Cardiac MRI Unit are also partly funded through the NIHR

For peer review only - http://bmjopen.bmj.com/site/about/guidelines.xhtml

Biomedical Research Centre at University Hospitals Bristol NHS Foundation Trust and the University of Bristol. The funder had no involvement in the study design; in the collection, analysis and interpretation of the data; in the writing of the paper; and in the decision to submit the paper for publication.

DISCLAIMER

The views expressed are those of the authors and not necessarily those of the NHS, the NIHR or the Department of Health and Social Care.

COMPETING INTERESTS

Chiara Bucciarelli-Ducci has received personal fees from Circle Cardiovascular Imaging. Barnaby C. Reeves reports former membership of the Health Technology Assessment Commissioning Board (up to 31 March 2016) and Health Technology Assessment Efficient Study Designs Board (October 2014 to December 2014). He also reports current membership of the Health Technology Assessment IP Methods Group and Systematic Reviews Programme Advisory Group (Systematic Reviews NIHR Cochrane Incentive Awards and Systematic Review Advisory Group). Beyond this, all authors have no competing interests, except support from the NIHR grant as detailed in the funding statement.

ETHICS

Since the health economic models were populated with data from secondary sources, ethical approval was not required for this study.

PATIENT CONSENT

Patient consent was not required.

DATA SHARING STATEMENT

No additional data available.

For peer review only

For peer review only - http://bmjopen.bmj.com/site/about/guidelines.xhtml

REFERENCES

1. Kumar A, Cannon CP. Acute coronary syndromes: diagnosis and management, part I. *Mayo Clin Proc* 2009;84(10):917-38.
2. Cardarelli F, Bellasi A, Ou FS, et al. Combined impact of age and estimated glomerular filtration rate on in-hospital mortality after percutaneous coronary intervention for acute myocardial infarction (from the American College of Cardiology National Cardiovascular Data Registry). *Am J Cardiol* 2009;103(6):766-71.
3. Goldstein JA, Demetriou D, Grines CL, et al. Multiple complex coronary plaques in patients with acute myocardial infarction. *N Engl J Med* 2000;343(13):915-22.
4. Lee JH, Park HS, Chae SC, et al. Predictors of six-month major adverse cardiac events in 30-day survivors after acute myocardial infarction (from the Korea Acute Myocardial Infarction Registry). *Am J Cardiol* 2009;104(2):182-9.
5. Rasoul S, Ottervanger JP, de Boer MJ, et al. Predictors of 30-day and 1-year mortality after primary percutaneous coronary intervention for ST-elevation myocardial infarction. *Coron Artery Dis* 2009;20(6):415-21.
6. Toma M, Buller CE, Westerhout CM, et al. Non-culprit coronary artery percutaneous coronary intervention during acute ST-segment elevation myocardial infarction: insights from the APEX-AMI trial. *Eur Heart J* 2010;31(14):1701-7.
7. Vlaar PJ, Mahmoud KD, Holmes DR, Jr., et al. Culprit vessel only versus multivessel and staged percutaneous coronary intervention for multivessel disease in patients presenting with ST-segment elevation myocardial infarction: a pairwise and network meta-analysis. *J Am Coll Cardiol* 2011;58(7):692-703.
8. Larsen AI, Galbraith PD, Ghali WA, et al. Characteristics and outcomes of patients with acute myocardial infarction and angiographically normal coronary arteries. *Am J Cardiol* 2005;95(2):261-3.
9. Meryon I, Patel N, Millane T, et al. Normal coronary angiography and primary percutaneous coronary intervention for ST elevation myocardial infarction: a literature review and audit findings. *Int J Clin Pract* 2010;64(9):1245-51.
10. Roongsritong C, Warraich I, Bradley C. Common causes of troponin elevations in the absence of acute myocardial infarction: incidence and clinical significance. *Chest* 2004;125(5):1877-84.
11. Sarda L, Colin P, Boccara F, et al. Myocarditis in patients with clinical presentation of myocardial infarction and normal coronary angiograms. *J Am Coll Cardiol* 2001;37(3):786-92.
12. Dokainish H, Pillai M, Murphy SA, et al. Prognostic implications of elevated troponin in patients with suspected acute coronary syndrome but no critical epicardial coronary disease: a TACTICS-TIMI-18 substudy. *J Am Coll Cardiol* 2005;45(1):19-24.
13. Assomull RG, Lyne JC, Keenan N, et al. The role of cardiovascular magnetic resonance in patients presenting with chest pain, raised troponin, and unobstructed coronary arteries. *Eur Heart J* 2007;28(10):1242-9.
14. Codreanu A, Djaballah W, Angioi M, et al. Detection of myocarditis by contrast-enhanced MRI in patients presenting with acute coronary syndrome but no coronary stenosis. *J Magn Reson Imaging* 2007;25(5):957-64.
15. Laissy JP, Hyafil F, Feldman LJ, et al. Differentiating acute myocardial infarction from myocarditis: diagnostic value of early- and delayed-perfusion cardiac MR imaging. *Radiology* 2005;237(1):75-82.

For peer review only - http://bmjopen.bmj.com/site/about/guidelines.xhtml

16. Leurent G, Langella B, Fougerou C, et al. Diagnostic contributions of cardiac magnetic resonance imaging in patients presenting with elevated troponin, acute chest pain syndrome and unobstructed coronary arteries. *Arch Cardiovasc Dis* 2011;104(3):161-70.
17. Stensaeth KH, Fossum E, Hoffmann P, et al. Clinical characteristics and role of early cardiac magnetic resonance imaging in patients with suspected ST-elevation myocardial infarction and normal coronary arteries. *Int J Cardiovasc Imaging* 2011;27(3):355-65.
18. Antony R, Daghem M, McCann GP, et al. Cardiovascular magnetic resonance activity in the United Kingdom: a survey on behalf of the British Society of Cardiovascular Magnetic Resonance. *J Cardiovasc Magn Reson* 2011;13:57.
19. Ibanez B, James S, Agewall S, et al. 2017 ESC Guidelines for the management of acute myocardial infarction in patients presenting with ST-segment elevation: The Task Force for the management of acute myocardial infarction in patients presenting with ST-segment elevation of the European Society of Cardiology (ESC). *Eur Heart J* 2018;39(2):119-77.
20. Lockie T, Ishida M, Perera D, et al. High-resolution magnetic resonance myocardial perfusion imaging at 3.0-Tesla to detect hemodynamically significant coronary stenoses as determined by fractional flow reserve. *J Am Coll Cardiol* 2011;57(1):70-5.
21. Watkins S, McGeoch R, Lyne J, et al. Validation of magnetic resonance myocardial perfusion imaging with fractional flow reserve for the detection of significant coronary heart disease. *Circulation* 2009;120(22):2207-13.
22. Bhattacharyya S, Chehab O, Khattar R, et al. Stress echocardiography in clinical practice: a United Kingdom National Health Service Survey on behalf of the British Society of Echocardiography. *European Heart Journal - Cardiovascular Imaging* 2014;15(2):158-63.
23. Brierley R, Pufulete M, Harris J, et al. Developing a UK registry to investigate the role of cardiovascular magnetic resonance (CMR) in patients who activate the primary percutaneous coronary intervention (PPCI) pathway: a multicentre, feasibility study linking routinely collected electronic patient data. *BMJ Open* 2018;8(3):e018987.
24. National Institute for Health and Care Excellence (NICE). Guide to the Methods of Technology Appraisal 2013. London: NICE 2013.
25. Department of Health. National Schedule of Reference Costs 2015-16. London: Department of Health 2016.
26. Joint Formulary Committee. British National Formulary No. 73. London: British Medical Association and Royal Pharmaceutical Society of Great Britian 2017.
27. The Center for the Evaluation of Value and Risk in Health Tufts Medical Center. Cost-Effectiveness Analysis (CEA) Registry. URL: http://healtheconomics.tuftsmedicalcenter.org/cear4/Home.aspx. (accessed 06/04/2017).
28. Dastidar AG, Rodrigues JC, Baritussio A, et al. MRI in the assessment of ischaemic heart disease. *Heart* 2016;102(3):239-52.
29. Pasupathy S, Air T, Dreyer RP, et al. Systematic review of patients presenting with suspected myocardial infarction and nonobstructive coronary arteries. *Circulation* 2015;131(10):861-70.
30. Tornvall P, Gerbaud E, Behaghel A, et al. Myocarditis or "true" infarction by cardiac magnetic resonance in patients with a clinical diagnosis of myocardial infarction without obstructive coronary disease: A meta-analysis of individual patient data. *Atherosclerosis* 2015;241(1):87-91.

For peer review only - http://bmjopen.bmj.com/site/about/guidelines.xhtml

31. Gurunathan S, Young G, Parsons G, et al. 132 Diagnostic Accuracy of Stress Echocardiography Compared with Invasive Coronary Angiography with Fractional Flow Reserve for The Diagnosis of Haemodynamically Significant Cad in Patients with Known or Suspected CAD. *Heart* 2016;102(Suppl 6):A94-A95.
32. Dastidar AG, Rodrigues JCL, Johnson TW, et al. Myocardial Infarction With Nonobstructed Coronary Arteries: Impact of CMR Early After Presentation. *JACC Cardiovasc Imaging* 2017;10(10 Pt A):1204-06.
33. Tonino PA, Fearon WF, De Bruyne B, et al. Angiographic versus functional severity of coronary artery stenoses in the FAME study fractional flow reserve versus angiography in multivessel evaluation. *J Am Coll Cardiol* 2010;55(25):2816-21.
34. Smits PC, Abdel-Wahab M, Neumann FJ, et al. Fractional Flow Reserve-Guided Multivessel Angioplasty in Myocardial Infarction. *N Engl J Med* 2017;376(13):1234-44.
35. Chopard R, Jehl J, Dutheil J, et al. Evolution of acute coronary syndrome with normal coronary arteries and normal cardiac magnetic resonance imaging. *Arch Cardiovasc Dis* 2011;104(10):509-17.
36. Gerbaud E, Harcaut E, Coste P, et al. Cardiac magnetic resonance imaging for the diagnosis of patients presenting with chest pain, raised troponin, and unobstructed coronary arteries. *Int J Cardiovasc Imaging* 2012;28(4):783-94.
37. Kang WY, Jeong MH, Ahn YK, et al. Are patients with angiographically near-normal coronary arteries who present as acute myocardial infarction actually safe? *Int J Cardiol* 2011;146(2):207-12.
38. Pathik B, Raman B, Mohd Amin NH, et al. Troponin-positive chest pain with unobstructed coronary arteries: incremental diagnostic value of cardiovascular magnetic resonance imaging. *Eur Heart J Cardiovasc Imaging* 2016;17(10):1146-52.
39. Lindahl B, Baron T, Erlinge D, et al. Medical Therapy for Secondary Prevention and Long-Term Outcome in Patients With Myocardial Infarction With Nonobstructive Coronary Artery Disease. *Circulation* 2017;135(16):1481-89.
40. Aasa M, Henriksson M, Dellborg M, et al. Cost and health outcome of primary percutaneous coronary intervention versus thrombolysis in acute ST-segment elevation myocardial infarction-Results of the Swedish Early Decision reperfusion Study (SWEDES) trial. *Am Heart J* 2010;160(2):322-8.
41. Nam J, Briggs A, Layland J, et al. Fractional flow reserve (FFR) versus angiography in guiding management to optimise outcomes in non-ST segment elevation myocardial infarction (FAMOUS-NSTEMI) developmental trial: cost-effectiveness using a mixed trial- and model-based methods. *Cost Eff Resour Alloc* 2015;13:19.
42. Palmer S, Sculpher M, Philips Z, et al. A cost-effectiveness model comparing alternative management strategies for the use of glycoprotein IIb/IIIa antagonists in non-st-elevation acute coronary syndrome. Report to the National Institute for Clinical Excellence 2002.
43. Daniel M, Agewall S, Caidahl K, et al. Effect of Myocardial Infarction With Nonobstructive Coronary Arteries on Physical Capacity and Quality-of-Life. *Am J Cardiol* 2017;120(3):341-46.
44. MR-INFORM: Stress Perfusion Imaging to Guide the Management of Stable CAD Patients. American College of Cardiology Annual Scientific Session; 2017 17/03/2017; Washington, DC.

For peer review only - http://bmjopen.bmj.com/site/about/guidelines.xhtml

FIGURE LEGENDS

Figure 1. Model 1 Structure - Patients with multi-vessel disease. Patient pathways for FFR and stress ECHO are identical to those for CMR. CMR, Cardiovascular Magnetic Resonance; echo, Echocardiography; FFR; Fractional flow reserve; MACE, Major adverse cardiac events.

Figure 2. Model 2 Structure - Patients with unobstructed coronaries. CMR, Cardiovascular Magnetic Resonance; echo, Echocardiography; MACE, Major adverse cardiac events; MI, Myocardial infarction.

Figure 3. (A) Plot of the 1000 simulated cost and QALY differences from the PSA for CMR versus stress ECHO; (B) Plot of the 1000 simulated cost and QALY differences from the PSA for FFR versus stress ECHO; (C) Plot of the 1000 simulated cost and QALY differences from the PSA for standard ECHO and CMR versus standard ECHO. CMR, Cardiovascular Magnetic Resonance; ECHO, Echocardiography; FFR; Fractional flow reserve; PSA, Probabilistic sensitivity analyses; QALY, Quality-adjusted life years.

For peer review only

For peer review only - http://bmjopen.bmj.com/site/about/guidelines.xhtml

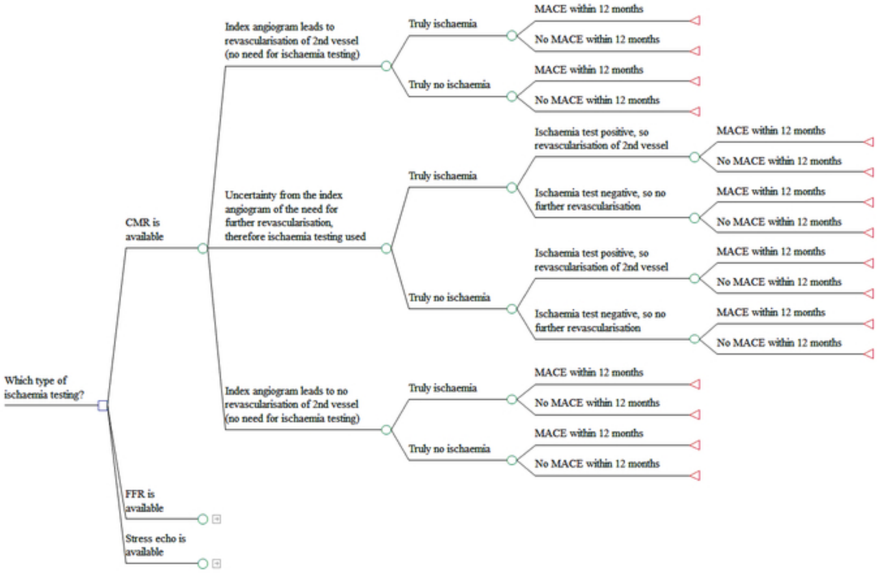

Figure 1. Model 1 Structure - Patients with multi-vessel disease.

65x38mm (300 x 300 DPI)

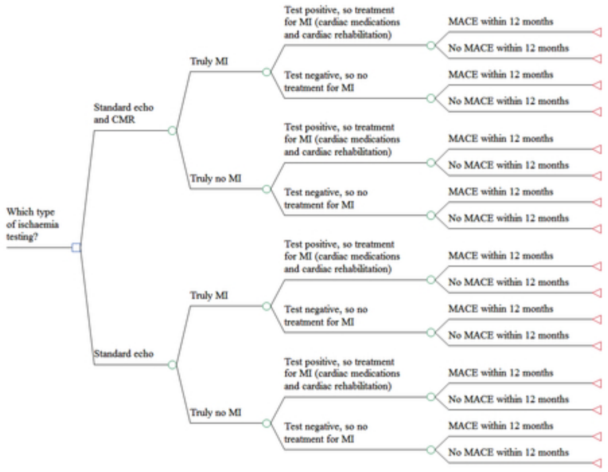

Figure 2. Model 2 Structure - Patients with unobstructed coronaries.

62x30mm (300 x 300 DPI)

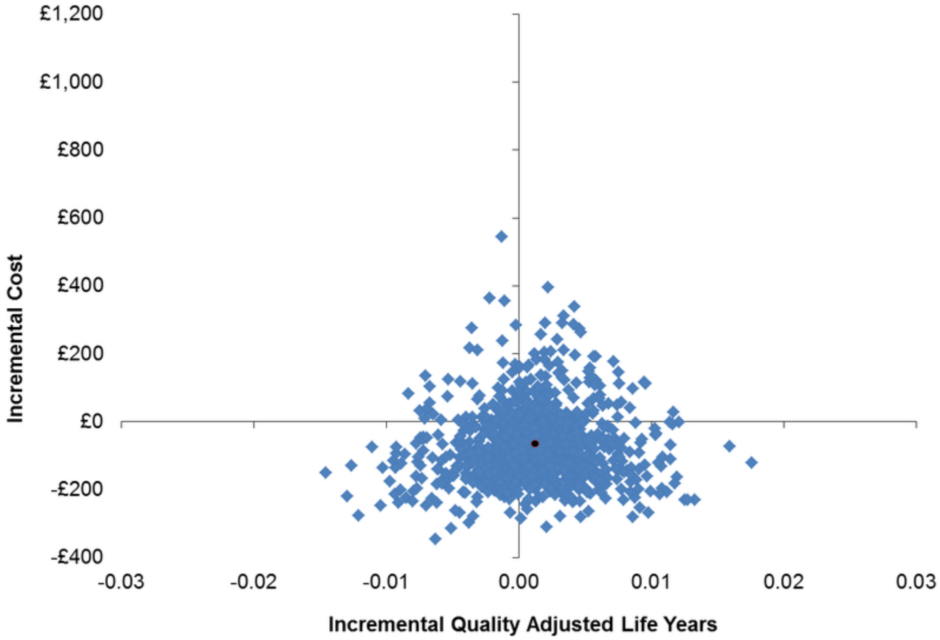

Figure 3A. Plot of the 1000 simulated cost and QALY differences from the PSA for CMR versus stress ECHO;

72x49mm (300 x 300 DPI)

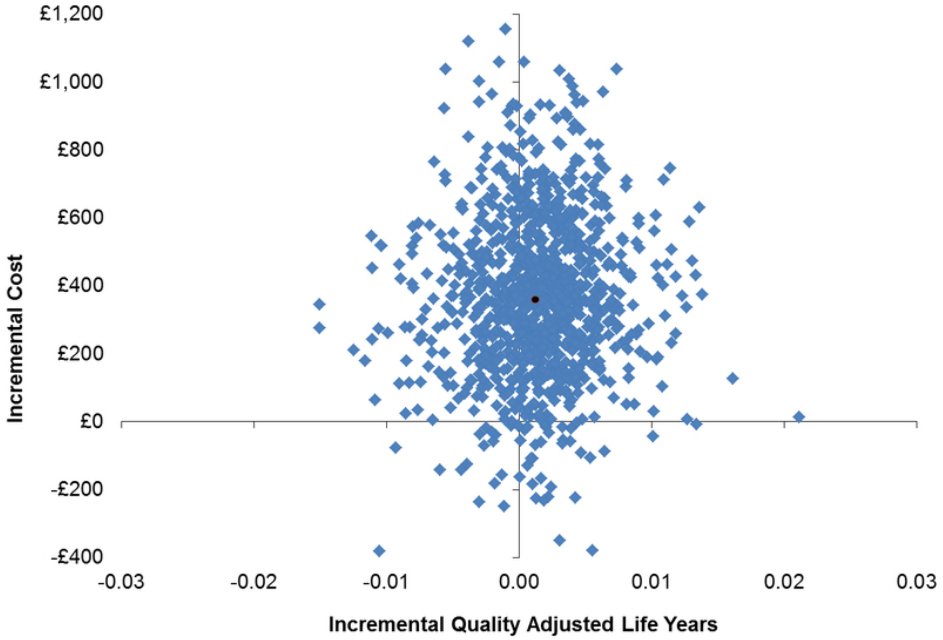

Figure 3B. Plot of the 1000 simulated cost and QALY differences from the PSA for FFR versus stress ECHO

72x49mm (300 x 300 DPI)

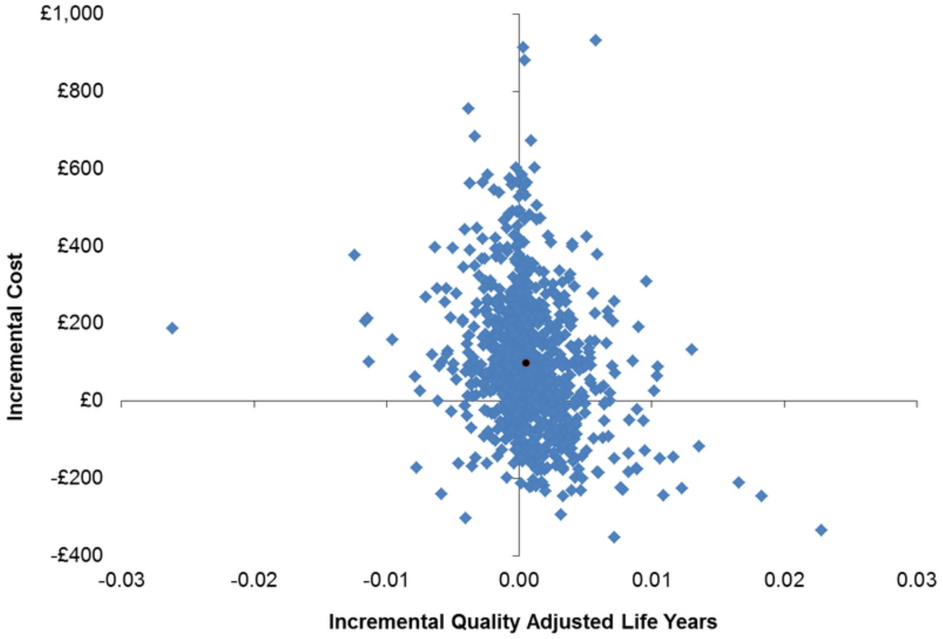

Figure 3C. Plot of the 1000 simulated cost and QALY differences from the PSA for standard ECHO and CMR versus standard ECHO

72x49mm (300 x 300 DPI)

SUPPLEMENTARY INFORMATION

Table of Contents

Appendix A – Model parameters, sources and assumptions for model 1 (multi-vessel disease) 2

Appendix B – Model parameters, sources and assumptions for model 2 (unobstructed coronary arteries) 6

Appendix C – Deterministic one-way sensitivity analyses for model 1 (multi-vessel disease) 9

Appendix D – Deterministic one-way sensitivity analyses for model 2 (unobstructed coronary arteries) 10

References 11

For peer review only - http://bmjopen.bmj.com/site/about/guidelines.xhtml

APPENDIX A – MODEL PARAMETERS, SOURCES AND ASSUMPTIONS FOR MODEL 1 (MULTI-VESSEL DISEASE)

**Testing and clinical parameters**			
Branch of model	Base-case value (Positive/total)	Distribution for PSA	Source
For each ischaemia testing option			
Probability index angiogram leads to revascularisation of second vessel	0.30 (30/100)*	Dirichlet	Expert opinion within the PIPA study team
Probability of uncertainty following index angiogram and need for ischaemia testing	0.60 (60/100)*		
Probability index angiogram leads to no revascularisation of second vessel	0.10 (10/100)*		
Index angiogram leads to revascularisation of second vessel			
Probability of true ischaemia	0.84 (598/709)	Beta	FAME[1]
Probability of MACE by 12 months | true ischaemia	0.09 (18/202)†	Beta	Smits 2017[2]
Probability of MACE by 12 months | truly no ischaemia	0.13 (54/432)†	Beta	Smits 2017[2]
Uncertainty following index angiogram and need for ischaemia testing			
Probability of true ischaemia	0.35 (218/620)	Beta	FAME[1]
Index angiogram leads to no revascularisation of second vessel			
Probability of true ischaemia	0.35 (218/620)	Beta	FAME[1]
Probability of MACE by 12 months | true ischaemia	0.31 (71/231)†	Beta	Smits 2017[2]
Probability of MACE by 12 months | truly no ischaemia	0.13 (54/432)†	Beta	Smits 2017[2]
CMR or FFR			
Uncertainty following index angiogram and need for ischaemia testing, True ischaemia			
Probability of ischaemia test positive and revascularisation	1§	Beta	
Probability of MACE by 12 months | revascularisation	0.09 (18/202)†	Beta	Smits 2017[2]
Probability of MACE by 12 months | no revascularisation	0.31 (71/231)†#	Beta	Smits 2017[2]
Uncertainty following index angiogram and need for ischaemia testing, Truly no ischaemia			
Probability of ischaemia test positive and revascularisation	0§	Beta	
Probability of MACE by 12 months | revascularisation	0.13 (54/432)†#	Beta	Smits 2017[2]

For peer review only - http://bmjopen.bmj.com/site/about/guidelines.xhtml

Probability of MACE by 12 months | no revascularisation	0.13 (54/432)†	Beta	Smits 2017[2]
Stress ECHO			
Uncertainty following index angiogram and need for ischaemia testing, True ischaemia			
Probability of ischaemia test positive and revascularisation	0.70 (32/46)	Beta	Gurunathan 2016[3]
Probability of MACE by 12 months | revascularisation	0.09 (18/202)†	Beta	Smits 2017[2]
Probability of MACE by 12 months | no revascularisation	0.31 (71/231)†	Beta	Smits 2017[2]
Uncertainty following index angiogram and need for ischaemia testing, Truly no ischaemia			
Probability of ischaemia test positive and revascularisation	0.23 (39/171)	Beta	Gurunathan 2016[3]
Probability of MACE by 12 months | revascularisation	0.13 (54/432)†	Beta	Smits 2017[2]
Probability of MACE by 12 months | no revascularisation	0.13 (54/432)†	Beta	Smits 2017[2]
**Cost parameters**			
Resource	Unit cost (SE)**	Distribution for PSA	Source
Revascularisation of second vessel (assumed to be PCI)	£2,682 (533)	Gamma	NHS Reference Costs 2015/16.[4] Elective Inpatient. Weighted average of codes EY40 and EY41, Standard / Complex Percutaneous Transluminal Coronary Angioplasty.
CMR	£264 (154)	Gamma	NHS Reference Costs 2015/16.[4] Diagnostic Imaging. Weighted average of all Cardiac Magnetic Resonance Imaging Scan codes.
Angiogram and FFR	£1,340 (374)	Gamma	NHS Reference Costs 2015/16.[4] Day case. Weighted average of code EY42, Complex Cardiac Catheterisation.
Angiogram, FFR and revascularisation (as a single catheter laboratory admission)	£2,971 (594)	Gamma	Cost of revascularisation above, and angiogram and FFR above, minus the cost of an angiogram, £1,051 (NHS Reference Costs 2015/16.[4] Day case. Weighted average of code EY43, Standard Cardiac Catheterisation)
Stress ECHO	£182 (3)	Gamma	NHS Reference Costs 2015/16.[4] Outpatient procedures. Cardiology. EY50Z, Complex Echocardiogram.
MACE	£3,251 (650)	Gamma	The proportion of each component of MACE was taken from Smits, 2017,[2] and a weighted average of the costs of

For peer review only - http://bmjopen.bmj.com/site/about/guidelines.xhtml

			each component was calculated, based on the unit costs below.
Revascularisation (PCI)	£2,682		As above
Revascularisation (CABG)	£10,944		NHS Reference Costs 2015/16.[4] Elective Inpatient. Weighted average of codes ED26, ED27, ED28 Complex/ Major/ Standard Coronary Artery Bypass Graft
Myocardial infarction (MI)	£2,177		NHS Reference Costs 2015/16.[4] Non-elective long stay. Weighted average of code EB10A, Actual or Suspected Myocardial Infarction.
Stroke	£3,723		NHS Reference Costs 2015/16.[4] Non-elective long stay. Weighted average of code AA22, Cerebrovascular Accident, Nervous System Infections or Encephalopathy.
Death	£0		
Cardiac rehabilitation	£364 (73)	Gamma	Assume offered eight sessions, but only 50% update, so cost four sessions (one first and three follow up appointments, individual costs below)
First appointment	£97		NHS Reference Costs 2015/16.[4] Consultant Led. 327, Cardiac Rehabilitation. Non-Admitted Face to Face Attendance, First.
Follow up appointment	£89		NHS Reference Costs 2015/16.[4] Consultant Led. 327, Cardiac Rehabilitation. Non-Admitted Face to Face Attendance, Follow-Up.
Cardiac medications for treatment of MI (all once daily by mouth). Assumed given for 12 months (or 6 months for patients who die).			
Aspirin (75mg)	£0.04		BNF 73, March 2017[5]
Prasugrel (5mg)	£1.70		BNF 73, March 2017[5]
Atorvastatin (80mg)	£0.07		BNF 73, March 2017[5]
Bisoprolol (2.5mg)	£0.02		BNF 73, March 2017[5]
Ramipril (2.5mg)	£0.04		BNF 73, March 2017[5]
Total daily cost	£1.87		
Total annual cost	£683 (137)	Gamma	
Outpatient follow up appointment (assumed to occur at 4-6 weeks and 6 months post-discharge)	£122 (22)	Gamma	NHS Reference Costs 2015/16.[4] Consultant led appointments. Non-Admitted Face to Face Attendance, Follow-Up. Cardiology.
Additional healthcare costs to one year	£2221 (444)	Gamma	Office of Health Economics estimate of the annual cost per person of NHS care inflated to 2015/16 prices using the

For peer review only

			hospital and community health services inflation index[6, 7] For patients who die, costs are assumed to be half of this (£1111).
Additional healthcare costs to one year for those with MACE	£263 (53)	Gamma	MI and stroke were assumed to be associated with continuing care costs post discharge. Costs from Greenhalgh (2011),[8] were inflated to 2015/16 prices.[6] Separate costs were provided for disabling and non-disabling stroke; according to Davies (2006) 58% of strokes are disabling,[9] and this percentage was used to weight the two continuing care costs for stroke. Additional costs to one year associated with MACE were calculated by weighting estimates of the annual costs of MI and stroke by the number of patients with these complications across the number of patients with MACE, from Smits (2017).[2]

**Quality-adjusted life years**			
Patient group	Mean (SE) †† QALYs to one year	Distribution for PSA	Source
No revascularisation of a second vessel and MACE	0.686 (0.09)	Beta	Calculations and sources described in the text
No revascularisation of a second vessel and no MACE	0.768 (0.09)	Beta	
Revascularisation of a second vessel and MACE	0.684 (0.09)	Beta	
Revascularisation of a second vessel and no MACE	0.766 (0.09)	Beta	

* To incorporate uncertainty around the percentage of patients in each of these three groups estimated by experts on the study team, it was assumed that experts were estimating the number of patients out of 100 in each group.
† For further information on the estimates of MACE, please see the final paragraph under 'Model probabilities'.
§ In the base case analyses, CMR and FFR are assumed to have perfect sensitivity and specificity, but these parameters are incorporated into the model so that alternative assumptions can be tested in sensitivity analyses.
# These probabilities are not required in the base case analysis (since the probability in the preceding branch is zero), but are included here as they are required in sensitivity analyses.
** NHS Reference Costs provide upper and lower quartiles around mean estimates; the difference between the mean and the upper quartile was captured, and used as a crude estimate of standard error in the PSA. Where this was not possible, the standard error was assumed to be 20% of the mean cost.
†† A standard error of 0.09 was assumed, based on a previous study.[10]

CABG, Coronary Artery Bypass Graft; CMR, Cardiovascular Magnetic Resonance; ECHO, Echocardiography; FFR; Fractional flow reserve; MACE, Major adverse cardiac events; MI, Myocardial infarction; PCI, Percutaneous coronary intervention; PSA, Probabilistic sensitivity analysis; QALYs, Quality-adjusted life years; SE, Standard error

For peer review only

APPENDIX B – MODEL PARAMETERS, SOURCES AND ASSUMPTIONS FOR MODEL 2 (UNOBSTRUCTED CORONARY ARTERIES)

**Testing and clinical parameters**			
Branch of model	Base-case value (Positive/total)	Distribution for PSA	Source
For both arms			
Probability of truly having had an myocardial infarction (MI)	0.24 (429/1801)	Beta	Pasupathy 2015[11]
Standard ECHO and CMR			
Probability of ischaemia test positive and treatment for MI | MI	1*	Beta	
Probability of MACE within 12 months | treatment for MI	0.21†	Beta	Kang 2011,[12] Pathik 2016[13]
Probability of MACE within 12 months | no treatment for MI	0.26†§	Beta	Kang 2011,[12] Pathik 2016,[13] Lindahl 2017[14]
Probability of ischaemia test positive and treatment for MI | no MI	0*	Beta	
Probability of MACE within 12 months | treatment for MI	0.03†§	Beta	Kang 2011,[12] Pathik 2016[13]
Probability of MACE within 12 months | no treatment for MI	0.03†	Beta	Kang 2011,[12] Pathik 2016[13]
Standard ECHO			
Probability of ischaemia test positive and treatment for MI | MI	0.47 (25/53)#	Beta	Dastidar 2017[15]
Probability of MACE within 12 months | treatment for MI	0.21†	Beta	Kang 2011,[12] Pathik 2016[13]
Probability of MACE within 12 months | no treatment for MI	0.26†	Beta	Kang 2011,[12] Pathik 2016,[13] Lindahl 2017[14]
Probability of ischaemia test positive and treatment for MI | no MI	0.62 (93/151)#	Beta	Dastidar 2017[15]
Probability of MACE within 12 months | treatment for MI	0.03†	Beta	Kang 2011,[12] Pathik 2016[13]
Probability of MACE within 12 months | no treatment for MI	0.03†	Beta	Kang 2011,[12] Pathik 2016[13]

**Cost parameters**			
Resource	Unit cost (SE)**	Distribution for PSA	Source
Standard ECHO	£72 (22)	Gamma	NHS Reference Costs 2015/16.[4] Diagnostic Imaging. Outpatient. RD51A, Simple Echocardiogram, 19 years and over.

For peer review only - http://bmjopen.bmj.com/site/about/guidelines.xhtml

CMR	£264 (154)	Gamma	NHS Reference Costs 2015/16.[4] Diagnostic Imaging. Weighted average of all Cardiac Magnetic Resonance Imaging Scan codes.
Cardiac rehabilitation	£364 (73)	Gamma	Assume offered eight sessions, but only 50% update, so cost four sessions (one first and three follow up appointments, individual costs below)
First appointment	£97		NHS Reference Costs 2015/16.[4] Consultant Led. Cardiac Rehabilitation. Non-Admitted Face to Face Attendance, First.
Follow up appointment	£89		NHS Reference Costs 2015/16.[4] Consultant Led. Cardiac Rehabilitation. Non-Admitted Face to Face Attendance, Follow-Up.
Cardiac medications for treatment of MI (all once daily by mouth)			
Aspirin (75mg)	£0.04		BNF 73, March 2017[5]
Clopidogrel (75mg)	£0.05		BNF 73, March 2017[5]
Atorvastatin (80mg)	£0.07		BNF 73, March 2017[5]
Bisoprolol (2.5mg)	£0.02		BNF 73, March 2017[5]
Ramipril (2.5mg)	£0.04		BNF 73, March 2017[5]
Total daily cost	£0.22		
Total annual cost	£80 (16)	Gamma	
Treatment for non-MI cause (cardiac medications) – total annual cost	£40 (8)	Gamma	Assume half of these patients are taken off cardiac medications above (these medications are given for cardiomyopathy but not for myocarditis)
Outpatient follow up appointment (assumed to occur at 4-6 weeks post-discharge)	£122 (22)	Gamma	NHS Reference Costs 2015/16.[4] Consultant led appointments. Non-Admitted Face to Face Attendance, Follow-Up. Cardiology.
MACE	£2,808 (562)	Gamma	The proportion of each component of MACE was taken from Pathik,[13] and a weighted average of the costs of each component was calculated, based on the unit costs provided in Appendix A. Given the small number of MACE events, calculations were based on MI and non-MI patients combined.
Additional healthcare costs to one year	£2221 (444)	Gamma	Office of Health Economics estimate of the annual cost per person of NHS care inflated to 2015/16 prices using the hospital and community health services inflation index[6, 7] For patients who die, costs are assumed to

For peer review only

			be half of this (£1111).
Additional healthcare costs to one year for those with MACE	£1602 (320)	Gamma	MI and stroke were assumed to be associated with continuing care costs post discharge. Costs from Greenhalgh (2011),[8] were inflated to 2015/16 prices.[6] Separate costs were provided for disabling and non-disabling stroke; according to Davies (2006) 58% of strokes are disabling,[9] and this percentage was used to weight the two continuing care costs for stroke. Additional costs to one year associated with MACE were calculated by weighting estimates of the annual costs of MI and stroke by the number of patients with these complications across the number of patients with MACE, from Pathik (2017).[13]

**Quality-adjusted life years**			
Patient group	Mean (SE)†† QALYs to one year	Distribution for PSA	Source
MACE within 12 months	0.686 (0.09)	Beta	Calculations and sources described in the text
No MACE within 12 months	0.768 (0.09)	Beta	

* In the base case analyses, standard ECHO combined with CMR is assumed to have perfect sensitivity and specificity, but these parameters are incorporated into the model so that alternative assumptions can be tested in sensitivity analyses.
† A standard error of 0.10 was assumed for the PSA.
§ These probabilities are not required in the base case analysis (since the probability in the preceding branch is zero), but are included here as they are required in sensitivity analyses.
# For further information on how estimates of sensitivity and specificity for standard ECHO were obtained, please see the final paragraph under 'Model probabilities'.
** NHS Reference Costs provide upper and lower quartiles around mean estimates; the difference between the mean and the upper quartile was captured, and used as a crude estimate of standard error in the PSA. Where this was not possible, the standard error was assumed to be 20% of the mean cost.
†† A standard error of 0.09 was assumed, based on a previous study.[10]

CMR, Cardiovascular Magnetic Resonance; ECHO, Echocardiography; MACE, Major adverse cardiac events; MI, Myocardial infarction; PSA, Probabilistic sensitivity analysis; QALYs, Quality-adjusted life years

For peer review only

APPENDIX C – DETERMINISTIC ONE-WAY SENSITIVITY ANALYSES FOR MODEL 1 (MULTI-VESSEL DISEASE)

SA	Parameter varied	Base case	Alternative strategies for sensitivity analysis
1	Probabilities associated with decision on revascularisation from index angiogram	Based on expert opinion	Based on PIPA data
	Probability index angiogram leads to revascularisation of second vessel	0.30 (30/100)	0.11 (78/717)
	Probability of uncertainty following index angiogram and need for ischaemia testing	0.60 (60/100)	0.15 (111/717)
	Probability index angiogram leads to no revascularisation of second vessel	0.10 (10/100)	0.74 (528/717)
2	Sensitivity and specificity of CMR	1 and 1	0.8 (SE 0.1) for each
3	Sensitivity and specificity of FFR	1 and 1	0.8 (SE 0.1) for each
4	Sensitivity and specificity of stress ECHO	0.70 and 0.77 (reported 1-specificity =0.23)	Sensitivity: 0.791 (SE 0.008) and 1-specificity 0.129 (SE 0.007) [54]
5	Probability of MACE	0.09, 0.13, 0.31	All ±0.05
6	Cost of CMR	£264	± 20%
7	Cost of angiogram and FFR	£1340	± 20%
8	QALYs for patients with MACE	0.686 and 0.684	-0.2
9	QALYs for patients with no MACE	0.768 and 0.766	+0.2

CMR, Cardiovascular Magnetic Resonance; ECHO, Echocardiography; FFR, Fractional flow reserve; MACE, Major adverse cardiac events; QALY, Quality-adjusted life years; SA, Sensitivity analysis; SE, Standard error

For peer review only - http://bmjopen.bmj.com/site/about/guidelines.xhtml

APPENDIX D – DETERMINISTIC ONE-WAY SENSITIVITY ANALYSES FOR MODEL 2 (UNOBSTRUCTED CORONARY ARTERIES)

SA	Parameter varied	Base case	Alternative strategies for sensitivity analysis
1	Sensitivity and specificity of standard ECHO plus CMR	1 and 1	0.8 (SE 0.1) for each
2	Sensitivity and specificity of standard ECHO	0.47 and 0.38	0.7 (SE 0.1) for each
3	Probability of MACE (ratio of events in MI:non-MI group =4)	0.21, 0.26, 0.03	0.18, 0.23, 0.05
4	Probability of MACE (ratio=2)	0.21, 0.26, 0.03	0.13, 0.17, 0.06
5	Probability of MACE (ratio=1)	0.21, 0.26, 0.03	0.08, 0.10, 0.08
6	Cost of CMR	£264	± 20%
7	Cost of additional healthcare costs to one year for patients with MACE - increase the proportion who die from 8% to 50%	£1602	£874
8	QALYs for patients with MACE	0.686	-0.2 (0.486)
9	QALYs for patients with no MACE	0.768	+0.2 (0.968)
10	QALYs for patients with MACE - increase the proportion who die from 8% to 50%	0.686	0.525

CMR, Cardiovascular Magnetic Resonance; ECHO, Echocardiography; MACE, Major adverse cardiac events; MI, Myocardial infarction; QALY, Quality-adjusted life years; SA, Sensitivity analysis; SE, Standard error

For peer review only

REFERENCES

1. Tonino PA, Fearon WF, De Bruyne B, et al. Angiographic versus functional severity of coronary artery stenoses in the FAME study fractional flow reserve versus angiography in multivessel evaluation. *J Am Coll Cardiol* 2010;55(25):2816-21.
2. Smits PC, Abdel-Wahab M, Neumann FJ, et al. Fractional Flow Reserve-Guided Multivessel Angioplasty in Myocardial Infarction. *N Engl J Med* 2017;376(13):1234-44.
3. Gurunathan S, Young G, Parsons G, et al. 132 Diagnostic Accuracy of Stress Echocardiography Compared with Invasive Coronary Angiography with Fractional Flow Reserve for The Diagnosis of Haemodynamically Significant Cad in Patients with Known or Suspected CAD. *Heart* 2016;102(Suppl 6):A94-A95.
4. Department of Health. National Schedule of Reference Costs 2015-16. London: Department of Health 2016.
5. Joint Formulary Committee. British National Formulary No. 73. London: British Medical Association and Royal Pharmaceutical Society of Great Britian 2017.
6. Curtis L, Burns A. Unit costs of health and social care 2016. Canterbury: Personal Social Services Research Unit, University of Kent 2016.
7. Hawe E, Cockcroft L. OHE guide to UK health and health care statistics. London: Office of Health Economics 2013.
8. Greenhalgh J, Bagust A, Boland A, et al. Clopidogrel and modified-release dipyridamole for the prevention of occlusive vascular events (review of Technology Appraisal No. 90): a systematic review and economic analysis. *Health Technol Assess* 2011;15(31):1-178.
9. Davies L, Brown TJ, Haynes S, et al. Cost-effectiveness of cell salvage and alternative methods of minimising perioperative allogeneic blood transfusion: a systematic review and economic model. *Health Technol Assess* 2006;10(44).
10. Palmer S, Sculpher M, Philips Z, et al. A cost-effectiveness model comparing alternative management strategies for the use of glycoprotein IIb/IIIa antagonists in non-st-elevation acute coronary syndrome. Report to the National Institute for Clinical Excellence 2002.
11. Pasupathy S, Air T, Dreyer RP, et al. Systematic review of patients presenting with suspected myocardial infarction and nonobstructive coronary arteries. *Circulation* 2015;131(10):861-70.
12. Kang WY, Jeong MH, Ahn YK, et al. Are patients with angiographically near-normal coronary arteries who present as acute myocardial infarction actually safe? *Int J Cardiol* 2011;146(2):207-12.
13. Pathik B, Raman B, Mohd Amin NH, et al. Troponin-positive chest pain with unobstructed coronary arteries: incremental diagnostic value of cardiovascular magnetic resonance imaging. *Eur Heart J Cardiovasc Imaging* 2016;17(10):1146-52.
14. Lindahl B, Baron T, Erlinge D, et al. Medical Therapy for Secondary Prevention and Long-Term Outcome in Patients With Myocardial Infarction With Nonobstructive Coronary Artery Disease. *Circulation* 2017;135(16):1481-89.
15. Dastidar AG, Rodrigues JCL, Johnson TW, et al. Myocardial Infarction With Nonobstructed Coronary Arteries: Impact of CMR Early After Presentation. *JACC Cardiovasc Imaging* 2017;10(10 Pt A):1204-06.

For peer review only - http://bmjopen.bmj.com/site/about/guidelines.xhtml

CHEERS Checklist

Items to include when reporting economic evaluations of health interventions

The **ISPOR CHEERS Task Force Report**, *Consolidated Health Economic Evaluation Reporting Standards (CHEERS)—Explanation and Elaboration: A Report of the ISPOR Health Economic Evaluations Publication Guidelines Good Reporting Practices Task Force,* provides examples and further discussion of the 24-item CHEERS Checklist and the CHEERS Statement. It may be accessed via the *Value in Health* or via the ISPOR Health Economic Evaluation Publication Guidelines – CHEERS: Good Reporting Practices webpage: http://www.ispor.org/TaskForces/EconomicPubGuidelines.asp

Section/item	Item No	Recommendation	Reported on page No/ line No
**Title and Abstract**			
Title	1	Identify the study as an economic evaluation or use more specific terms such as "cost-effectiveness analysis", and describe the interventions compared.	Title on page 1 uses the term 'cost-effectiveness analysis'. The intervention of interest, CMR imaging, is included in the title, but the comparators are not, since these differ in the two economic models, and there is not room to include both in the title.
Abstract	2	Provide a structured summary of objectives, perspective, setting, methods (including study design and inputs), results (including base case and uncertainty analyses), and conclusions.	Page 2.
**Introduction**			
Background and objectives	3	Provide an explicit statement of the broader context for the study. Present the study question and its relevance for health policy or practice decisions.	Introduction, pages 4-5. Introduction, pages 4-5; Methods: Overview and treatment strategies, final paragraph.
**Methods**			
Target population and subgroups	4	Describe characteristics of the base case population and subgroups analysed, including why they were chosen.	Introduction paragraphs 1 and 2, page 4; Methods: Overview and treatment strategies, page 5. No subgroups within these two populations considered.
Setting and location	5	State relevant aspects of the system(s) in which the decision(s) need(s) to be made.	Introduction paragraphs 1-3, page 4; Methods: Overview and treatment strategies, and Model structure, pages 5-6.

For peer review only

Study perspective	6	Describe the perspective of the study and relate this to the costs being evaluated.	Methods: Overview and treatment strategies, final sentence of section, page 5; and Methods: Cost parameter section, page 9 together with Appendices A&B in the Supplementary Information.
Comparators	7	Describe the interventions or strategies being compared and state why they were chosen.	Introduction paragraphs 2-3, page 4; Methods: Overview and treatment strategies, page 5.
Time horizon	8	State the time horizon(s) over which costs and consequences are being evaluated and say why appropriate.	Methods: Overview and treatment strategies, line 3 of section, page 5.
Discount rate	9	Report the choice of discount rate(s) used for costs and outcomes and say why appropriate.	Methods: Economic analyses, lines 3-4 of section, page 7.
Choice of health outcomes	10	Describe what outcomes were used as the measure(s) of benefit in the evaluation and their relevance for the type of analysis performed.	Methods: Overview and treatment strategies, first sentence, page 5.
Measurement of effectiveness	11a	*Single study-based estimates:* Describe fully the design features of the single effectiveness study and why the single study was a sufficient source of clinical effectiveness data.	N/A
	11b	*Synthesis-based estimates:* Describe fully the methods used for identification of included studies and synthesis of clinical effectiveness data.	Methods: Identification of model parameters, paragraph 1, final sentence, page 7; Methods: Quality-adjusted life years, pages 9-10.
Measurement and valuation of preference based outcomes	12	If applicable, describe the population and methods used to elicit preferences for outcomes.	Methods: Quality-adjusted life years, lines 4-6 of section, page 9.
Estimating resources and costs	13a	*Single study-based economic evaluation:* Describe approaches used to estimate resource use associated with the alternative interventions. Describe primary or secondary research methods for valuing each resource item in terms	N/A

For peer review only

		of its unit cost. Describe any adjustments made to approximate to opportunity costs.	
	13b	*Model-based economic evaluation:* Describe approaches and data sources used to estimate resource use associated with model health states. Describe primary or secondary research methods for valuing each resource item in terms of its unit cost. Describe any adjustments made to approximate to opportunity costs.	Methods: Identification of model parameters, lines 2-3, page 7; Methods: Cost parameters, page 9, together with Appendices A&B in the Supplementary Information.
Currency, price date, and conversion	14	Report the dates of the estimated resource quantities and unit costs. Describe methods for adjusting estimated unit costs to the year of reported costs if necessary. Describe methods for converting costs into a common currency base and the exchange rate.	Methods: Economic analyses, line 2 of section, page 7. Appendices A&B in the Supplementary Information.
Choice of model	15	Describe and give reasons for the specific type of decision analytical model used. Providing a figure to show model structure is strongly recommended.	Methods: Overview and treatment strategies, and Model structure, pages 5-6; Figures 1 and 2.
Assumptions	16	Describe all structural or other assumptions underpinning the decision-analytical model.	Methods, pages 5-10; Appendices A&B in the Supplementary Information.
Analytical methods	17	Describe all analytical methods supporting the evaluation. This could include methods for dealing with skewed, missing, or censored data; extrapolation methods; methods for pooling data; approaches to validate or make adjustments (such as half cycle corrections) to a model; and methods for handling population heterogeneity and uncertainty.	Methods: Economic analyses, page 7; Methods: Base-case and sensitivity analyses, page 10; Appendices A&B in the Supplementary Information.
**Results**			
Study parameters	18	Report the values, ranges, references, and, if used, probability distributions for all parameters. Report reasons or sources for distributions used to represent uncertainty where appropriate.<br>Providing a table to show the input values is strongly recommended.	Methods: Clinical parameters, pages 8-9; Methods: Base-case and sensitivity analyses, final paragraph of section, page 10; Appendices A&B in the Supplementary Information.

For peer review only

Incremental costs and outcomes	19	For each intervention, report mean values for the main categories of estimated costs and outcomes of interest, as well as mean differences between the comparator groups. If applicable, report incremental cost-effectiveness ratios.	Table 1, base case row, page 12; Table 2, base case row, page 15.
Characterising uncertainty	20a	*Single study-based economic evaluation:* Describe the effects of sampling uncertainty for the estimated incremental cost and incremental effectiveness parameters, together with the impact of methodological assumptions (such as discount rate, study perspective).	N/A
	20b	*Model-based economic evaluation:* Describe the effects on the results of uncertainty for all input parameters, and uncertainty related to the structure of the model and assumptions.	Results: Sensitivity analyses, pages 17-18; Tables 1 and 2; Appendices C&D in the Supplementary Information.
Characterising heterogeneity	21	If applicable, report differences in costs, outcomes, or cost-effectiveness that can be explained by variations between subgroups of patients with different baseline characteristics or other observed variability in effects that are not reducible by more information.	N/A
**Discussion**			
Study findings, limitations, generalisability, and current knowledge	22	Summarise key study findings and describe how they support the conclusions reached. Discuss limitations and the generalisability of the findings and how the findings fit with current knowledge.	Discussion, pages 18-20.
**Other**			
Source of funding	23	Describe how the study was funded and the role of the funder in the identification, design, conduct, and reporting of the analysis. Describe other non-monetary sources of support.	Funding section, pages 21-22.
Conflicts of interest	24	Describe any potential for conflict of interest of study contributors in accordance with journal policy. In	Competing interests section, page 22.

For peer review only

	the absence of a journal policy, we recommend authors comply with International Committee of Medical Journal Editors recommendations.

For peer review only

BMJ Open

Cardiovascular magnetic resonance in emergency patients with multi-vessel disease or unobstructed coronary arteries: a cost-effectiveness analysis in the United Kingdom

Journal:	*BMJ Open*
Manuscript ID	bmjopen-2018-025700.R1
Article Type:	Research
Date Submitted by the Author:	20-Mar-2019

Complete List of Authors:	Stokes, Elizabeth; University of Oxford, Health Economics Research Centre, Nuffield Department of Population Health<br>Doble, Brett; University of Oxford, Nuffield Department of Population Health<br>Pufulete, Maria; University of Bristol, Clinical Trials and Evaluation Unit; University Hospitals Bristol NHS Foundation Trust and University of Bristol, NIHR Bristol Biomedical Research Centre<br>Reeves, Barnaby; University of Bristol, Clinical Trials and Evaluation Unit; University Hospitals Bristol NHS Foundation Trust and University of Bristol, NIHR Bristol Biomedical Research Centre<br>Bucciarelli Ducci, Chiara; University Hospitals Bristol NHS Foundation Trust and University of Bristol, NIHR Bristol Biomedical Research Centre; University Hospitals Bristol NHS Foundation Trust, Bristol Heart Institute<br>Dorman, Stephen; University Hospitals Bristol NHS Foundation Trust, Bristol Heart Institute<br>Greenwood, John; University of Leeds, Leeds Institute of Cardiovascular and Metabolic Medicine<br>Anderson, Richard; University Hospital of Wales, Cardiology<br>Wordsworth, Sarah; University of Oxford, Health Economics Research Centre, Nuffield Department of Population Health

**Primary Subject Heading**:	Cardiovascular medicine
Secondary Subject Heading:	Health economics
Keywords:	Cardiovascular disease, Cardiovascular magnetic resonance, Cost-utility analysis, Diagnostic accuracy, Economic evaluation, Myocardial infarction < CARDIOLOGY

SCHOLARONE™
Manuscripts

Cardiovascular magnetic resonance in emergency patients with multi-vessel disease or unobstructed coronary arteries: a cost-effectiveness analysis in the United Kingdom

Elizabeth A. Stokes[1*], Brett Doble[1], Maria Pufulete[2,3], Barnaby C. Reeves[2,3], Chiara Bucciarelli-Ducci[3,4], Stephen Dorman[4], John P Greenwood[5], Richard A Anderson[6], Sarah Wordsworth[1]

Affiliations

[1]Health Economics Research Centre, Nuffield Department of Population Health, University of Oxford, Oxford, United Kingdom

[2]Clinical Trials and Evaluation Unit, Bristol Trials Centre, Bristol Medical School, University of Bristol, Bristol, United Kingdom

[3]NIHR Bristol Biomedical Research Centre, University Hospitals Bristol NHS Foundation Trust and University of Bristol, Bristol, United Kingdom

[4]Bristol Heart Institute, University Hospitals Bristol NHS Foundation Trust, Bristol, United Kingdom

[5]Leeds Institute of Cardiovascular and Metabolic Medicine, University of Leeds, Leeds, United Kingdom

[6]University Hospitals of Wales, Heath Park, Cardiff, United Kingdom

*Corresponding author: Elizabeth Stokes, Health Economics Research Centre, Nuffield Department of Population Health, University of Oxford, Old Road Campus, Headington, Oxford, OX3 7LF, United Kingdom. Tel: +44(0)1865 289276; Email: elizabeth.stokes@dph.ox.ac.uk

Word Count: 4254

For peer review only - http://bmjopen.bmj.com/site/about/guidelines.xhtml

ABSTRACT

Objective: To identify the key drivers of cost-effectiveness for cardiovascular magnetic resonance (CMR) when patients activate the primary percutaneous coronary intervention (PPCI) pathway.

Design: Economic decision models for two patient subgroups populated from secondary sources, each with a one-year time horizon from the perspective of the National Health Service (NHS) and personal social services in the UK.

Setting: Usual care (with or without CMR) in the NHS.

Participants: Patients who activated the PPCI pathway, and for Model 1: underwent an emergency coronary angiogram and PPCI, and were found to have multi-vessel coronary artery disease. For Model 2: underwent an emergency coronary angiogram, and were found to have unobstructed coronary arteries.

Interventions: Model 1 (multi-vessel disease) compared two different ischaemia testing methods, CMR or fractional flow reserve (FFR), versus stress echocardiography. Model 2 (unobstructed arteries) compared CMR with standard echocardiography versus standard echocardiography alone.

Main outcome measures: Key drivers of cost-effectiveness for CMR, incremental costs and quality-adjusted life years (QALYs) and incremental cost-effectiveness ratios.

Results: In both models the incremental costs and QALYs between CMR (or FFR, model 1) versus no CMR (stress echocardiography, model 1 and standard echocardiography, model 2) were small (CMR: -£64 (95% CI -£232, £187) / FFR: £360 (95% CI -£116, £844) and CMR/FFR: 0.0012 QALYs, (95% CI -0.0076, 0.0093)) and (£98, (95% CI -£199, £488) and 0.0005 QALYs (95% CI -0.0050, 0.0077)) respectively. The diagnostic accuracy of the tests was the key driver of cost-effectiveness for both patient groups.

Conclusions: If CMR were introduced for all subgroups of patients who activate the PPCI pathway, it is likely that diagnostic accuracy would be a key determinant of its cost-effectiveness. Further research is needed to definitively answer whether revascularisation guided by CMR or FFR leads to different clinical outcomes in acute coronary syndrome patients with multi-vessel disease.

Keywords: cardiovascular disease, cardiovascular magnetic resonance (CMR), cost-utility analysis, diagnostic accuracy, economic evaluation, myocardial infarction

For peer review only

ARTICLE SUMMARY

Strengths and limitations of this study

- We present the first cost-effectiveness analysis of cardiovascular magnetic resonance (CMR) and fractional flow reserve (FFR) ischaemia tests compared to stress echocardiography in patients who activate the primary percutaneous coronary intervention (PPCI) pathway and are found to have multi-vessel coronary disease, and the first cost-effectiveness analysis of introducing CMR in patients who activate the PPCI pathway and are found to have unobstructed coronary arteries.

- This work draws on all available evidence in this field to provide guidance on the key drivers of cost-effectiveness for future research.

- The majority of model parameter estimates were based on single studies with small sample sizes, conducted outside the UK where patient pathways differ.

- CMR and FFR tests were treated as reference standards (assumed 100% sensitivity and specificity) in base-case analyses; sensitivity analyses show that relative cost-effectiveness varies substantially depending on assumptions about these parameters.

For peer review only

INTRODUCTION

Patients with acute coronary syndrome (ACS) and suspected acute thrombotic coronary occlusion activate the primary percutaneous coronary intervention (PPCI) pathway. In the UK National Health Service (NHS), PPCI is the main clinical approach to restore blood flow in the infarcted artery after ST-elevation myocardial infarction (STEMI).[1] Many patients presenting to hospital with STEMI have multi-vessel disease (40-65%),[2-7] which may require secondary revascularisation of the patients' non-infarcted territories. In addition, 3-16% of patients who activate the PPCI pathway do not receive PPCI because angiography sometimes shows that the coronary arteries are unobstructed.[8, 9] These factors result in considerable uncertainty and sometimes a lack of a definitive diagnosis of myocardial infarction (MI) in these patients, which can lead to inappropriate clinical management,[10, 11] which may in turn be associated with poor prognosis or unnecessary resource use.[12]

Cardiovascular magnetic resonance (CMR) is a non-invasive imaging technique that assesses heart structure with high temporal resolution, and can help inform management decisions for ACS patients who activate the PPCI pathway. CMR can facilitate differential diagnosis in the context of a normal coronary angiogram,[13-15] providing a definitive diagnosis in 65-90% of patients.[13, 16, 17] The benefits of CMR in other subgroups of PPCI patients, such as those found to have multi-vessel disease, are less clear. Long-term outcome data and studies reporting the cost-effectiveness of CMR are absent, further complicating resource allocation of CMR services within the NHS. Despite a lack of evidence, CMR has been increasingly used in ACS patients in the UK,[18] and is included in the 2017 European Society of Cardiology guidelines on the management of patients presenting with STEMI.[19]

There has also been a rapid increase in the use of fractional flow reserve (FFR) testing; a guide wire-based procedure which is undertaken through a standard guide catheter during invasive angiography to assess the degree of ischaemia in stenosed coronary arteries. Stress CMR is considered to have excellent diagnostic accuracy compared to FFR for detecting ischaemia.[20, 21] The main benefit of ischaemia testing using FFR is that lesions can be assessed and revascularised in the same session; however if revascularisation is not required, then the benefit of FFR over CMR is less clear. In addition to CMR and FFR, stress echocardiography (ECHO) and myocardial perfusion scintigraphy using single photon emission computed tomography (MPS-SPECT) are often used to evaluate patients with multi-vessel disease for residual ischaemia of bystander disease after PPCI for STEMI; stress ECHO is one of the most commonly used imaging modalities in the UK.[22]

For peer review only - http://bmjopen.bmj.com/site/about/guidelines.xhtml

In this paper, we present a health economic analysis based on a study that was designed to establish the feasibility of setting up a UK multi-centre registry to document CMR use in patients who activate the PPCI pathway and determine its prognostic value and impact on patient management.[23] Here we present the results of two economic decision models which we designed to compare the cost-effectiveness of introducing CMR in two patient subgroups: patients with multi-vessel disease and those with unobstructed coronary arteries. The aim was to identify how the results of the analyses change in response to sensitivity analyses to determine key drivers of cost-effectiveness that could be considered for detailed measurement in any future research.

METHODS

Overview and treatment strategies

We built two separate cost-effectiveness models in consultation with clinical experts to estimate the healthcare costs and quality-adjusted life years (QALYs) of introducing CMR after PPCI pathway activation in two patient subgroups over a one-year time horizon:

1) Model 1 compares two types of ischaemia testing: CMR or FFR to stress ECHO in patients with multi-vessel disease (commonly defined as stenosis >50%) after index angiogram and PPCI;
2) Model 2 compares CMR in addition to standard ECHO to standard ECHO alone in patients with unobstructed coronary arteries after index angiogram.

Given the focus on the immediate consequences on clinical management of introducing additional diagnostic testing, and because this is a feasibility study assessing key drivers of cost-effectiveness rather than cost-effectiveness per se, we chose a one-year time horizon. Survival to 12 months under each testing strategy was assessed to determine whether there were differences, which would identify a need to consider a longer time horizon. From these two models, it may be possible to make inferences about the likely drivers of cost-effectiveness of introducing CMR for all subgroups of patients who activate the PPCI pathway. The analysis was conducted from an NHS and personal social services perspective in the UK.[24]

For peer review only - http://bmjopen.bmj.com/site/about/guidelines.xhtml

Model structure

Model 1: multi-vessel disease

Model 1 uses a decision tree structure (Figure 1) to compare the cost-effectiveness of two types of ischaemia testing (CMR and FFR) to stress ECHO. All three tests are used to guide decisions regarding secondary revascularisation in patients with multi-vessel disease. Based on the findings of their emergency index angiogram at which the culprit lesion is treated, patients can enter one of three test/treatment pathways to determine whether further treatment is required for non-culprit lesions at a later date: no ischaemia testing and secondary revascularisation; ischaemia testing to guide decisions for secondary revascularisation; or no ischaemia testing and no secondary revascularisation. Patients are then divided into whether they 'truly' have ischaemia or not. For patients receiving ischaemia testing, the test results can be either positive or negative (ischaemia present or not), and the model reflects potential misclassification of patients (i.e., ischaemia testing has a sensitivity and specificity). Patients entering any of the three test/treatment pathways are then at risk of experiencing a major adverse cardiovascular event (MACE) or no MACE over the one-year time horizon. MACE is a composite endpoint of all-cause mortality, MI, stroke or (repeat) revascularisation.

The model structure is the same for the three ischaemia testing options, but there are differences in the timing of testing and secondary revascularisation between these arms (note for all arms this testing and secondary revascularisation, if required, is at a later date than the index angiogram at PPCI and treatment of the culprit lesion). For CMR and stress ECHO, this is a two-step process (i.e., a patient has the ischaemia test, and then separately has secondary revascularisation). For FFR, this happens at the same time (i.e., a patient has their diagnostic angiogram and FFR, and if required, secondary revascularisation). We have assumed that this timing difference only affects costs, but not the model structure.

Model 2: unobstructed coronary arteries

Model 2 also uses a decision tree structure (Figure 2) to compare the cost-effectiveness of CMR with standard ECHO to 'current practice' of standard ECHO only. These tests are used to guide decisions in offering treatment for MI in patients who activate the PPCI pathway but are found to have unobstructed coronary arteries. In each arm, patients are divided according to whether they truly had an MI or not. Results for both testing approaches can be either positive or negative (MI occurred or not), with the model again reflecting the potential for misclassification. Patients receiving a positive test result are

For peer review only - http://bmjopen.bmj.com/site/about/guidelines.xhtml

assumed to have had an MI and receive treatment (cardiac rehabilitation and medications). A negative test results in treatment for other cardiac causes of chest pain (fewer cardiac medications or none at all). Thereafter, patients are divided into whether they had MACE or not over the one-year time horizon.

Economic analyses

TreeAge Pro 2013 (TreeAge Software Inc., Williamstown, MA) was used to develop and analyse the models. Resources were valued in 2015/16 pounds sterling. The expected costs and QALYs associated with each testing strategy to one-year were calculated. Costs and QALYs were not discounted, as the time horizon was only one-year. Incremental cost-effectiveness ratios (ICERs) were calculated as the ratio of the difference in cost to the difference in QALYs between each testing option and its comparator.

Identification of model parameters

Testing and clinical parameters used in both models were derived from reviews of the literature. Unit costs were obtained from standard UK sources, notably the National Schedule of Reference Costs and the British National Formulary.[25, 26] Health state utility values were identifed by searching the Tufts Cost-effectiveness Analysis (CEA) Registry.[27]

For Model 1, we searched Medline using Medical Subject Headings and keywords for each ischaemia test (e.g. exp/pressure-wire or pressure-wire.mp or fractional flow reserve.mp and exp/myocardial infarction). Review articles, reference lists and citations of relevant studies were also evaluated. For Model 2, initial Medline searching identified a 2016 review paper,[28] which referenced the inaugural study of CMR in patients with unobstructed coronary arteries.[13] All citations of this primary paper were reviewed.[13] Two meta-analyses were identified and their citations were reviewed.[29, 30] Medline was also searched using keywords such as ‘myocardial infarction with non-obstructive coronary arteries’ and synonyms.

Complete lists of model parameters, their respective sources and assumptions made in their estimation are provided in Appendix A and B and briefly summarised below.

Testing parameters

Model 1: multi-vessel disease

CMR and FFR were treated as reference standards with assumed 100% sensitivity and specificity. While FFR is the reference standard for detecting ischaemia in patients with

For peer review only - http://bmjopen.bmj.com/site/about/guidelines.xhtml

stable coronary artery disease, there is uncertainty in how FFR-guided revascularisation influences outcome in this high-risk group of STEMI patients with multi-vessel disease. It was therefore considered a reasonable base-case assumption that the sensitivities and specificities of CMR and FFR were the same in this population. While in practice their sensitivity and specificity are likely to be a little less than 100%, there is no gold standard test for ischaemia in STEMI patients with multi-vessel disease with which to compare; lower sensitivities and specificities were explored in sensitivity analyses. The assumption of 100% sensitivity and specificity for CMR and FFR results in identical probabilities and outcomes, but differences in costs for the two arms. Sensitivity and specificity values for stress ECHO were obtained from Gurunathan et al.[31]

Model 2: unobstructed coronary arteries

CMR with standard ECHO was treated as a reference standard, whereas sensitivity and specificity values for standard ECHO were obtained from Dastidar et al.[32]

Clinical parameters

Model 1: multi-vessel disease

The probabilities of patients entering one of the three test/treatment pathways were based on expert clinical opinion. Probabilities of true ischaemia for each test/treatment pathway were derived from the FAME study.[33] Subgroup outcomes from Smits et al. were used to estimate probabilities of MACE for three patient groups: truly ischaemic patients who had secondary revascularisation, truly ischaemic patients who did not have secondary revascularisation, and truly non-ischaemic patients, regardless of secondary revascularisation.[34]

Model 2: unobstructed coronary arteries

Studies reporting the probability of MACE in patients with unobstructed coronary arteries have used varying definitions of MACE and follow-up periods (12-34 months).[35-38] In patients with <50% coronary artery stenosis, Kang et al. reported a 7.8% risk of MACE at one-year,[37] but their MACE definition did not include stroke. Three studies reported MACE, broken down by CMR diagnosis.[35, 36, 38] Pathik reported MACE for 27% of patients diagnosed with MI, and for 5% of patients without MI (diagnosed with myocarditis, cardiomyopathy or normal CMR) over a median of 24 months.[38] In the other two studies, no MACE outcomes were observed in the non-MI patients. The MACE estimates reported in Pathik et al. were converted to continuous rates (assuming events occur evenly over 24 months of follow-up). The ratio of events in the MI patients compared to the

For peer review only - http://bmjopen.bmj.com/site/about/guidelines.xhtml

non-MI patients was six, and was used with the overall estimate of MACE at one-year from Kang et al. (0.078),[37] and the probability of patients truly having an MI from Pasupathy et al. (0.24),[29] to calculate the probability of MACE in patients who truly had an MI (0.21) and in those who did not have an MI (0.03).

The hazard ratio for MACE in patients who were taking statins compared to those who were not (0.77) reported by Lindahl et al. was used to adjust the probability of MACE for patients who had an MI, giving a probability of MACE for those with MI but without treatment of 0.26.[39] For patients without MI, the probability of MACE of 0.03 was assumed to apply regardless of treatment.

Cost parameters

Model 1: multi-vessel disease

The costs considered for all three ischaemia testing strategies included: the cost of ischaemia testing (CMR, FFR, or stress ECHO) if required, secondary revascularisation, adverse events included in MACE (initial inpatient and post-discharge costs), medications and cardiac rehabilitation offered to all patients and additional healthcare costs beyond hospital discharge to one-year, including follow-up outpatient appointments.

Model 2: unobstructed coronary arteries

The costs considered for the two testing strategies included: the cost of testing (CMR and/or standard ECHO), cardiac rehabilitation and medications for patients classified as either having had an MI or a non-MI diagnosis (details in Appendix B), adverse events included in MACE (initial inpatient and post-discharge costs) and additional healthcare costs beyond hospital discharge to one-year, including a follow-up appointment.

Quality-adjusted life years

Model 1: multi-vessel disease

QALYs were estimated over one-year as a function of survival and health state utility values. Since death is one of the components of MACE, we assumed that patients without MACE are all alive at one-year and that the probability of dying for MACE patients was 0.08 as estimated by Smits et al.[34] We identified mean utility values of 0.72, 0.77 and 0.77 at baseline (during index hospitalisation), one and twelve-months respectively estimated using the UK EQ-5D-3L tariff in patients with STEMI having PPCI.[27, 40]

For peer review only - http://bmjopen.bmj.com/site/about/guidelines.xhtml

QALYs were calculated assuming a patient's utility changed linearly between each time point or death. Patients who died were assumed to die midway through the time-period (six months), and have a utility of zero after death. QALYs for patients without MACE who were all alive at one-year were calculated based on the utility values for the three time-points above.[40] QALYs for patients with MACE were also calculated using these three utility values, but assuming 8% of patients died to produce a weighted average for patients alive and dead at one-year.[34] A utility decrement of 0.05 was applied to this weighted average to reflect the reduced quality-of-life of patients with MACE compared to those without.[41] QALYs for patients who had secondary revascularisation were modified to assume their utility at the one-month time-point was repeated for a subsequent month.

Model 2: unobstructed coronary arteries

Searches of the CEA Registry did not yield any utility values for patients with unobstructed coronary arteries, and therefore QALYs for patients with and without MACE in Model 2 were calculated using the same utility values and methods as for Model 1.[40]

Base-case and sensitivity analyses

The best available point estimates were used in the base-case analyses in which ICERs were the summary measures. Since this was a feasibility study, the actual ICERs are not the main interest, but rather how they change in response to sensitivity analyses.

One-way deterministic sensitivity analyses (DSAs) were conducted to test the impact of varying the base-case values for several model parameters. Alternative values for DSAs (Appendix C and D) were obtained from substitute evidence sources, clinical judgment (e.g. 8% probability of death for patients with MACE was thought to be low, and a relative risk of 6.0 to be high for events in MI compared to non-MI patients, so higher and lower values were explored) or altering mean values for unit costs by ±20%. Threshold analyses were also conducted to establish the cost of each test at which the expected costs associated with an alternative strategy would be identical.

Probabilistic sensitivity analyses (PSAs) were used to account for the impact on results of all uncertain parameters simultaneously. Parameters were assigned distributions based on the precision of estimates available to describe a range of plausible values, and 1,000 randomly selected values from each distribution were generated and results calculated for each run of the model. 95% confidence intervals and scatter plots of the 1,000 incremental costs and QALYs for each comparison were then assessed.

For peer review only - http://bmjopen.bmj.com/site/about/guidelines.xhtml

Patient and public involvement

Patients and the public were not involved in the design of this study.

RESULTS

Base-case results

Model 1: multi-vessel disease

Over one-year, the expected total costs of stress ECHO, CMR and FFR strategies per patient were £5495, £5431 and £5855 respectively (Table 1). Use of stress ECHO produced 0.7564 QALYs, whereas CMR and FFR both resulted in an additional 0.0012 QALYs gained. CMR dominated stress ECHO as a CMR strategy was less costly (-£64) and more effective (+0.0012 QALYs). FFR was more costly (£360) and more effective (+0.0012 QALYs) than stress ECHO, resulting in an ICER above the accepted cost-effectiveness threshold of £20,000-£30,000 per QALY.

For peer review only - http://bmjopen.bmj.com/site/about/guidelines.xhtml

Table 1. Base-case and deterministic one-way sensitivity analyses results for Model 1 (multi-vessel disease)

DSA	Ischaemia testing option	Costs (£) Mean (SE)	QALYs Mean (SE)	Difference in costs to Stress ECHO Mean (95% CI)	Difference in QALYs to Stress ECHO Mean (95% CI)	ICER (£) – compared to Stress ECHO
Base	Stress ECHO	5495 (556)	0.7564 (0.0545)			
Case	CMR	5431 (560)	0.7576 (0.0551)	-64 (-232, 187)	0.0012 (-0.0076, 0.0093)	CMR dominant (-53,563)
	FFR	5855 (539)	0.7576 (0.0551)	360 (-116, 844)	0.0012 (-0.0076, 0.0093)	300,216
1 Decision	Stress ECHO	4575 (449)	0.7537 (0.0654)			
From	CMR	4558 (500)	0.7540 (0.0661)	-17 (-62, 45)	0.0003 (-0.0022, 0.0026)	CMR dominant (-53,563)
angiogram	FFR	4667 (500)	0.7540 (0.0661)	93 (-21, 214)	0.0003 (-0.0022, 0.0026)	300,216
2a CMR test	Stress ECHO	5495 (556)	0.7564 (0.0545)			
sensitivity	CMR	5349 (549)	0.7569 (0.0553)	-146 (-362, 105)	0.0005 (-0.0162, 0.0181)	CMR dominant (-276,922)
2b CMR test	Stress ECHO	5495 (556)	0.7564 (0.0545)			
specificity	CMR	5639 (593)	0.7574 (0.0554)	145 (-103, 524)	0.0010 (-0.0155, 0.0192)	138,608
2c CMR test	Stress ECHO	5495 (556)	0.7564 (0.0545)			
accuracy	CMR	5558 (600)	0.7567 (0.0572)	63 (-202, 390)	0.0004 (-0.0119, 0.0108)	168,453
3a FFR test	Stress ECHO	5495 (556)	0.7564 (0.0545)			
sensitivity	FFR	5817 (555)	0.7569 (0.0564)	322 (-138, 782)	0.0005 (-0.0162, 0.0181)	610,864
3b FFR test	Stress ECHO	5495 (556)	0.7564 (0.0545)			
specificity	FFR	5982 (567)	0.7574 (0.0572)	487 (13, 1024)	0.0010 (-0.0155, 0.0192)	466,754
3c FFR test	Stress ECHO	5495 (556)	0.7564 (0.0545)			
accuracy	FFR	5944 (568)	0.7567 (0.0572)	449 (-18, 947)	0.0004 (-0.0119, 0.0108)	1,207,875

For peer review only - http://bmjopen.bmj.com/site/about/guidelines.xhtml

DSA	Ischaemia testing option	Costs (£) Mean (SE)	QALYs Mean (SE)	Difference in costs to Stress ECHO Mean (95% CI)	Difference in QALYs to Stress ECHO Mean (95% CI)	ICER (£) – compared to Stress ECHO
4a ECHO	Stress ECHO	5534 (552)	0.7567 (0.0562)			
test	CMR	5431 (560)	0.7576 (0.0551)	-103 (-274, 124)	0.0009 (-0.0122, 0.0114)	CMR dominant (-117,499)
sensitivity	FFR	5855 (539)	0.7576 (0.0551)	321 (-157, 761)	0.0009 (-0.0122, 0.0114)	365,070
4b ECHO	Stress ECHO	5392 (565)	0.7564 (0.0562)			
test	CMR	5431 (560)	0.7576 (0.0551)	39 (-105, 294)	0.0011 (-0.0035, 0.0067)	34,961
specificity	FFR	5855 (539)	0.7576 (0.0551)	463 (54, 935)	0.0011 (-0.0035, 0.0067)	413,065
4c ECHO	Stress ECHO	5431 (553)	0.7568 (0.0555)			
test	CMR	5431 (560)	0.7576 (0.0551)	+0 (-128, 253)	0.0008 (-0.0022, 0.0036)	201
accuracy	FFR	5855 (539)	0.7576 (0.0551)	424 (18, 888)	0.0008 (-0.0022, 0.0036)	529,193
5	Stress ECHO	5674 (586)	0.7520 (0.0566)			
MACE	CMR	5610 (580)	0.7533 (0.0574)	-65 (-233, 164)	0.0012 (-0.0078, 0.0087)	CMR dominant (-53,434)
+0.05	FFR	6033 (575)	0.7533 (0.0574)	359 (-85, 831)	0.0012 (-0.0078, 0.0087)	296,587
5	Stress ECHO	5317 (564)	0.7607 (0.0589)			
MACE	CMR	5252 (565)	0.7619 (0.0595)	-65 (-244, 192)	0.0012 (-0.0081, 0.0097)	CMR dominant (-53,463)
-0.05	FFR	5676 (533)	0.7619 (0.0595)	359 (-112, 830)	0.0012 (-0.0081, 0.0097)	297,408
6 CMR cost	Stress ECHO	5495 (556)	0.7564 (0.0545)			
+20%	CMR	5463 (556)	0.7576 (0.0551)	-32 (-218, 187)	0.0012 (-0.0076, 0.0093)	CMR dominant (-27,021)
6 CMR cost	Stress ECHO	5495 (556)	0.7564 (0.0545)			
-20%	CMR	5399 (551)	0.7576 (0.0551)	-96 (-268, 159)	0.0012 (-0.0076, 0.0093)	CMR dominant (-80,104)

For peer review only - http://bmjopen.bmj.com/site/about/guidelines.xhtml

DSA	Ischaemia testing option	Costs (£) Mean (SE)	QALYs Mean (SE)	Difference in costs to Stress ECHO Mean (95% CI)	Difference in QALYs to Stress ECHO Mean (95% CI)	ICER (£) – compared to Stress ECHO
7 FFR cost +20%	Stress ECHO	5495 (556)	0.7564 (0.0545)			
	FFR	6015 (560)	0.7576 (0.0551)	521 (78, 992)	0.0012 (-0.0076, 0.0093)	434,425
7 FFR cost -20%	Stress ECHO	5495 (556)	0.7564 (0.0545)			
	FFR	5694 (537)	0.7576 (0.0551)	199 (-240, 622)	0.0012 (-0.0076, 0.0093)	166,007
8 QALYs for MACE -0.2	Stress ECHO	5495 (556)	0.7306 (0.0559)			
	CMR	5431 (560)	0.7346 (0.0565)	-64 (-232, 187)	0.0040 (-0.0057, 0.0117)	CMR dominant (-16,041)
	FFR	5855 (539)	0.7346 (0.0565)	360 (-116, 844)	0.0040 (-0.0057, 0.0117)	89,907
9 QALYs for no MACE +0.2	Stress ECHO	5495 (556)	0.9306 (0.0576)			
	CMR	5431 (560)	0.9346 (0.0587)	-64 (-232, 187)	0.0040 (-0.0071, 0.0104)	CMR dominant (-16,041)
	FFR	5855 (539)	0.9346 (0.0587)	360 (-116, 844)	0.0040 (-0.0071, 0.0104)	89,907

CMR, Cardiovascular Magnetic Resonance; CI, Confidence interval; ECHO, Echocardiography; FFR, Fractional flow reserve; ICER, Incremental cost-effectiveness ratio; MACE, Major adverse cardiovascular events; QALY, Quality-adjusted life year; SA, Sensitivity analysis; SE, Standard error

Note: all costs and ICERs are rounded to nearest pound

For peer review only

Model 2: unobstructed coronary arteries

Over one-year, the expected total costs of CMR with standard ECHO and standard ECHO alone strategies per patient were £3130 and £3032 respectively (Table 2). Use of CMR with standard ECHO produced 0.0005 more QALYs than standard ECHO alone, at an increase in costs of £98, resulting in an ICER above the accepted cost-effectiveness threshold.

For peer review only

For peer review only - http://bmjopen.bmj.com/site/about/guidelines.xhtml

Table 2. Base-case and deterministic one-way sensitivity analyses results for Model 2 (unobstructed coronary arteries)

DSA	Testing option	Costs (£) Mean (SE)	QALYs Mean (SE)	Difference in costs Mean (95% CI)	Difference in QALYs Mean (95% CI)	ICER (£)
Base	Standard ECHO	3032 (564)	0.7615 (0.0837)			
Case	Standard ECHO and CMR	3130 (589)	0.7620 (0.0844)	98 (-199, 488)	0.0005 (-0.0050, 0.0077)	190,114
1a CMR test	Standard ECHO	3032 (564)	0.7615 (0.0837)			
sensitivity	Standard ECHO and CMR	3121 (596)	0.7618 (0.0838)	89 (-163, 460)	0.0003 (-0.0030, 0.0048)	278,043
1b CMR test	Standard ECHO	3032 (564)	0.7615 (0.0837)			
specificity	Standard ECHO and CMR	3191 (612)	0.7620 (0.0840)	160 (-132, 567)	0.0005 (-0.0047, 0.0076)	309,400
1c CMR test	Standard ECHO	3032 (564)	0.7615 (0.0837)			
accuracy	Standard ECHO and CMR	3183 (591)	0.7618 (0.0850)	151 (-105, 523)	0.0003 (-0.0026, 0.0053)	469,998
2a ECHO test	Standard ECHO	3042 (569)	0.7617 (0.0835)			
sensitivity	Standard ECHO and CMR	3130 (589)	0.7620 (0.0844)	88 (-163, 479)	0.0003 (-0.0029, 0.0042)	299,955
2b ECHO test	Standard ECHO	2935 (539)	0.7615 (0.0824)			
specificity	Standard ECHO and CMR	3130 (589)	0.7620 (0.0844)	195 (-95, 600)	0.0005 (-0.0047, 0.0070)	378,523
2c ECHO test	Standard ECHO	2945 (561)	0.7617 (0.0863)			
accuracy	Standard ECHO and CMR	3130 (589)	0.7620 (0.0844)	185 (-59, 581)	0.0003 (-0.0027, 0.0042)	631,744
3 MACE	Standard ECHO	3067 (541)	0.7608 (0.0826)			
(ratio=4)	Standard ECHO and CMR	3165 (559)	0.7614 (0.0830)	98 (-192, 465)	0.0005 (-0.0043, 0.0069)	190,154
4 MACE	Standard ECHO	3043 (573)	0.7613 (0.0829)			
(ratio=2)	Standard ECHO and CMR	3146 (606)	0.7617 (0.0834)	104 (-162, 498)	0.0004 (-0.0051, 0.0063)	250,905
5 MACE	Standard ECHO	3046 (579)	0.7612 (0.0847)			
(ratio=1)	Standard ECHO and CMR	3161 (596)	0.7614 (0.0850)	115 (-168, 492)	0.0002 (-0.0051, 0.0069)	554,609

For peer review only - http://bmjopen.bmj.com/site/about/guidelines.xhtml

DSA	Testing option	Costs (£) Mean (SE)	QALYs Mean (SE)	Difference in costs Mean (95% CI)	Difference in QALYs Mean (95% CI)	ICER (£)
6 CMR cost	Standard ECHO	3032 (564)	0.7615 (0.0837)			
+20%	Standard ECHO and CMR	3183 (579)	0.7620 (0.0844)	151 (-133, 532)	0.0005 (-0.0050, 0.0077)	292,836
6 CMR cost	Standard ECHO	3032 (564)	0.7615 (0.0837)			
-20%	Standard ECHO and CMR	3077 (572)	0.7620 (0.0844)	45 (-229, 476)	0.0005 (-0.0050, 0.0077)	87,391
7 Reduce	Standard ECHO	2974 (548)	0.7615 (0.0837)			
MACE costs	Standard ECHO and CMR	3077 (574)	0.7620 (0.0844)	103 (-159, 502)	0.0005 (-0.0050, 0.0077)	198,992
8 QALYs for	Standard ECHO	3032 (564)	0.7457 (0.0879)			
MACE -0.2	Standard ECHO and CMR	3130 (589)	0.7474 (0.0886)	98 (-199, 488)	0.0018 (-0.0091, 0.0139)	55,281
9 QALYs for	Standard ECHO	3032 (564)	0.9457 (0.0905)			
no MACE +0.2	Standard ECHO and CMR	3130 (589)	0.9474 (0.0911)	98 (-199, 488)	0.0018 (-0.0094, 0.0134)	55,281
10 QALYs for	Standard ECHO	2994 (516)	0.7488 (0.0859)			
MACE (50% die)	Standard ECHO and CMR	3095 (542)	0.7503 (0.0864)	101 (-180, 464)	0.0015 (-0.0090, 0.0133)	66,040

CMR, Cardiovascular Magnetic Resonance; CI, Confidence interval; ECHO, Echocardiography; ICER, Incremental cost-effectiveness ratio; MACE, Major adverse cardiovascular events; QALY, Quality-adjusted life year; SA, Sensitivity analysis; SE, Standard error

Note: all costs and ICERs are rounded to nearest pound.

For peer review only

Sensitivity analyses

Model 1: multi-vessel disease

Table 1 reports the results of the DSAs for Model 1. DSA 1, varying the probabilities of a clinician ordering an ischaemia test and of a decision to perform a secondary revascularisation (or not), affects the mean costs and QALYs under each strategy but does not alter the cost-effectiveness results. When the specificity, or sensitivity and specificity of CMR were reduced to 80% (DSA 2), CMR became more costly than stress ECHO, rather than dominant, and at accepted cost-effectiveness thresholds, would not be considered cost-effective relative to stress ECHO. When the sensitivity and/or specificity of FFR were reduced to 80% (DSA 3), conclusions did not alter, but the ICER increased. In DSA 4, using higher sensitivity, or sensitivity and specificity values for stress ECHO, the ICER for CMR would still be considered cost-effective but when using a higher specificity only for stress ECHO, CMR would no longer be considered cost-effective. Improved sensitivity and/or specificity for stress ECHO resulted in an even more unfavourable ICER for FFR. Altering the probability of MACE (DSA 5), the costs of the ischaemia tests (DSAs 6 and 7), and QALYs for patients with and without MACE (DSAs 8 and 9), did not alter cost-effectiveness conclusions.

Threshold analyses identified that if the cost of CMR increased to £371 (from a base-case value of £264), there would be no difference in the expected costs of the CMR and stress ECHO strategies. Similarly, the cost of angiogram and FFR would need to be reduced to £250 and £415 (from a base-case value of £1,340) for there to be no difference in the expected costs of CMR and stress ECHO strategies respectively compared to FFR.

The 1,000 simulated incremental costs and QALYs from the PSA (Figures 3A and 3B) show considerable uncertainty in the base-case results. This uncertainty is apparent in both figures as the spread of estimates crosses two or three quadrants of the cost-effectiveness plane.

Under all testing strategies, the probability of survival to 12 months was 0.99. If a cohort of 1000 patients entered each strategy, on average there would be one more death by 12 months under a stress ECHO strategy compared to CMR/FFR (11 versus 10 deaths). Given that there is no difference in survival, we did not extrapolate findings to a longer time horizon.

For peer review only - http://bmjopen.bmj.com/site/about/guidelines.xhtml

Model 2: unobstructed coronary arteries

Table 2 reports the results of the DSAs for Model 2. Reducing the diagnostic accuracy of CMR (DSA 1), increasing the diagnostic accuracy of standard ECHO (DSA 2) and increasing the cost of CMR (DSA 6) had the greatest impact on the mean incremental cost between CMR with standard ECHO and standard ECHO alone. Altering QALYs (DSAs 8, 9 and 10), had the greatest impact on the mean incremental QALYs between the arms. However, in all DSAs conducted, CMR with standard ECHO remained both more costly and more effective than standard ECHO alone, resulting in ICERs that were consistently above the accepted cost-effectiveness threshold.

Threshold analyses show that if the cost of CMR reduced to £166 (from a base-case value of £264), there would be no difference in the expected costs between strategies.

Similar to Model 1, the 1,000 simulated incremental costs and QALYs (Figure 3C) indicate considerable uncertainty in the base-case results; again, the estimates are spread across three quadrants of the cost-effectiveness plane.

Under both strategies, the probability of survival to 12 months was 0.99. If a cohort of 1000 patients entered each strategy, on average there would be one more death by 12 months under a standard ECHO strategy compared to standard ECHO and CMR (seven versus six deaths). As for Model 1, given that there is no difference in survival, we did not extrapolate findings to a longer time horizon.

DISCUSSION

We developed economic decision models to identify key drivers of cost-effectiveness of CMR compared to 'current practice' in two subgroups of patients who activate the PPCI pathway: multi-vessel disease (Model 1) and unobstructed coronary arteries (Model 2). 'Current practice' for ischemia testing in multi-vessel disease varies widely. The results of both models suggest that differences in QALYs between strategies are small and, therefore, the results are largely driven by modest differences in costs.

In Model 1, only 35% of patients receiving ischaemia testing truly had ischaemia, therefore the majority of patients received an expensive test without needing further treatment. If more patients needed revascularisation the expected cost of the FFR strategy would reduce as potential economies of scale would result from performing the ischaemia test and secondary revascularisation concurrently. In Model 2, the reduction in costs

For peer review only - http://bmjopen.bmj.com/site/about/guidelines.xhtml

For peer review only

associated with treating fewer patients for MI if CMR were to be introduced only partially compensated for the additional cost of CMR. DSAs for both models identified the diagnostic accuracy of the tests as the key driver of cost-effectiveness. In Model 1, the costs of ischaemia testing and QALYs associated with MACE/ no MACE also influenced cost-effectiveness, but the latter factors had minimal impact compared to altering the diagnostic accuracy of CMR and FFR. For Model 2, the cost of CMR, the QALYs associated with MACE/ no MACE, and the proportion of patients with MACE who die, influenced cost-effectiveness results.

Overall, we have identified the sensitivity and specificity of the tests as the key drivers of cost-effectiveness in both models. It is likely that any business case to introduce CMR for all patients who activate the PPCI pathway would require accurate estimates of these parameters to be collected in future studies.

In terms of limitations of our study, first, many parameter estimates were obtained from a single study conducted outside the UK, where practices may differ. Estimates from different patient groups were sometimes used to populate the model. For example, in Model 1 the diagnostic accuracy of stress ECHO was derived from a study diagnosing significant coronary artery disease, rather than PPCI patients.[31] Despite these potential limitations, our model represents the best available evidence.

Second, for the base-case analysis of Model 1, CMR and FFR were treated as reference standards, assuming 100% sensitivity and specificity. We considered identical values for CMR and FFR as a reasonable starting point; while FFR is the reference standard for detecting ischaemia in patients with stable coronary artery disease, there is uncertainty in whether this also applies to a STEMI population with multi-vessel disease. There is no gold standard test for ischaemia in this population with which to compare. However, it is important to note that varying the sensitivity and specificity in DSAs for Model 1 had large impacts on cost-effectiveness results. Similarly, for Model 2, reducing the diagnostic accuracy of CMR with standard ECHO compared to standard ECHO doubled the ICER.

Third, resource use was estimated for patients in both models based on standard patient pathways described by clinical experts, as individual patient data from routine sources (e.g. Hospital Episode Statistics) were not available at the time of analysis. This may underestimate the variability between individual patients.

For peer review only - http://bmjopen.bmj.com/site/about/guidelines.xhtml

Finally, there was uncertainty around the utility decrement of 0.05 for patients with MACE compared to those without MACE.[41] Although this decrement has been used by others, it is not based on primary data.[42] In addition, utility estimates for patients with unobstructed coronary arteries were not available requiring the use of utility estimates from a population with STEMI having PPCI in Model 2. We considered these assumptions to be reasonable given (a) quality-of-life using the Short Form-36 was similar for patients with unobstructed coronary arteries and a control group of MI patients with coronary heart disease,[43] and (b) we varied QALYs in the DSAs.

Our cost-effectiveness models have highlighted that the diagnostic accuracies of the tests (CMR and FFR, and CMR) are key drivers of the relative cost-effectiveness of management strategies based on these tests when compared to 'current practice' for patients with multi-vessel disease and unobstructed coronaries respectively. In the base-case analyses for patients with multi-vessel disease, FFR was not cost-effective relative to stress ECHO, and was more costly than a CMR strategy. For patients with stable angina, the MR-INFORM trial showed that CMR-guided management is non-inferior for MACE at one-year compared with invasive angiography and FFR.[44] It is, therefore, concerning that there has been rapid adoption of FFR for detecting ischaemia during our study despite a lack of evidence of benefit over CMR; this may reflect the lack of capacity for CMR nationally. Future research should seek to quantify the relative diagnostic accuracy of CMR and FFR compared to stress ECHO, and versus each other. The fact that both CMR and FFR testing are regarded as reference standards in clinical practice and no superior standard is recognised may mean that the cost-effectiveness of CMR compared to FFR can only be tested in a randomised controlled trial, in which the impact of the ischaemia tests on patients' care pathways could be directly observed.

For peer review only

ACKNOWLEDGEMENTS

We would like to thank Alice Redfern, who assisted with the literature searches for parameter estimates for the models, and Danielle Bargo, who assisted with the design of the health economic model structures.

AUTHOR CONTRIBUTIONS

EAS designed the study, extracted, analysed and interpreted all the data, critically reviewed and edited the draft article and gave final approval of this version to be published.

BD assisted with the interpretation of the data, drafted and revised this article and gave final approval of this version to be published.

MP conceptualised the study, assisted with its design and the interpretation of data, critically reviewed and edited the draft article and gave final approval of this version to be published.

BCR conceptualised the study, assisted with its design and the interpretation of data, critically reviewed and edited the draft article and gave final approval of this version to be published.

CBD conceptualised the study, assisted with its design and the interpretation of data, critically reviewed and edited the draft article and gave final approval of this version to be published.

SD assisted with the study design and the interpretation of data, critically reviewed and edited the draft article and gave final approval of this version to be published.

JPG assisted with the interpretation of data, critically reviewed and edited the draft article and gave final approval of this version to be published.

RAA assisted with the interpretation of data, critically reviewed and edited the draft article and gave final approval of this version to be published.

SW conceptualised the study, assisted with its design and the interpretation of data, critically reviewed and edited the draft article and gave final approval of this version to be published.

FUNDING

This research was funded by the National Institute for Health Research (NIHR), Health Services and Delivery Research Programme (ref 11/2003/58). Barnaby C. Reeves, Chiara Bucciarelli-Ducci and the Cardiac MRI Unit are also partly funded through the NIHR

For peer review only - http://bmjopen.bmj.com/site/about/guidelines.xhtml

Biomedical Research Centre at University Hospitals Bristol NHS Foundation Trust and the University of Bristol. The funder had no involvement in the study design; in the collection, analysis and interpretation of the data; in the writing of the paper; and in the decision to submit the paper for publication.

DISCLAIMER

The views expressed are those of the authors and not necessarily those of the NHS, the NIHR or the Department of Health and Social Care.

COMPETING INTERESTS

Chiara Bucciarelli-Ducci has received personal fees from Circle Cardiovascular Imaging. Barnaby C. Reeves reports former membership of the Health Technology Assessment Commissioning Board (up to 31 March 2016) and Health Technology Assessment Efficient Study Designs Board (October 2014 to December 2014). He also reports current membership of the Health Technology Assessment IP Methods Group and Systematic Reviews Programme Advisory Group (Systematic Reviews NIHR Cochrane Incentive Awards and Systematic Review Advisory Group). Beyond this, all authors have no competing interests, except support from the NIHR grant as detailed in the funding statement.

ETHICS

Since the health economic models were populated with data from secondary sources, ethical approval was not required for this study.

PATIENT CONSENT

Patient consent was not required.

DATA SHARING STATEMENT

No additional data available.

For peer review only

For peer review only - http://bmjopen.bmj.com/site/about/guidelines.xhtml

REFERENCES

1. Kumar A, Cannon CP. Acute coronary syndromes: diagnosis and management, part I. *Mayo Clin Proc* 2009;84(10):917-38.
2. Cardarelli F, Bellasi A, Ou FS, et al. Combined impact of age and estimated glomerular filtration rate on in-hospital mortality after percutaneous coronary intervention for acute myocardial infarction (from the American College of Cardiology National Cardiovascular Data Registry). *Am J Cardiol* 2009;103(6):766-71.
3. Goldstein JA, Demetriou D, Grines CL, et al. Multiple complex coronary plaques in patients with acute myocardial infarction. *N Engl J Med* 2000;343(13):915-22.
4. Lee JH, Park HS, Chae SC, et al. Predictors of six-month major adverse cardiac events in 30-day survivors after acute myocardial infarction (from the Korea Acute Myocardial Infarction Registry). *Am J Cardiol* 2009;104(2):182-9.
5. Rasoul S, Ottervanger JP, de Boer MJ, et al. Predictors of 30-day and 1-year mortality after primary percutaneous coronary intervention for ST-elevation myocardial infarction. *Coron Artery Dis* 2009;20(6):415-21.
6. Toma M, Buller CE, Westerhout CM, et al. Non-culprit coronary artery percutaneous coronary intervention during acute ST-segment elevation myocardial infarction: insights from the APEX-AMI trial. *Eur Heart J* 2010;31(14):1701-7.
7. Vlaar PJ, Mahmoud KD, Holmes DR, Jr., et al. Culprit vessel only versus multivessel and staged percutaneous coronary intervention for multivessel disease in patients presenting with ST-segment elevation myocardial infarction: a pairwise and network meta-analysis. *J Am Coll Cardiol* 2011;58(7):692-703.
8. Larsen AI, Galbraith PD, Ghali WA, et al. Characteristics and outcomes of patients with acute myocardial infarction and angiographically normal coronary arteries. *Am J Cardiol* 2005;95(2):261-3.
9. Meryon I, Patel N, Millane T, et al. Normal coronary angiography and primary percutaneous coronary intervention for ST elevation myocardial infarction: a literature review and audit findings. *Int J Clin Pract* 2010;64(9):1245-51.
10. Roongsritong C, Warraich I, Bradley C. Common causes of troponin elevations in the absence of acute myocardial infarction: incidence and clinical significance. *Chest* 2004;125(5):1877-84.
11. Sarda L, Colin P, Boccara F, et al. Myocarditis in patients with clinical presentation of myocardial infarction and normal coronary angiograms. *J Am Coll Cardiol* 2001;37(3):786-92.
12. Dokainish H, Pillai M, Murphy SA, et al. Prognostic implications of elevated troponin in patients with suspected acute coronary syndrome but no critical epicardial coronary disease: a TACTICS-TIMI-18 substudy. *J Am Coll Cardiol* 2005;45(1):19-24.
13. Assomull RG, Lyne JC, Keenan N, et al. The role of cardiovascular magnetic resonance in patients presenting with chest pain, raised troponin, and unobstructed coronary arteries. *Eur Heart J* 2007;28(10):1242-9.
14. Codreanu A, Djaballah W, Angioi M, et al. Detection of myocarditis by contrast-enhanced MRI in patients presenting with acute coronary syndrome but no coronary stenosis. *J Magn Reson Imaging* 2007;25(5):957-64.
15. Laissy JP, Hyafil F, Feldman LJ, et al. Differentiating acute myocardial infarction from myocarditis: diagnostic value of early- and delayed-perfusion cardiac MR imaging. *Radiology* 2005;237(1):75-82.

For peer review only - http://bmjopen.bmj.com/site/about/guidelines.xhtml

16. Leurent G, Langella B, Fougerou C, et al. Diagnostic contributions of cardiac magnetic resonance imaging in patients presenting with elevated troponin, acute chest pain syndrome and unobstructed coronary arteries. *Arch Cardiovasc Dis* 2011;104(3):161-70.
17. Stensaeth KH, Fossum E, Hoffmann P, et al. Clinical characteristics and role of early cardiac magnetic resonance imaging in patients with suspected ST-elevation myocardial infarction and normal coronary arteries. *Int J Cardiovasc Imaging* 2011;27(3):355-65.
18. Antony R, Daghem M, McCann GP, et al. Cardiovascular magnetic resonance activity in the United Kingdom: a survey on behalf of the British Society of Cardiovascular Magnetic Resonance. *J Cardiovasc Magn Reson* 2011;13:57.
19. Ibanez B, James S, Agewall S, et al. 2017 ESC Guidelines for the management of acute myocardial infarction in patients presenting with ST-segment elevation: The Task Force for the management of acute myocardial infarction in patients presenting with ST-segment elevation of the European Society of Cardiology (ESC). *Eur Heart J* 2018;39(2):119-77.
20. Lockie T, Ishida M, Perera D, et al. High-resolution magnetic resonance myocardial perfusion imaging at 3.0-Tesla to detect hemodynamically significant coronary stenoses as determined by fractional flow reserve. *J Am Coll Cardiol* 2011;57(1):70-5.
21. Watkins S, McGeoch R, Lyne J, et al. Validation of magnetic resonance myocardial perfusion imaging with fractional flow reserve for the detection of significant coronary heart disease. *Circulation* 2009;120(22):2207-13.
22. Bhattacharyya S, Chehab O, Khattar R, et al. Stress echocardiography in clinical practice: a United Kingdom National Health Service Survey on behalf of the British Society of Echocardiography. *European Heart Journal - Cardiovascular Imaging* 2014;15(2):158-63.
23. Brierley R, Pufulete M, Harris J, et al. Developing a UK registry to investigate the role of cardiovascular magnetic resonance (CMR) in patients who activate the primary percutaneous coronary intervention (PPCI) pathway: a multicentre, feasibility study linking routinely collected electronic patient data. *BMJ Open* 2018;8(3):e018987.
24. National Institute for Health and Care Excellence (NICE). Guide to the Methods of Technology Appraisal 2013. London: NICE 2013.
25. Department of Health. National Schedule of Reference Costs 2015-16. London: Department of Health 2016.
26. Joint Formulary Committee. British National Formulary No. 73. London: British Medical Association and Royal Pharmaceutical Society of Great Britian 2017.
27. The Center for the Evaluation of Value and Risk in Health Tufts Medical Center. Cost-Effectiveness Analysis (CEA) Registry. URL: http://healtheconomics.tuftsmedicalcenter.org/cear4/Home.aspx. (accessed 06/04/2017).
28. Dastidar AG, Rodrigues JC, Baritussio A, et al. MRI in the assessment of ischaemic heart disease. *Heart* 2016;102(3):239-52.
29. Pasupathy S, Air T, Dreyer RP, et al. Systematic review of patients presenting with suspected myocardial infarction and nonobstructive coronary arteries. *Circulation* 2015;131(10):861-70.
30. Tornvall P, Gerbaud E, Behaghel A, et al. Myocarditis or "true" infarction by cardiac magnetic resonance in patients with a clinical diagnosis of myocardial infarction without obstructive coronary disease: A meta-analysis of individual patient data. *Atherosclerosis* 2015;241(1):87-91.

For peer review only

For peer review only - http://bmjopen.bmj.com/site/about/guidelines.xhtml

31. Gurunathan S, Young G, Parsons G, et al. 132 Diagnostic Accuracy of Stress Echocardiography Compared with Invasive Coronary Angiography with Fractional Flow Reserve for The Diagnosis of Haemodynamically Significant Cad in Patients with Known or Suspected CAD. *Heart* 2016;102(Suppl 6):A94-A95.
32. Dastidar AG, Rodrigues JCL, Johnson TW, et al. Myocardial Infarction With Nonobstructed Coronary Arteries: Impact of CMR Early After Presentation. *JACC Cardiovasc Imaging* 2017;10(10 Pt A):1204-06.
33. Tonino PA, Fearon WF, De Bruyne B, et al. Angiographic versus functional severity of coronary artery stenoses in the FAME study fractional flow reserve versus angiography in multivessel evaluation. *J Am Coll Cardiol* 2010;55(25):2816-21.
34. Smits PC, Abdel-Wahab M, Neumann FJ, et al. Fractional Flow Reserve-Guided Multivessel Angioplasty in Myocardial Infarction. *N Engl J Med* 2017;376(13):1234-44.
35. Chopard R, Jehl J, Dutheil J, et al. Evolution of acute coronary syndrome with normal coronary arteries and normal cardiac magnetic resonance imaging. *Arch Cardiovasc Dis* 2011;104(10):509-17.
36. Gerbaud E, Harcaut E, Coste P, et al. Cardiac magnetic resonance imaging for the diagnosis of patients presenting with chest pain, raised troponin, and unobstructed coronary arteries. *Int J Cardiovasc Imaging* 2012;28(4):783-94.
37. Kang WY, Jeong MH, Ahn YK, et al. Are patients with angiographically near-normal coronary arteries who present as acute myocardial infarction actually safe? *Int J Cardiol* 2011;146(2):207-12.
38. Pathik B, Raman B, Mohd Amin NH, et al. Troponin-positive chest pain with unobstructed coronary arteries: incremental diagnostic value of cardiovascular magnetic resonance imaging. *Eur Heart J Cardiovasc Imaging* 2016;17(10):1146-52.
39. Lindahl B, Baron T, Erlinge D, et al. Medical Therapy for Secondary Prevention and Long-Term Outcome in Patients With Myocardial Infarction With Nonobstructive Coronary Artery Disease. *Circulation* 2017;135(16):1481-89.
40. Aasa M, Henriksson M, Dellborg M, et al. Cost and health outcome of primary percutaneous coronary intervention versus thrombolysis in acute ST-segment elevation myocardial infarction-Results of the Swedish Early Decision reperfusion Study (SWEDES) trial. *Am Heart J* 2010;160(2):322-8.
41. Nam J, Briggs A, Layland J, et al. Fractional flow reserve (FFR) versus angiography in guiding management to optimise outcomes in non-ST segment elevation myocardial infarction (FAMOUS-NSTEMI) developmental trial: cost-effectiveness using a mixed trial- and model-based methods. *Cost Eff Resour Alloc* 2015;13:19.
42. Palmer S, Sculpher M, Philips Z, et al. A cost-effectiveness model comparing alternative management strategies for the use of glycoprotein IIb/IIIa antagonists in non-st-elevation acute coronary syndrome. Report to the National Institute for Clinical Excellence 2002.
43. Daniel M, Agewall S, Caidahl K, et al. Effect of Myocardial Infarction With Nonobstructive Coronary Arteries on Physical Capacity and Quality-of-Life. *Am J Cardiol* 2017;120(3):341-46.
44. MR-INFORM: Stress Perfusion Imaging to Guide the Management of Stable CAD Patients. American College of Cardiology Annual Scientific Session; 2017 17/03/2017; Washington, DC.

For peer review only - http://bmjopen.bmj.com/site/about/guidelines.xhtml

FIGURE LEGENDS

Figure 1. Model 1 Structure - Patients with multi-vessel disease. Patient pathways for FFR and stress ECHO are identical to those for CMR. CMR, Cardiovascular Magnetic Resonance; echo, Echocardiography; FFR; Fractional flow reserve; MACE, Major adverse cardiac events.

Figure 2. Model 2 Structure - Patients with unobstructed coronaries. CMR, Cardiovascular Magnetic Resonance; echo, Echocardiography; MACE, Major adverse cardiac events; MI, Myocardial infarction.

Figure 3. (A) Plot of the 1000 simulated cost and QALY differences from the PSA for CMR versus stress ECHO; (B) Plot of the 1000 simulated cost and QALY differences from the PSA for FFR versus stress ECHO; (C) Plot of the 1000 simulated cost and QALY differences from the PSA for standard ECHO and CMR versus standard ECHO. CMR, Cardiovascular Magnetic Resonance; ECHO, Echocardiography; FFR; Fractional flow reserve; PSA, Probabilistic sensitivity analyses; QALY, Quality-adjusted life years.

For peer review only

For peer review only - http://bmjopen.bmj.com/site/about/guidelines.xhtml

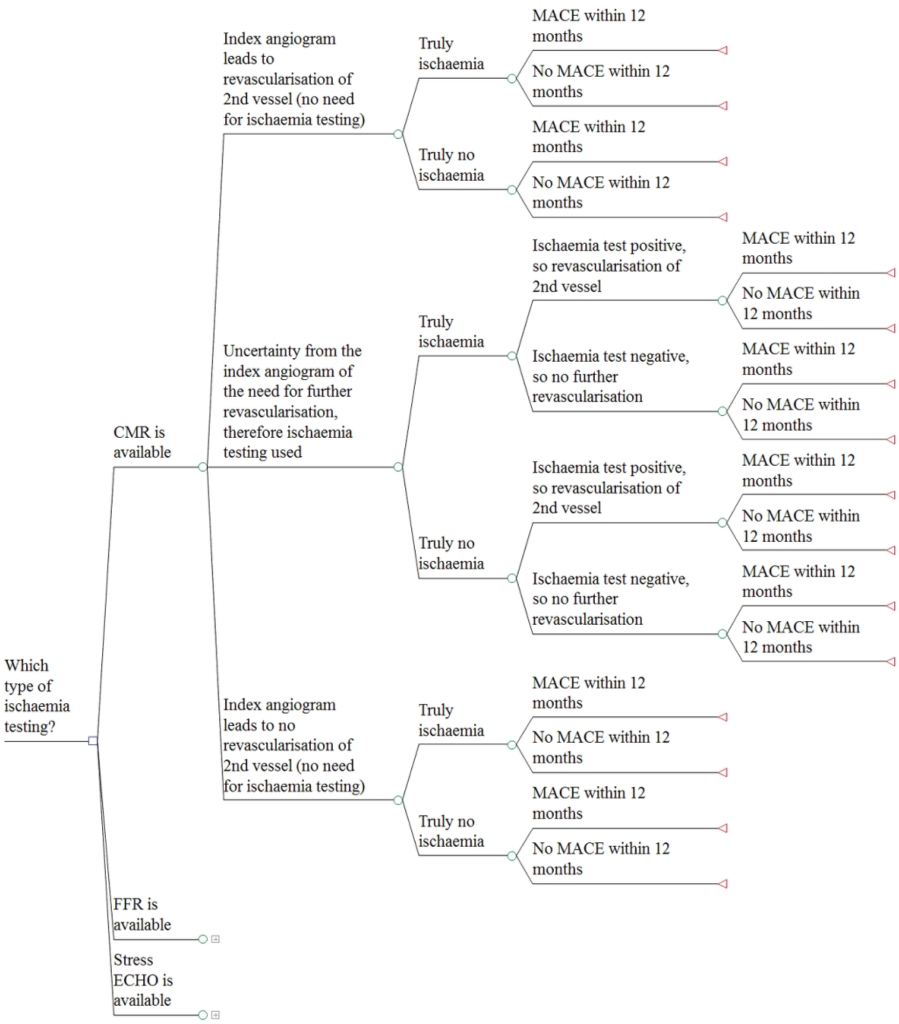

Model 1 Structure - Patients with multi-vessel disease.

123x127mm (300 x 300 DPI)

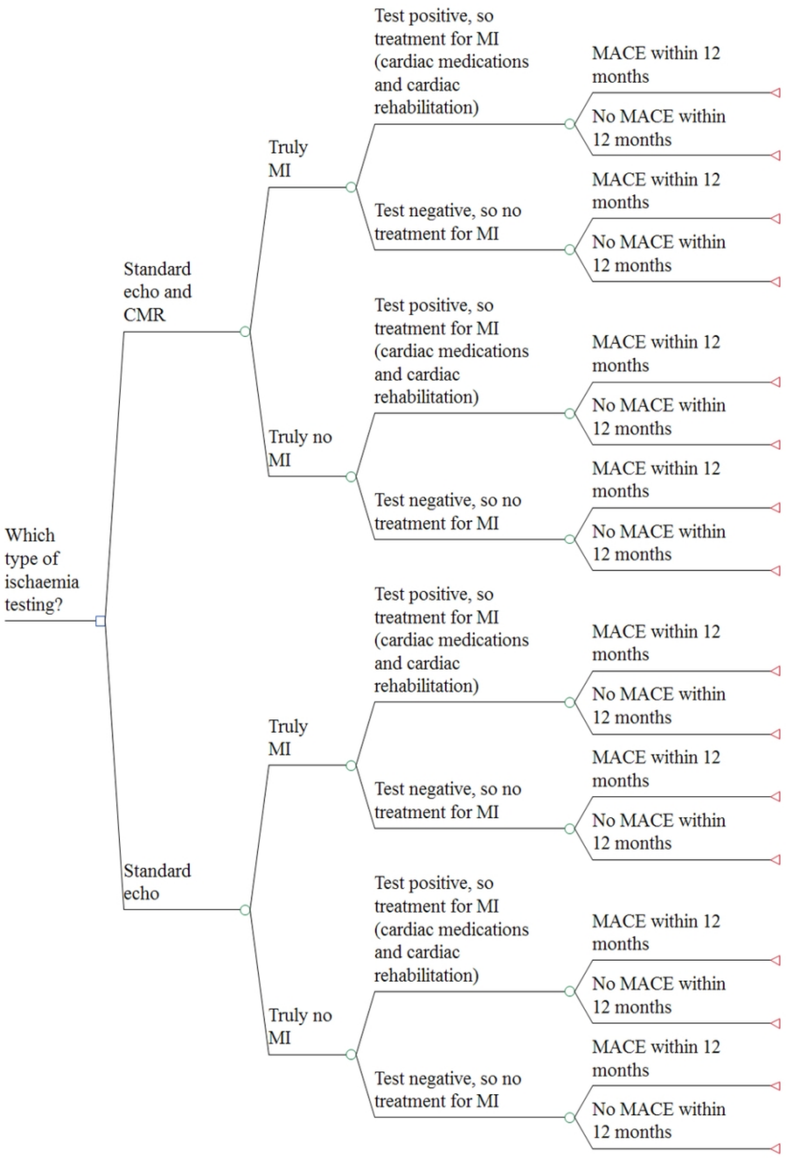

Model 2 Structure - Patients with unobstructed coronaries.

109x127mm (300 x 300 DPI)

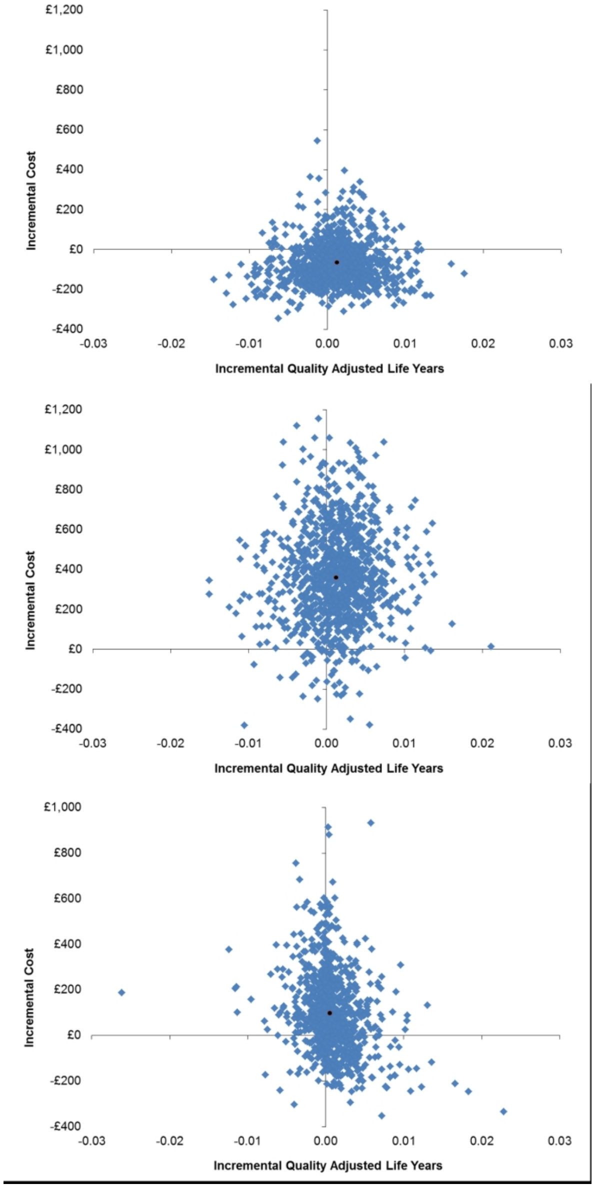

(A) Plot of the 1000 simulated cost and QALY differences from the PSA for CMR versus stress ECHO; (B) Plot of the 1000 simulated cost and QALY differences from the PSA for FFR versus stress ECHO; (C) Plot of the 1000 simulated cost and QALY differences from the PSA for standard ECHO and CMR versus standard ECHO.

106x216mm (300 x 300 DPI)

SUPPLEMENTARY INFORMATION

Table of Contents

Appendix A – Model parameters, sources and assumptions for model 1 (multi-vessel disease) 2

Appendix B – Model parameters, sources and assumptions for model 2 (unobstructed coronary arteries) 6

Appendix C – Deterministic one-way sensitivity analyses for model 1 (multi-vessel disease) 9

Appendix D – Deterministic one-way sensitivity analyses for model 2 (unobstructed coronary arteries) 11

References 12

For peer review only

For peer review only - http://bmjopen.bmj.com/site/about/guidelines.xhtml

APPENDIX A – MODEL PARAMETERS, SOURCES AND ASSUMPTIONS FOR MODEL 1 (MULTI-VESSEL DISEASE)

**Testing and clinical parameters**			
Branch of model	Base-case value (Positive/total)	Distribution for PSA	Source
For each ischaemia testing option			
Probability index angiogram leads to revascularisation of second vessel	0.30 (30/100)*	Dirichlet	Expert opinion within the PIPA study team
Probability of uncertainty following index angiogram and need for ischaemia testing	0.60 (60/100)*		
Probability index angiogram leads to no revascularisation of second vessel	0.10 (10/100)*		
Index angiogram leads to revascularisation of second vessel			
Probability of true ischaemia	0.84 (598/709)	Beta	FAME[1]
Probability of MACE by 12 months | true ischaemia	0.09 (18/202)†	Beta	Smits 2017[2]
Probability of MACE by 12 months | truly no ischaemia	0.13 (54/432)†	Beta	Smits 2017[2]
Uncertainty following index angiogram and need for ischaemia testing			
Probability of true ischaemia	0.35 (218/620)	Beta	FAME[1]
Index angiogram leads to no revascularisation of second vessel			
Probability of true ischaemia	0.35 (218/620)	Beta	FAME[1]
Probability of MACE by 12 months | true ischaemia	0.31 (71/231)†	Beta	Smits 2017[2]
Probability of MACE by 12 months | truly no ischaemia	0.13 (54/432)†	Beta	Smits 2017[2]
CMR or FFR			
Uncertainty following index angiogram and need for ischaemia testing, True ischaemia			
Probability of ischaemia test positive and revascularisation (sensitivity of test)	1§	Beta	
Probability of MACE by 12 months | revascularisation	0.09 (18/202)†	Beta	Smits 2017[2]
Probability of MACE by 12 months | no revascularisation	0.31 (71/231)†#	Beta	Smits 2017[2]
Uncertainty following index angiogram and need for ischaemia testing, Truly no ischaemia			
Probability of ischaemia test positive and revascularisation (1 minus specificity of test)	0§	Beta	
Probability of MACE by 12 months | revascularisation	0.13 (54/432)†#	Beta	Smits 2017[2]

For peer review only - http://bmjopen.bmj.com/site/about/guidelines.xhtml

Probability of MACE by 12 months | no revascularisation	0.13 (54/432)†	Beta	Smits 2017[2]
Stress ECHO			
Uncertainty following index angiogram and need for ischaemia testing, True ischaemia			
Probability of ischaemia test positive and revascularisation (sensitivity of test)	0.70 (32/46)	Beta	Gurunathan 2016[3]
Probability of MACE by 12 months | revascularisation	0.09 (18/202)†	Beta	Smits 2017[2]
Probability of MACE by 12 months | no revascularisation	0.31 (71/231)†	Beta	Smits 2017[2]
Uncertainty following index angiogram and need for ischaemia testing, Truly no ischaemia			
Probability of ischaemia test positive and revascularisation (1 minus specificity of test)	0.23 (39/171)	Beta	Gurunathan 2016[3]
Probability of MACE by 12 months | revascularisation	0.13 (54/432)†	Beta	Smits 2017[2]
Probability of MACE by 12 months | no revascularisation	0.13 (54/432)†	Beta	Smits 2017[2]
**Cost parameters**			
Resource	Unit cost (SE)**	Distribution for PSA	Source
Revascularisation of second vessel (assumed to be PCI)	£2,682 (533)	Gamma	NHS Reference Costs 2015/16.[4] Elective Inpatient. Weighted average of codes EY40 and EY41, Standard / Complex Percutaneous Transluminal Coronary Angioplasty.
CMR	£264 (154)	Gamma	NHS Reference Costs 2015/16.[4] Diagnostic Imaging. Weighted average of all Cardiac Magnetic Resonance Imaging Scan codes.
Angiogram and FFR	£1,340 (374)	Gamma	NHS Reference Costs 2015/16.[4] Day case. Weighted average of code EY42, Complex Cardiac Catheterisation.
Angiogram, FFR and revascularisation (as a single catheter laboratory admission)	£2,971 (594)	Gamma	Cost of revascularisation above, and angiogram and FFR above, minus the cost of an angiogram, £1,051 (NHS Reference Costs 2015/16.[4] Day case. Weighted average of code EY43, Standard Cardiac Catheterisation)
Stress ECHO	£182 (3)	Gamma	NHS Reference Costs 2015/16.[4] Outpatient procedures. Cardiology. EY50Z, Complex Echocardiogram.
MACE	£3,251 (650)	Gamma	The proportion of each component of MACE was taken from Smits, 2017,[2] and a weighted average of the costs of

For peer review only

			each component was calculated, based on the unit costs below.
Revascularisation (PCI)	£2,682		As above
Revascularisation (CABG)	£10,944		NHS Reference Costs 2015/16.[4] Elective Inpatient. Weighted average of codes ED26, ED27, ED28 Complex/ Major/ Standard Coronary Artery Bypass Graft
Myocardial infarction (MI)	£2,177		NHS Reference Costs 2015/16.[4] Non-elective long stay. Weighted average of code EB10A, Actual or Suspected Myocardial Infarction.
Stroke	£3,723		NHS Reference Costs 2015/16.[4] Non-elective long stay. Weighted average of code AA22, Cerebrovascular Accident, Nervous System Infections or Encephalopathy.
Death	£0		
Cardiac rehabilitation	£364 (73)	Gamma	Assume offered eight sessions, but only 50% update, so cost four sessions (one first and three follow up appointments, individual costs below)
First appointment	£97		NHS Reference Costs 2015/16.[4] Consultant Led. 327, Cardiac Rehabilitation. Non-Admitted Face to Face Attendance, First.
Follow up appointment	£89		NHS Reference Costs 2015/16.[4] Consultant Led. 327, Cardiac Rehabilitation. Non-Admitted Face to Face Attendance, Follow-Up.
Cardiac medications for treatment of MI (all once daily by mouth). Assumed given for 12 months (or 6 months for patients who die).			
Aspirin (75mg)	£0.04		BNF 73, March 2017[5]
Prasugrel (5mg)	£1.70		BNF 73, March 2017[5]
Atorvastatin (80mg)	£0.07		BNF 73, March 2017[5]
Bisoprolol (2.5mg)	£0.02		BNF 73, March 2017[5]
Ramipril (2.5mg)	£0.04		BNF 73, March 2017[5]
Total daily cost	£1.87		
Total annual cost	£683 (137)	Gamma	
Outpatient follow up appointment (assumed to occur at 4-6 weeks and 6 months post-discharge)	£122 (22)	Gamma	NHS Reference Costs 2015/16.[4] Consultant led appointments. Non-Admitted Face to Face Attendance, Follow-Up. Cardiology.
Additional healthcare costs to one year	£2221 (444)	Gamma	Office of Health Economics estimate of the annual cost per person of NHS care inflated to 2015/16 prices using the

For peer review only - http://bmjopen.bmj.com/site/about/guidelines.xhtml

			hospital and community health services inflation index[6, 7] For patients who die, costs are assumed to be half of this (£1111).
Additional healthcare costs to one year for those with MACE	£263 (53)	Gamma	MI and stroke were assumed to be associated with continuing care costs post discharge. Costs from Greenhalgh (2011),[8] were inflated to 2015/16 prices.[6] Separate costs were provided for disabling and non-disabling stroke; according to Davies (2006) 58% of strokes are disabling,[9] and this percentage was used to weight the two continuing care costs for stroke. Additional costs to one year associated with MACE were calculated by weighting estimates of the annual costs of MI and stroke by the number of patients with these complications across the number of patients with MACE, from Smits (2017).[2]
**Quality-adjusted life years**			
Patient group	Mean (SE) †† QALYs to one year	Distribution for PSA	Source
No revascularisation of a second vessel and MACE	0.686 (0.09)	Beta	Calculations and sources described in the text
No revascularisation of a second vessel and no MACE	0.768 (0.09)	Beta	
Revascularisation of a second vessel and MACE	0.684 (0.09)	Beta	
Revascularisation of a second vessel and no MACE	0.766 (0.09)	Beta	

* To incorporate uncertainty around the percentage of patients in each of these three groups estimated by experts on the study team, it was assumed that experts were estimating the number of patients out of 100 in each group.
† For further information on the estimates of MACE, please see the final paragraph under ‘Model probabilities’.
§ In the base case analyses, CMR and FFR are assumed to have perfect sensitivity and specificity, but these parameters are incorporated into the model so that alternative assumptions can be tested in sensitivity analyses.
# These probabilities are not required in the base case analysis (since the probability in the preceding branch is zero), but are included here as they are required in sensitivity analyses.
** NHS Reference Costs provide upper and lower quartiles around mean estimates; the difference between the mean and the upper quartile was captured, and used as a crude estimate of standard error in the PSA. Where this was not possible, the standard error was assumed to be 20% of the mean cost.
†† A standard error of 0.09 was assumed, based on a previous study.[10]

CABG, Coronary Artery Bypass Graft; CMR, Cardiovascular Magnetic Resonance; ECHO, Echocardiography; FFR; Fractional flow reserve; MACE, Major adverse cardiac events; MI, Myocardial infarction; PCI, Percutaneous coronary intervention; PSA, Probabilistic sensitivity analysis; QALYs, Quality-adjusted life years; SE, Standard error

For peer review only - http://bmjopen.bmj.com/site/about/guidelines.xhtml

APPENDIX B – MODEL PARAMETERS, SOURCES AND ASSUMPTIONS FOR MODEL 2 (UNOBSTRUCTED CORONARY ARTERIES)

**Testing and clinical parameters**			
Branch of model	Base-case value (Positive/total)	Distribution for PSA	Source
For both arms			
Probability of truly having had an myocardial infarction (MI) (prevalence)	0.24 (429/1801)	Beta	Pasupathy 2015[11]
Standard ECHO and CMR			
Probability of ischaemia test positive and treatment for MI | MI (sensitivity of test)	1*	Beta	
Probability of MACE within 12 months | treatment for MI	0.21†	Beta	Kang 2011,[12] Pathik 2016[13]
Probability of MACE within 12 months | no treatment for MI	0.26†§	Beta	Kang 2011,[12] Pathik 2016,[13] Lindahl 2017[14]
Probability of ischaemia test positive and treatment for MI | no MI (1 minus specificity of test)	0*	Beta	
Probability of MACE within 12 months | treatment for MI	0.03†§	Beta	Kang 2011,[12] Pathik 2016[13]
Probability of MACE within 12 months | no treatment for MI	0.03†	Beta	Kang 2011,[12] Pathik 2016[13]
Standard ECHO			
Probability of ischaemia test positive and treatment for MI | MI (sensitivity of test)	0.47 (25/53)#	Beta	Dastidar 2017[15]
Probability of MACE within 12 months | treatment for MI	0.21†	Beta	Kang 2011,[12] Pathik 2016[13]
Probability of MACE within 12 months | no treatment for MI	0.26†	Beta	Kang 2011,[12] Pathik 2016,[13] Lindahl 2017[14]
Probability of ischaemia test positive and treatment for MI | no MI (1 minus specificity of test)	0.62 (93/151)#	Beta	Dastidar 2017[15]
Probability of MACE within 12 months | treatment for MI	0.03†	Beta	Kang 2011,[12] Pathik 2016[13]
Probability of MACE within 12 months | no treatment for MI	0.03†	Beta	Kang 2011,[12] Pathik 2016[13]

For peer review only - http://bmjopen.bmj.com/site/about/guidelines.xhtml

Cost parameters			
Resource	Unit cost (SE)**	Distribution for PSA	Source
Standard ECHO	£72 (22)	Gamma	NHS Reference Costs 2015/16.[4] Diagnostic Imaging. Outpatient. RD51A, Simple Echocardiogram, 19 years and over.
CMR	£264 (154)	Gamma	NHS Reference Costs 2015/16.[4] Diagnostic Imaging. Weighted average of all Cardiac Magnetic Resonance Imaging Scan codes.
Cardiac rehabilitation	£364 (73)	Gamma	Assume offered eight sessions, but only 50% update, so cost four sessions (one first and three follow up appointments, individual costs below)
First appointment	£97		NHS Reference Costs 2015/16.[4] Consultant Led. Cardiac Rehabilitation. Non-Admitted Face to Face Attendance, First.
Follow up appointment	£89		NHS Reference Costs 2015/16.[4] Consultant Led. Cardiac Rehabilitation. Non-Admitted Face to Face Attendance, Follow-Up.
Cardiac medications for treatment of MI (all once daily by mouth)			
Aspirin (75mg)	£0.04		BNF 73, March 2017[5]
Clopidogrel (75mg)	£0.05		BNF 73, March 2017[5]
Atorvastatin (80mg)	£0.07		BNF 73, March 2017[5]
Bisoprolol (2.5mg)	£0.02		BNF 73, March 2017[5]
Ramipril (2.5mg)	£0.04		BNF 73, March 2017[5]
Total daily cost	£0.22		
Total annual cost	£80 (16)	Gamma	
Treatment for non-MI cause (cardiac medications) – total annual cost	£40 (8)	Gamma	Assume half of these patients are taken off cardiac medications above (these medications are given for cardiomyopathy but not for myocarditis)
Outpatient follow up appointment (assumed to occur at 4-6 weeks post-discharge)	£122 (22)	Gamma	NHS Reference Costs 2015/16.[4] Consultant led appointments. Non-Admitted Face to Face Attendance, Follow-Up. Cardiology.
MACE	£2,808 (562)	Gamma	The proportion of each component of MACE was taken from Pathik,[13] and a weighted average of the costs of each component was calculated, based on the unit costs provided in Appendix A. Given the small number of MACE

For peer review only - http://bmjopen.bmj.com/site/about/guidelines.xhtml

			events, calculations were based on MI and non-MI patients combined.
Additional healthcare costs to one year	£2221 (444)	Gamma	Office of Health Economics estimate of the annual cost per person of NHS care inflated to 2015/16 prices using the hospital and community health services inflation index[6, 7] For patients who die, costs are assumed to be half of this (£1111).
Additional healthcare costs to one year for those with MACE	£1602 (320)	Gamma	MI and stroke were assumed to be associated with continuing care costs post discharge. Costs from Greenhalgh (2011),[8] were inflated to 2015/16 prices.[6] Separate costs were provided for disabling and non-disabling stroke; according to Davies (2006) 58% of strokes are disabling,[9] and this percentage was used to weight the two continuing care costs for stroke. Additional costs to one year associated with MACE were calculated by weighting estimates of the annual costs of MI and stroke by the number of patients with these complications across the number of patients with MACE, from Pathik (2017).[13]
**Quality-adjusted life years**			
Patient group	Mean (SE)†† QALYs to one year	Distribution for PSA	Source
MACE within 12 months	0.686 (0.09)	Beta	Calculations and sources described in the text
No MACE within 12 months	0.768 (0.09)	Beta	

* In the base case analyses, standard ECHO combined with CMR is assumed to have perfect sensitivity and specificity, but these parameters are incorporated into the model so that alternative assumptions can be tested in sensitivity analyses.
† A standard error of 0.10 was assumed for the PSA.
§ These probabilities are not required in the base case analysis (since the probability in the preceding branch is zero), but are included here as they are required in sensitivity analyses.
# For further information on how estimates of sensitivity and specificity for standard ECHO were obtained, please see the final paragraph under 'Model probabilities'.
** NHS Reference Costs provide upper and lower quartiles around mean estimates; the difference between the mean and the upper quartile was captured, and used as a crude estimate of standard error in the PSA. Where this was not possible, the standard error was assumed to be 20% of the mean cost.
†† A standard error of 0.09 was assumed, based on a previous study.[10]

CMR, Cardiovascular Magnetic Resonance; ECHO, Echocardiography; MACE, Major adverse cardiac events; MI, Myocardial infarction; PSA, Probabilistic sensitivity analysis; QALYs, Quality-adjusted life years

For peer review only - http://bmjopen.bmj.com/site/about/guidelines.xhtml

APPENDIX C – DETERMINISTIC ONE-WAY SENSITIVITY ANALYSES FOR MODEL 1 (MULTI-VESSEL DISEASE)

SA	Parameter varied	Base case	Alternative strategies for sensitivity analysis
1	Probabilities associated with decision on revascularisation from index angiogram:<br>Probability index angiogram leads to revascularisation of second vessel<br>Probability of uncertainty following index angiogram and need for ischaemia testing<br>Probability index angiogram leads to no revascularisation of second vessel	Based on expert opinion:<br>0.30 (30/100)<br>0.60 (60/100)<br>0.10 (10/100)	Based on PIPA data:<br>0.11 (78/717)<br>0.15 (111/717)<br>0.74 (528/717)
2a	Sensitivity of CMR	1	0.8 (SE 0.1)
2b	Specificity of CMR	1	0.8 (SE 0.1)
2c	Sensitivity and specificity of CMR (labelled test accuracy in manuscript Table 1)	1 and 1	0.8 (SE 0.1) for each
3a	Sensitivity of FFR	1	0.8 (SE 0.1)
3b	Specificity of FFR	1	0.8 (SE 0.1)
3c	Sensitivity and specificity of FFR (labelled test accuracy in manuscript Table 1)	1 and 1	0.8 (SE 0.1) for each
4a	Sensitivity of stress ECHO	0.70	0.791 (SE 0.008) [54]
4b	Specificity of stress ECHO	0.77 (reported 1-specificity =0.23)	1-specificity 0.129 (SE 0.007) [54]
4c	Sensitivity and specificity of stress ECHO (labelled test accuracy in manuscript Table 1)	0.70 and 0.77 (reported 1-specificity =0.23)	Sensitivity: 0.791 (SE 0.008) and 1-specificity 0.129 (SE 0.007) [54]
5	Probability of MACE for truly ischaemic patients who had a second revascularisation, truly ischaemic patients who did not have a second revascularisation, and truly not ischaemic patients whether or not they had a second revascularisation	0.09, 0.31, 0.13	All ±0.05
6	Cost of CMR	£264	± 20%
7	Cost of angiogram and FFR	£1340	± 20%

For peer review only - http://bmjopen.bmj.com/site/about/guidelines.xhtml

8	QALYs for patients with MACE with and without revascularisation of a second vessel	0.684, 0.686	Base case -0.2 (0.484, 0.486)
9	QALYs for patients with no MACE with and without revascularisation of a second vessel	0.766, 0.768	Base case +0.2 (0.966, 0.968)

CMR, Cardiovascular Magnetic Resonance; ECHO, Echocardiography; FFR, Fractional flow reserve; MACE, Major adverse cardiac events; QALY, Quality-adjusted life years; SA, Sensitivity analysis; SE, Standard error

For peer review only

For peer review only - http://bmjopen.bmj.com/site/about/guidelines.xhtml

APPENDIX D – DETERMINISTIC ONE-WAY SENSITIVITY ANALYSES FOR MODEL 2 (UNOBSTRUCTED CORONARY ARTERIES)

SA	Parameter varied	Base case	Alternative strategies for sensitivity analysis
1a	Sensitivity of standard ECHO plus CMR	1	0.8 (SE 0.1)
1b	Specificity of standard ECHO plus CMR	1	0.8 (SE 0.1)
1c	Sensitivity and specificity of standard ECHO plus CMR (labelled test accuracy in manuscript Table 2)	1 and 1	0.8 (SE 0.1) for each
2a	Sensitivity of standard ECHO	0.47	0.7 (SE 0.1)
2b	Specificity of standard ECHO	0.38	0.7 (SE 0.1)
2c	Sensitivity and specificity of standard ECHO (labelled test accuracy in manuscript Table 2)	0.47 and 0.38	0.7 (SE 0.1) for each
3	Probability of MACE for patients who had a MI and treatment, for patients who had a MI and no treatment, and for patients without MI regardless of whether they had treatment (ratio of events in MI:non-MI group =4)*	0.21, 0.26, 0.03	0.18, 0.23, 0.05
4	Probability of MACE (ratio=2)	0.21, 0.26, 0.03	0.13, 0.17, 0.06
5	Probability of MACE (ratio=1)	0.21, 0.26, 0.03	0.08, 0.10, 0.08
6	Cost of CMR	£264	± 20%
7	Cost of additional healthcare costs to one year for patients with MACE - increase the proportion who die from 8% to 50%	£1602	£874
8	QALYs for patients with MACE	0.686	Base case -0.2 (0.486)
9	QALYs for patients with no MACE	0.768	Base case +0.2 (0.968)
10	QALYs for patients with MACE - increase the proportion who die from 8% to 50%	0.686	0.525

** For further information on this ratio, please see the Clinical parameters section of the manuscript.*
CMR, Cardiovascular Magnetic Resonance; ECHO, Echocardiography; MACE, Major adverse cardiac events; MI, Myocardial infarction; QALY, Quality-adjusted life years; SA, Sensitivity analysis; SE, Standard error

For peer review only - http://bmjopen.bmj.com/site/about/guidelines.xhtml

REFERENCES

1. Tonino PA, Fearon WF, De Bruyne B, et al. Angiographic versus functional severity of coronary artery stenoses in the FAME study fractional flow reserve versus angiography in multivessel evaluation. *J Am Coll Cardiol* 2010;55(25):2816-21.
2. Smits PC, Abdel-Wahab M, Neumann FJ, et al. Fractional Flow Reserve-Guided Multivessel Angioplasty in Myocardial Infarction. *N Engl J Med* 2017;376(13):1234-44.
3. Gurunathan S, Young G, Parsons G, et al. 132 Diagnostic Accuracy of Stress Echocardiography Compared with Invasive Coronary Angiography with Fractional Flow Reserve for The Diagnosis of Haemodynamically Significant Cad in Patients with Known or Suspected CAD. *Heart* 2016;102(Suppl 6):A94-A95.
4. Department of Health. National Schedule of Reference Costs 2015-16. London: Department of Health 2016.
5. Joint Formulary Committee. British National Formulary No. 73. London: British Medical Association and Royal Pharmaceutical Society of Great Britian 2017.
6. Curtis L, Burns A. Unit costs of health and social care 2016. Canterbury: Personal Social Services Research Unit, University of Kent 2016.
7. Hawe E, Cockcroft L. OHE guide to UK health and health care statistics. London: Office of Health Economics 2013.
8. Greenhalgh J, Bagust A, Boland A, et al. Clopidogrel and modified-release dipyridamole for the prevention of occlusive vascular events (review of Technology Appraisal No. 90): a systematic review and economic analysis. *Health Technol Assess* 2011;15(31):1-178.
9. Davies L, Brown TJ, Haynes S, et al. Cost-effectiveness of cell salvage and alternative methods of minimising perioperative allogeneic blood transfusion: a systematic review and economic model. *Health Technol Assess* 2006;10(44).
10. Palmer S, Sculpher M, Philips Z, et al. A cost-effectiveness model comparing alternative management strategies for the use of glycoprotein IIb/IIIa antagonists in non-st-elevation acute coronary syndrome. Report to the National Institute for Clinical Excellence 2002.
11. Pasupathy S, Air T, Dreyer RP, et al. Systematic review of patients presenting with suspected myocardial infarction and nonobstructive coronary arteries. *Circulation* 2015;131(10):861-70.
12. Kang WY, Jeong MH, Ahn YK, et al. Are patients with angiographically near-normal coronary arteries who present as acute myocardial infarction actually safe? *Int J Cardiol* 2011;146(2):207-12.
13. Pathik B, Raman B, Mohd Amin NH, et al. Troponin-positive chest pain with unobstructed coronary arteries: incremental diagnostic value of cardiovascular magnetic resonance imaging. *Eur Heart J Cardiovasc Imaging* 2016;17(10):1146-52.
14. Lindahl B, Baron T, Erlinge D, et al. Medical Therapy for Secondary Prevention and Long-Term Outcome in Patients With Myocardial Infarction With Nonobstructive Coronary Artery Disease. *Circulation* 2017;135(16):1481-89.
15. Dastidar AG, Rodrigues JCL, Johnson TW, et al. Myocardial Infarction With Nonobstructed Coronary Arteries: Impact of CMR Early After Presentation. *JACC Cardiovasc Imaging* 2017;10(10 Pt A):1204-06.

For peer review only - http://bmjopen.bmj.com/site/about/guidelines.xhtml

CHEERS Checklist

Items to include when reporting economic evaluations of health interventions

The **ISPOR CHEERS Task Force Report**, *Consolidated Health Economic Evaluation Reporting Standards (CHEERS)—Explanation and Elaboration: A Report of the ISPOR Health Economic Evaluations Publication Guidelines Good Reporting Practices Task Force,* provides examples and further discussion of the 24-item CHEERS Checklist and the CHEERS Statement. It may be accessed via the *Value in Health* or via the ISPOR Health Economic Evaluation Publication Guidelines – CHEERS: Good Reporting Practices webpage: http://www.ispor.org/TaskForces/EconomicPubGuidelines.asp

Section/item	Item No	Recommendation	Reported on page No/ line No
**Title and Abstract**			
Title	1	Identify the study as an economic evaluation or use more specific terms such as "cost-effectiveness analysis", and describe the interventions compared.	Title on page 1 uses the term 'cost-effectiveness analysis'. The intervention of interest, CMR imaging, is included in the title, but the comparators are not, since these differ in the two economic models, and there is not room to include both in the title.
Abstract	2	Provide a structured summary of objectives, perspective, setting, methods (including study design and inputs), results (including base case and uncertainty analyses), and conclusions.	Page 2.
**Introduction**			
Background and objectives	3	Provide an explicit statement of the broader context for the study.<br>Present the study question and its relevance for health policy or practice decisions.	Introduction, pages 4-5.<br><br>Introduction, pages 4-5; Methods: Overview and treatment strategies, final paragraph.
**Methods**			
Target population and subgroups	4	Describe characteristics of the base case population and subgroups analysed, including why they were chosen.	Introduction paragraphs 1 and 2, page 4; Methods: Overview and treatment strategies, page 5. No subgroups within these two populations considered.
Setting and location	5	State relevant aspects of the system(s) in which the decision(s) need(s) to be made.	Introduction paragraphs 1-3, page 4; Methods: Overview and treatment strategies, and Model structure, pages 6-7.

For peer review only

Study perspective	6	Describe the perspective of the study and relate this to the costs being evaluated.	Methods: Overview and treatment strategies, final sentence of section, page 5; and Methods: Cost parameter section, page 9 together with Appendices A&B in the Supplementary Information.
Comparators	7	Describe the interventions or strategies being compared and state why they were chosen.	Introduction paragraphs 2-3, page 4; Methods: Overview and treatment strategies, page 5.
Time horizon	8	State the time horizon(s) over which costs and consequences are being evaluated and say why appropriate.	Methods: Overview and treatment strategies, page 5.
Discount rate	9	Report the choice of discount rate(s) used for costs and outcomes and say why appropriate.	Methods: Economic analyses, lines 3-4 of section, page 7.
Choice of health outcomes	10	Describe what outcomes were used as the measure(s) of benefit in the evaluation and their relevance for the type of analysis performed.	Methods: Overview and treatment strategies, first sentence, page 5.
Measurement of effectiveness	11a	*Single study-based estimates:* Describe fully the design features of the single effectiveness study and why the single study was a sufficient source of clinical effectiveness data.	N/A
	11b	*Synthesis-based estimates:* Describe fully the methods used for identification of included studies and synthesis of clinical effectiveness data.	Methods: Identification of model parameters, paragraph 1, final sentence, page 7; Methods: Quality-adjusted life years, pages 9-10.
Measurement and valuation of preference based outcomes	12	If applicable, describe the population and methods used to elicit preferences for outcomes.	Methods: Quality-adjusted life years, lines 4-6 of section, page 9.
Estimating resources and costs	13a	*Single study-based economic evaluation:* Describe approaches used to estimate resource use associated with the alternative interventions. Describe primary or secondary research methods for valuing each resource item in terms	N/A

For peer review only

		of its unit cost. Describe any adjustments made to approximate to opportunity costs.	
	13b	*Model-based economic evaluation:* Describe approaches and data sources used to estimate resource use associated with model health states. Describe primary or secondary research methods for valuing each resource item in terms of its unit cost. Describe any adjustments made to approximate to opportunity costs.	Methods: Identification of model parameters, lines 2-3, page 7; Methods: Cost parameters, page 9, together with Appendices A&B in the Supplementary Information.
Currency, price date, and conversion	14	Report the dates of the estimated resource quantities and unit costs. Describe methods for adjusting estimated unit costs to the year of reported costs if necessary. Describe methods for converting costs into a common currency base and the exchange rate.	Methods: Economic analyses, line 2 of section, page 7. Appendices A&B in the Supplementary Information.
Choice of model	15	Describe and give reasons for the specific type of decision analytical model used. Providing a figure to show model structure is strongly recommended.	Methods: Overview and treatment strategies, and Model structure, pages 5-7; Figures 1 and 2.
Assumptions	16	Describe all structural or other assumptions underpinning the decision-analytical model.	Methods, pages 5-10; Appendices A&B in the Supplementary Information.
Analytical methods	17	Describe all analytical methods supporting the evaluation. This could include methods for dealing with skewed, missing, or censored data; extrapolation methods; methods for pooling data; approaches to validate or make adjustments (such as half cycle corrections) to a model; and methods for handling population heterogeneity and uncertainty.	Methods: Economic analyses, page 7; Methods: Base-case and sensitivity analyses, page 10; Appendices A&B in the Supplementary Information.
**Results**			
Study parameters	18	Report the values, ranges, references, and, if used, probability distributions for all parameters. Report reasons or sources for distributions used to represent uncertainty where appropriate. Providing a table to show the input values is strongly recommended.	Methods: Clinical parameters, pages 8-9; Methods: Base-case and sensitivity analyses, final paragraph of section, page 10; Appendices A&B in the Supplementary Information.

For peer review only

Incremental costs and outcomes	19	For each intervention, report mean values for the main categories of estimated costs and outcomes of interest, as well as mean differences between the comparator groups. If applicable, report incremental cost-effectiveness ratios.	Table 1, base case row, page 12; Table 2, base case row, page 16.
Characterising uncertainty	20a	*Single study-based economic evaluation:* Describe the effects of sampling uncertainty for the estimated incremental cost and incremental effectiveness parameters, together with the impact of methodological assumptions (such as discount rate, study perspective).	N/A
	20b	*Model-based economic evaluation:* Describe the effects on the results of uncertainty for all input parameters, and uncertainty related to the structure of the model and assumptions.	Results: Sensitivity analyses, pages 18-19; Tables 1 and 2; Appendices C&D in the Supplementary Information.
Characterising heterogeneity	21	If applicable, report differences in costs, outcomes, or cost-effectiveness that can be explained by variations between subgroups of patients with different baseline characteristics or other observed variability in effects that are not reducible by more information.	N/A
**Discussion**			
Study findings, limitations, generalisability, and current knowledge	22	Summarise key study findings and describe how they support the conclusions reached. Discuss limitations and the generalisability of the findings and how the findings fit with current knowledge.	Discussion, pages 19-21.
**Other**			
Source of funding	23	Describe how the study was funded and the role of the funder in the identification, design, conduct, and reporting of the analysis. Describe other non-monetary sources of support.	Funding section, pages 22-23.
Conflicts of interest	24	Describe any potential for conflict of interest of study contributors in accordance with journal policy. In	Competing interests section, page 23.

For peer review only

the absence of a journal policy, we recommend authors comply with International Committee of Medical Journal Editors recommendations.

For peer review only
